# The antibacterial effect of silver, zinc-oxide and combination of silver/ zinc oxide nanoparticles coating of orthodontic brackets (an in vitro study)

**DOI:** 10.1186/s12903-022-02263-6

**Published:** 2022-06-09

**Authors:** Noha K. Zeidan, Nagwa M. Enany, Gehad Genidy Mohamed, Eiman S. Marzouk

**Affiliations:** 1 Department of Orthodontics, Faculty of Dentistry, Alexandria University, Champollion St., Azarita, P. O. Box 21521, Alexandria, Egypt; 2 Chemistry Department, Faculty of Science, Cairo University, Giza, 12613 Egypt; 3 Nanoscience Department, Basic and Applied Science Institute, Egypt-Japan University of Science and Technology, New Borg El Arab, Alexandria, Egypt

**Keywords:** Orthodontics, Brackets, Coating, Nanoparticles, Silver, Zinc oxide

## Abstract

**Background:**

Preventive measures are essential during the length of orthodontic treatment to reduce the risk of decalcification and white spot lesions formation. With the evolution of procedures that enable coating of the orthodontic brackets using nanoparticles known for their good antibacterial activity, coating the brackets with nanoparticles of silver, zinc oxide and combination of silver and zinc oxide to evaluate their antibacterial effect in comparison to a control group without coating was carried out in this study.

**Methods:**

Four groups of 12 brackets each were included in the study. The coating procedure was carried out using physical vapor deposition. The antibacterial activity was tested on *Streptococcus mutans* and *Lactobacillus Acidophilus* using colony forming count. The antibacterial activity was evaluated immediately after coating and later after 3 months.

**Results:**

Brackets coated with combination of silver and zinc oxide nanoparticles had the highest ability on reduction of both *Streptococcus mutans* and *Lactobacillus Acidophilus* count followed by silver nanoparticles and then zinc oxide nanoparticles. No significant difference was found between the first and second antibacterial tests.

**Conclusion:**

The silver/zinc oxide nanoparticles coated brackets had the highest antibacterial effect in comparison to silver nanoparticles and zinc oxide nanoparticles individually coated brackets on *Streptococcus mutans* and *Lactobacillus acidophilus*, and all types of coatings showed enhanced antibacterial effect in comparison to the uncoated bracket. Coating of orthodontic brackets could be further assessed in clinical application to prevent decalcification.

**Supplementary Information:**

The online version contains supplementary material available at 10.1186/s12903-022-02263-6.

## Background

Improvement of esthetics is usually the primary goal for patients seeking orthodontic treatment [1, 2]. Attachment of fixed appliances on teeth challenges the patients with their oral hygiene measures leading to alterations in the oral environment [3, 4]. These variations are manifested as increase in areas of food particles retention, consequently leading to a rise in the number of microbial population and lowering of the dental cavity pH [5, 6]. The micro-organisms that increase on bonding and/ or banding were identified to be *Streptococcus mutans* (*S. mutans*) [7] and *Lactobacillus acidophilus* (*L. acidophilus*) [8, 9]. *S. mutans* were found to have an essential role in caries initiation [10, 11], while *L. acidophilus* contribute more in caries propagation [12]. The lowered pH causes demineralization of the enamel and impedes the process of remineralization [13]. This demineralization is manifested as white spot lesions (WSLs) and can lead to further cavitation. Therefore, the occurrence of decalcification and their appearance in the form of WSLs at the time of debonding is one of the main concerns for both the patient and the orthodontist [14].

Therefore, efforts were made to control or even prevent the development of WSLs. Starting from maintaining good oral hygiene measures that include teeth brushing using fluoridated toothpaste and rinsing with fluoride mouthwash [15, 16]. Moving to the application of varnishes [17, 18], resin materials containing antibacterial agent [19, 20] and using modified orthodontic elastomerics [21]. Recently, attempts included the addition of nanoparticles to orthodontic adhesives [22, 23], resin modified glass ionomer cements [24, 25], elastomerics [26] and coating of orthodontic brackets, wires and bands [27, 28].

Physical vapor deposition (PVD) is one of the coating techniques that is characterized by its sustainability, as coatings can be reproduced with more efficiency and higher purity than when using other techniques [29]. Thermal evaporation, which is a type of PVD is considered advantageous over magnetron sputtering in its better control over the produced film thickness and higher purity [30].

Nanoparticles are defined as insoluble materials of size smaller than 100 nm [31]. Because of its small size, it has a higher surface-to-volume ratio and a closer interaction with microbial membranes, resulting in a larger surface area of antimicrobial activity [32]. Several metals as silver, copper, gold, titanium and zinc have been used since ages to act as antimicrobial materials, where each of them has different properties and range of activity [33, 34].

Using silver, silver ions and silver compounds have been considered as antibacterial agents in biomedical applications [35]. In dental applications, nanoparticles of silver were proven to be an effective antimicrobial component when added to dental resin composites, and also when coated on orthodontic brackets and wires [22, 36, 37].

Zinc oxide nanoparticles (ZnO) was proven to be a good antibacterial agent [38]. Also, on coating orthodontic wires with ZnO, it was found to have a good antibacterial activity [39]. Although, the nanoparticles of silver (Ag) have displayed higher antimicrobial activity than ZnO nanoparticles [40], several studies have shown that Ag nanoparticles are cytotoxic and genotoxic to human cells [41, 42]. However, a composite of Ag and ZnO nanoparticles exhibited an improved antibacterial activity against *S. mutans* [25]*.*

Therefore, with the aim of benefiting from both ZnO nanoparticles and Ag nanoparticles, while reducing their individual cons which are the cytotoxicity of Ag nanoparticles [41, 42], its higher cost than ZnO nanoparticles [33], along with the reduced antibacterial effectiveness of ZnO nanoparticles when compared to Ag nanoparticles [40], a combination of Ag/ ZnO nanoparticles was used in this study for coating of orthodontic brackets and its antibacterial activity was compared to the antimicrobial effect of Ag and ZnO nanoparticles coatings individually.

The null hypothesis was that the antibacterial effects of the three types of coatings on the orthodontic stainless-steel brackets; Ag, ZnO and the combination of Ag/ ZnO nanoparticles were not to be significantly different.

## Methods

This study aimed at assessing the antibacterial effect of three types of nanoparticles; Ag, ZnO and a combination of both Ag and ZnO (Ag/ZnO) when applied as coatings on orthodontic stainless-steel brackets through physical vapor deposition, on two different strains of bacteria; *Streptococcus mutans* and *Lactobacillus acidophilus*. This evaluation was to be carried out immediately after coating (T1) and after 3 months (T2) to see if the antibacterial effect, if present, persisted.

The study was carried out at Faculty of Dentistry, Alexandria University, the Egyptian Nanotechnology center, Cairo University, El-Sheikh Zayed Campus and Faculty of Science, Cairo University.

### Sample grouping and preparation

The sample size was estimated based on assumptions of alpha error to be equal 5% and study power 80% [37, 43, 44], a total of 48 brackets were to be included.

The brackets were divided into four groups, each constituting 12 brackets: control group (brackets as received without modifications), Ag nanoparticles coated group, ZnO nanoparticles coated group and Ag/ ZnO nanoparticles coated group.

The brackets used were stainless steel “American orthodontics” 0.018’’ slot size brackets of lower premolars. Before coating the brackets, ultrasonication was done at Faculty of Dentistry, Alexandria University to remove any adventitious macroscopic contamination [37]. Prior to storage in an airtight container, the brackets were thoroughly cleaned and sterilized using an autoclave.

### Coating procedure

**Fig. 1:**
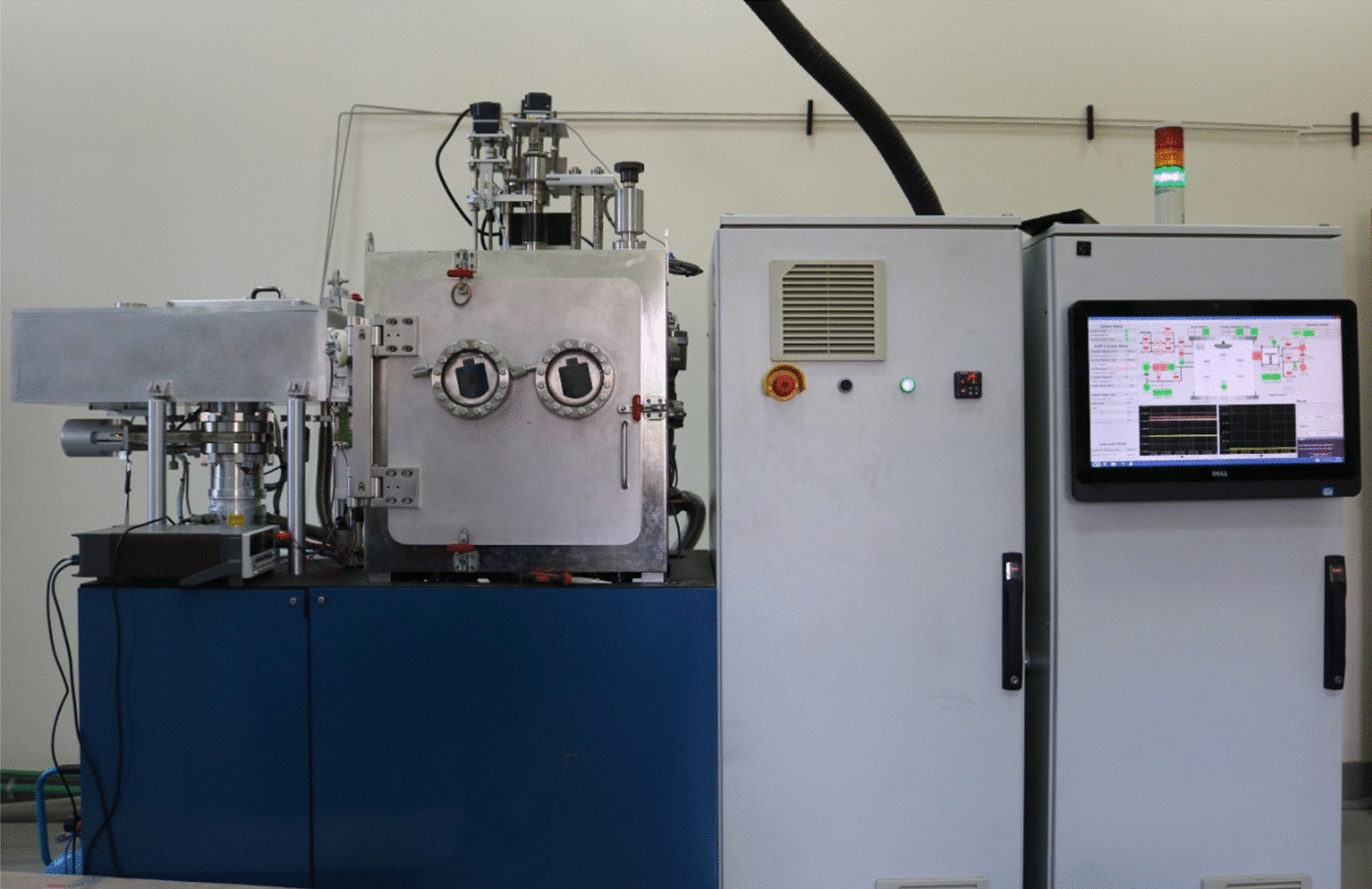
The Physical Vapor deposition (PVD) machine

**Fig. 2:**
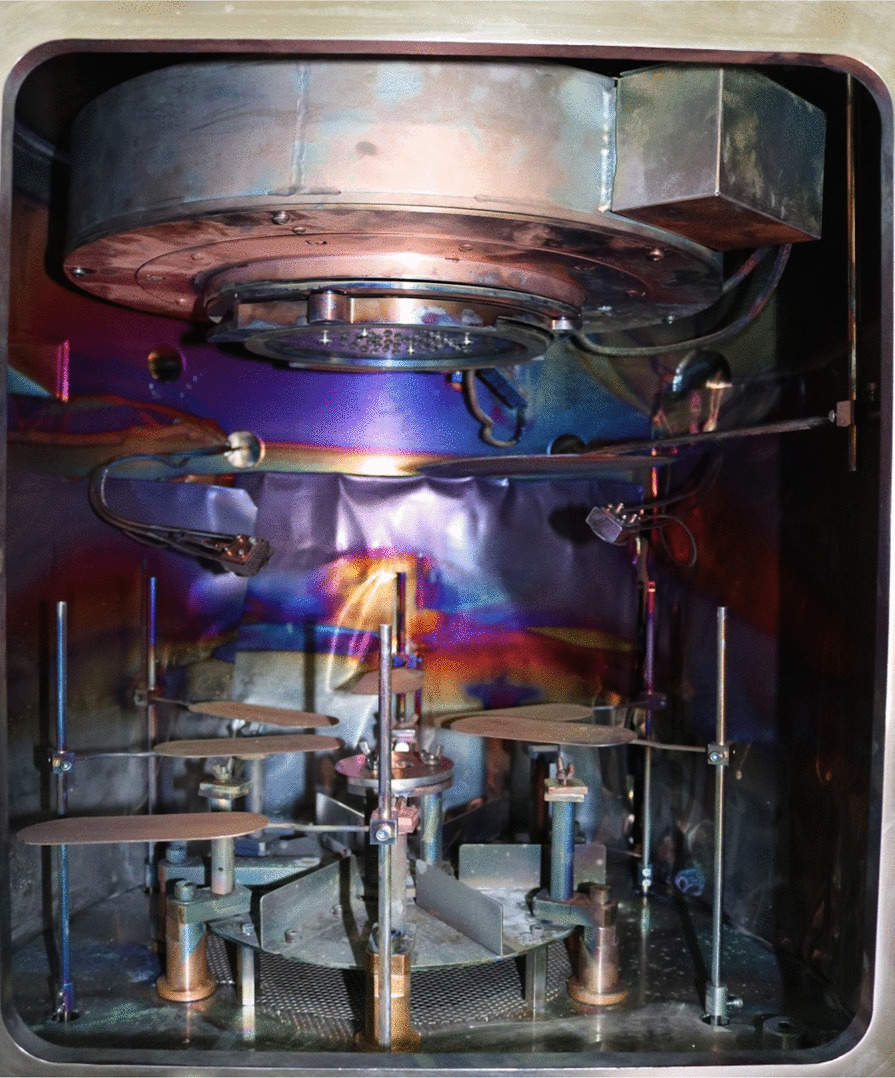
The Evaporation Chamber

Physical vapor deposition was carried out using PROTOFLEX 1400 machine (USA; Figs. 1 and 2) at the Egyptian Nanotechnology center, Cairo University, El-Sheikh Zayed Campus. Thermal Evaporation was used in which Ag and/ or ZnO were vaporized followed by their deposition and coating of the surface of the orthodontic brackets. First vacuum environment was achieved through evacuation of the chamber and reaching pressure of 10^–6^ Torr* to avoid any gaseous contamination during the deposition process. Then heating the tungsten boats on which the Ag and/or ZnO were placed started. After reaching the sublimation temperature, which is the temperature at which solid material is converted into gas or vapor, for Ag and/or ZnO, the shutter was opened to allow the vapor to reach the brackets [45]. After reaching a deposition of 50 nm thickness according to the detector present in the chamber, the process was stopped.

### Characterization

**Fig. 3:**
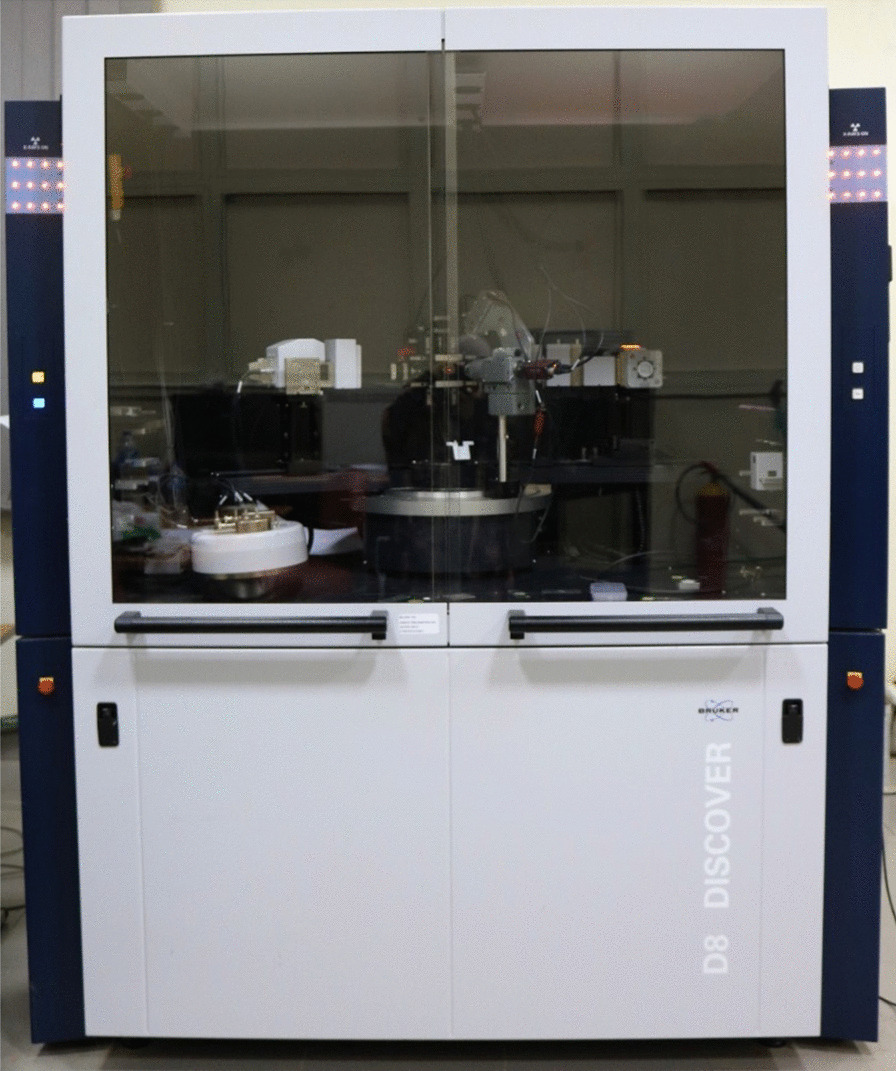
The X-ray Diffraction (XRD) machine

Characterization for the coated brackets was carried out using Atomic Force Microscope (AFM) of Agilent technologies (California, United States) and X-ray Diffraction (XRD) using Bruker, model D8 Discover (Billerica, Massachusetts, United States) (Fig. 3) at the Egyptian Nanotechnology center, Cairo University, El-Sheikh Zayed Campus.

### Antibacterial activity assessment

#### Streptococcus mutans

*S. mutans* strain (ATCC 25,175) was prepared using a suspension of concentration 1.5 × 10^6^ CFU/mL. 3 mL of liquid medium was added to 100 μL of the suspension in test tubes. Brackets of the four groups were then added to the test tubes. Under visible light, these tubes were incubated at 37 °C for 60 min. 10 mL of sterile saline was used for diluting 10 μL of the suspension, then this dilution was plated onto nutrient agar plates at a volume of 10 μL and cultivated for 24 h at 37 °C. The antibacterial activity in our study was described in terms of CFU [46] and percent of inhibition, which was calculated according to the equation:

$$\left( {{\text{CFU}}\;{\text{of}}\;{\text{Control}} - {\text{CFU}}\;{\text{of}}\;{\text{Drug}}} \right)/{\text{CFU}}\;{\text{of}}\;{\text{Control}}*{1}00.$$

#### Lactobacillus acidophilus

The process of incubation and evaluating the antibacterial effect of the brackets was carried out in the same manner as *S. mutans* [46]*.*

After the first antibacterial test, carried out immediately after coating, the brackets were washed using phosphate-buffered saline and sterilized under Ultraviolet lamp (UV lamp) to remove any hanging bacteria**.** They were then preserved for 3 months at 37 °C in artificial saliva and replenished weekly to mimic the oral environment. Then the antibacterial test was performed in the same manner as the first test.

### Statistical analysis

The data were analyzed using the software program Minitab®19. Descriptive statistical analysis of data was done for colony counting for the four groups at both times intervals, after coating and 3 months later. The data were represented as mean ± standard deviation. The One-way ANOVA along with Post Hoc test were used to compare the effects between the groups over the two periods of time. *P*-value < 0.05 was considered statistically significant.

## Results

### Characterization

#### AFM

**Fig. 4:**
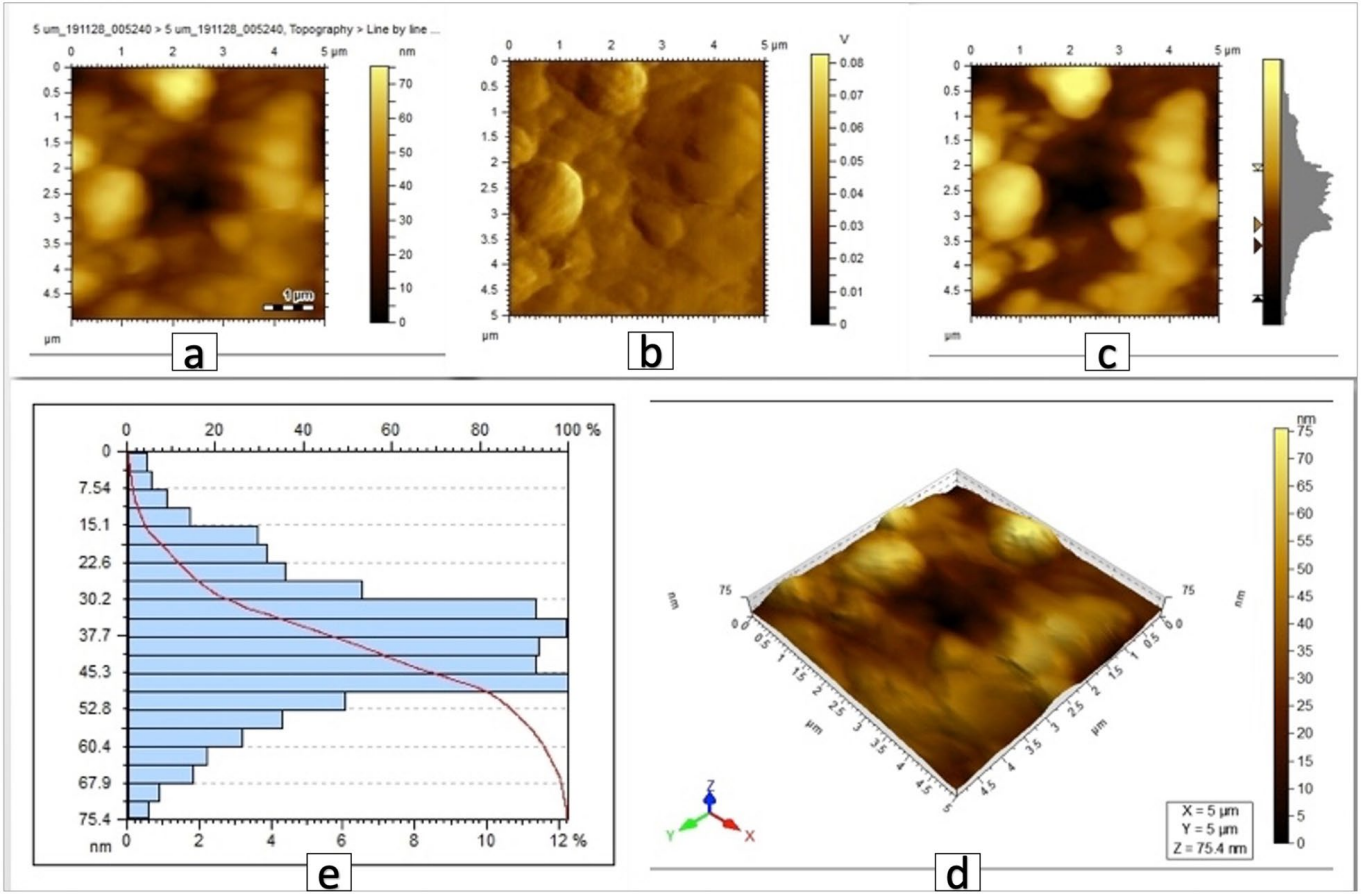
AFM images of brackets coated with Ag nanoparticles, scanned area 5 µm × 5 µm. **a**, **b**, **c** 2D images showing the Ag particles, **d** 3D view showing the peak height of the coating to be 75.4 nm, **e** histogram reflecting the size distribution (diameter in nm vs.% of particles) showing the average of particles size to be 38.68 nm

**Fig. 5:**
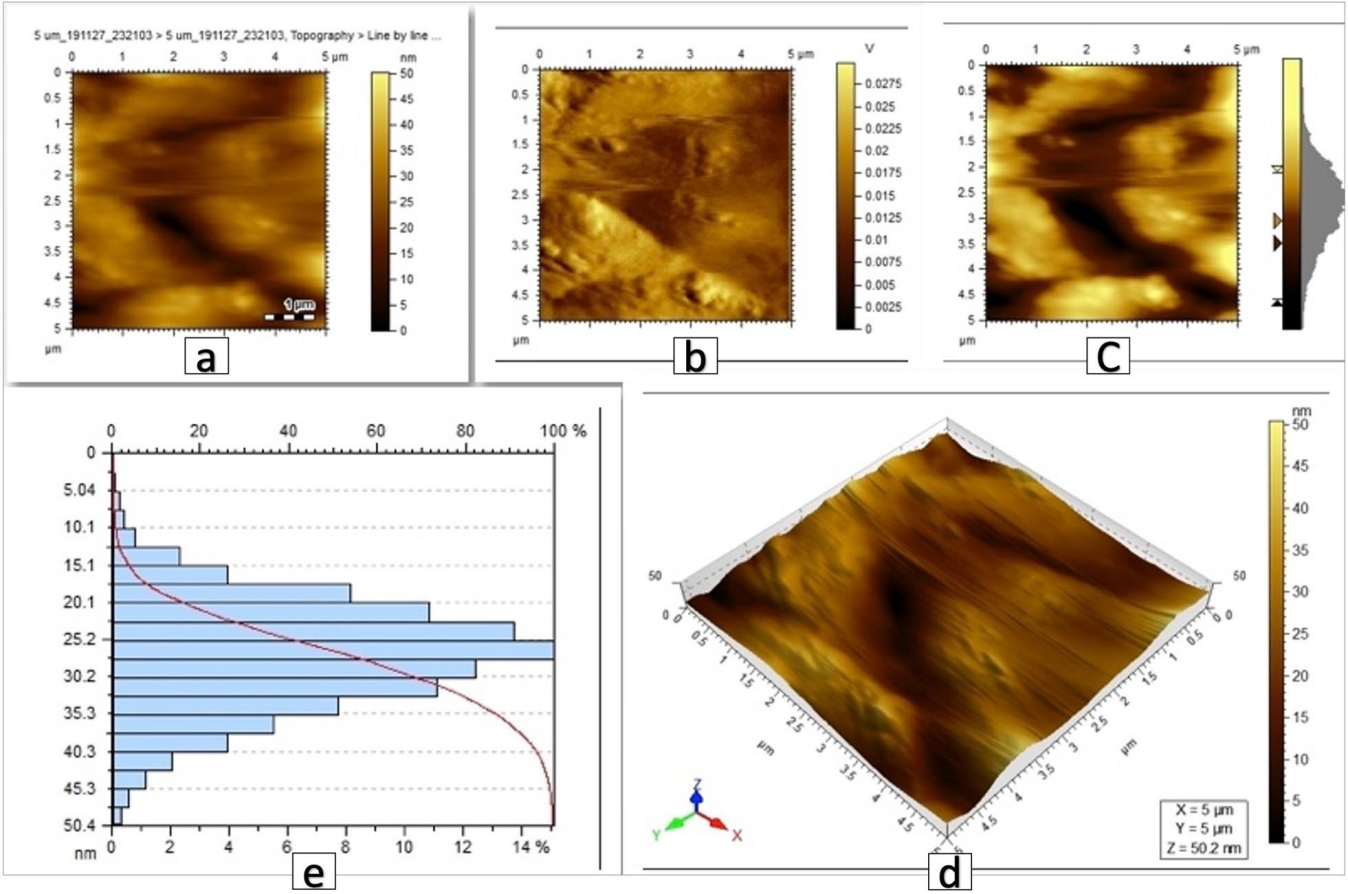
AFM images of brackets coated with ZnO nanoparticles, scanned area 5 µm × 5 µm. **a**, **b**, **c** 2D images showing the ZnO particles, **d** 3D view showing the peak height of the coating to be 50.2 nm, **e** histogram reflecting the size distribution (diameter in nm vs.% of particles) showing the average of particles size to be 27.7 nm

**Fig. 6:**
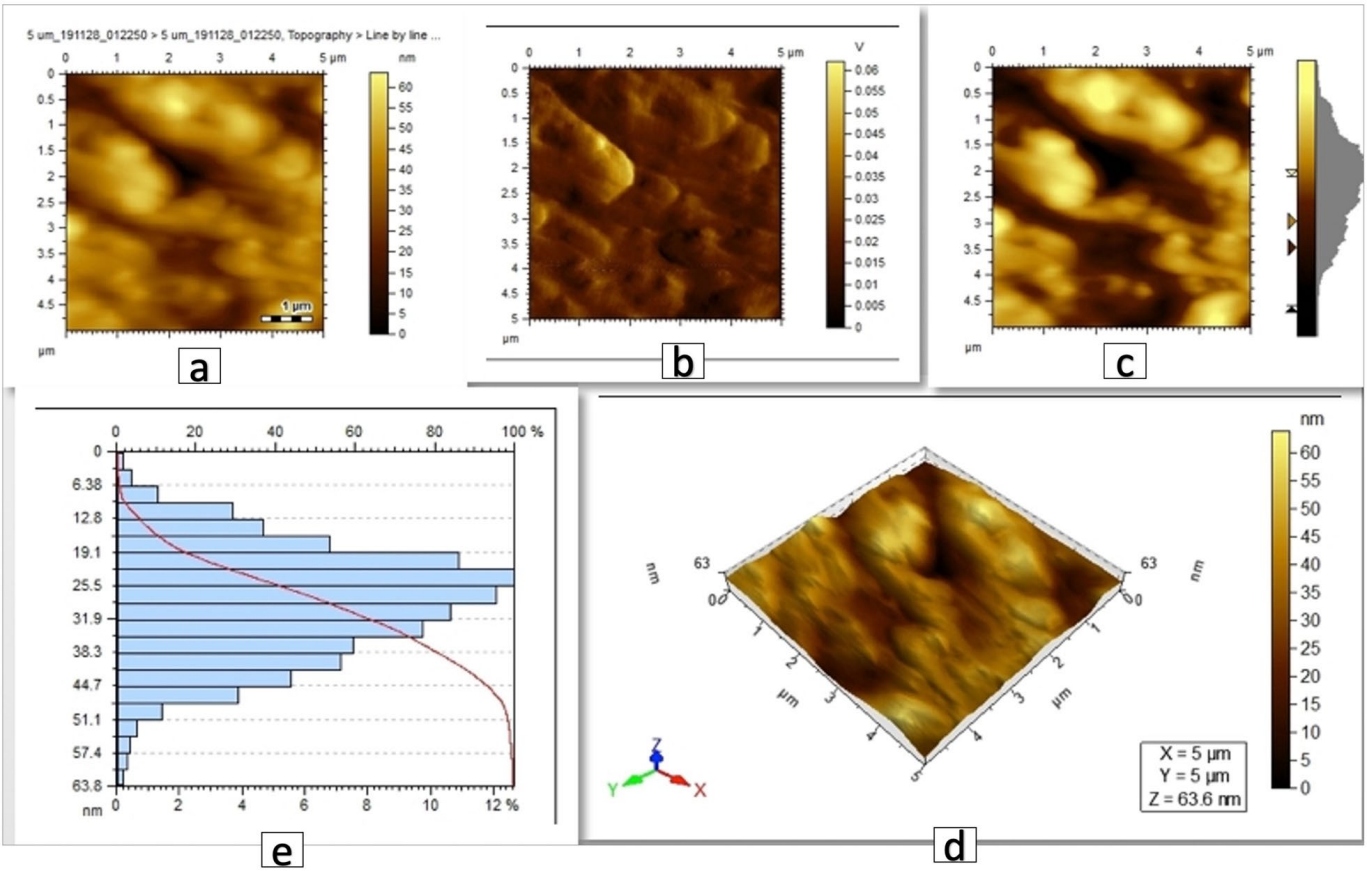
AFM images of brackets coated with Ag/ZnO nanoparticles, scanned area 5 µm × 5 µm. **a**, **b**, **c** 2D images showing the Ag/ZnO particles, **d** 3D view showing the peak height of the coating to be 63.6 nm, **e** histogram reflecting the size distribution (diameter in nm vs.% of particles) showing the average of particles size to be 26.8 nm

AFM pseudo-color plots for the coated brackets showed the surface difference between the three coated groups (Figs. 4, 5, 6).

#### XRD

XRD results reflected the presence of Ag, ZnO and Ag/ZnO according to the shown peak intensities (Two Theta (2θ)), accordingly the obtained d-spacings confirmed the coatings material. At 2θ = 40.3, d-spacing was = 2.3579, which reflects Ag nanoparticles in the Ag coated brackets. at 2θ = 33.4, d-spacing was = 2.68010 which reflects the presence of ZnO coating. For the Ag/ZnO nanoparticles coated brackets, at 2θ = 38.829, d-spacing was = 2.31740, and also at 2θ = 33.4, d-spacing was = 2.68010, as both were found, the presence of both Ag and ZnO in the coating was confirmed.

### Antibacterial activity

## Antibacterial effect of brackets coating on *S. mutans* immediately after coating (T1)

**Fig. 7:**
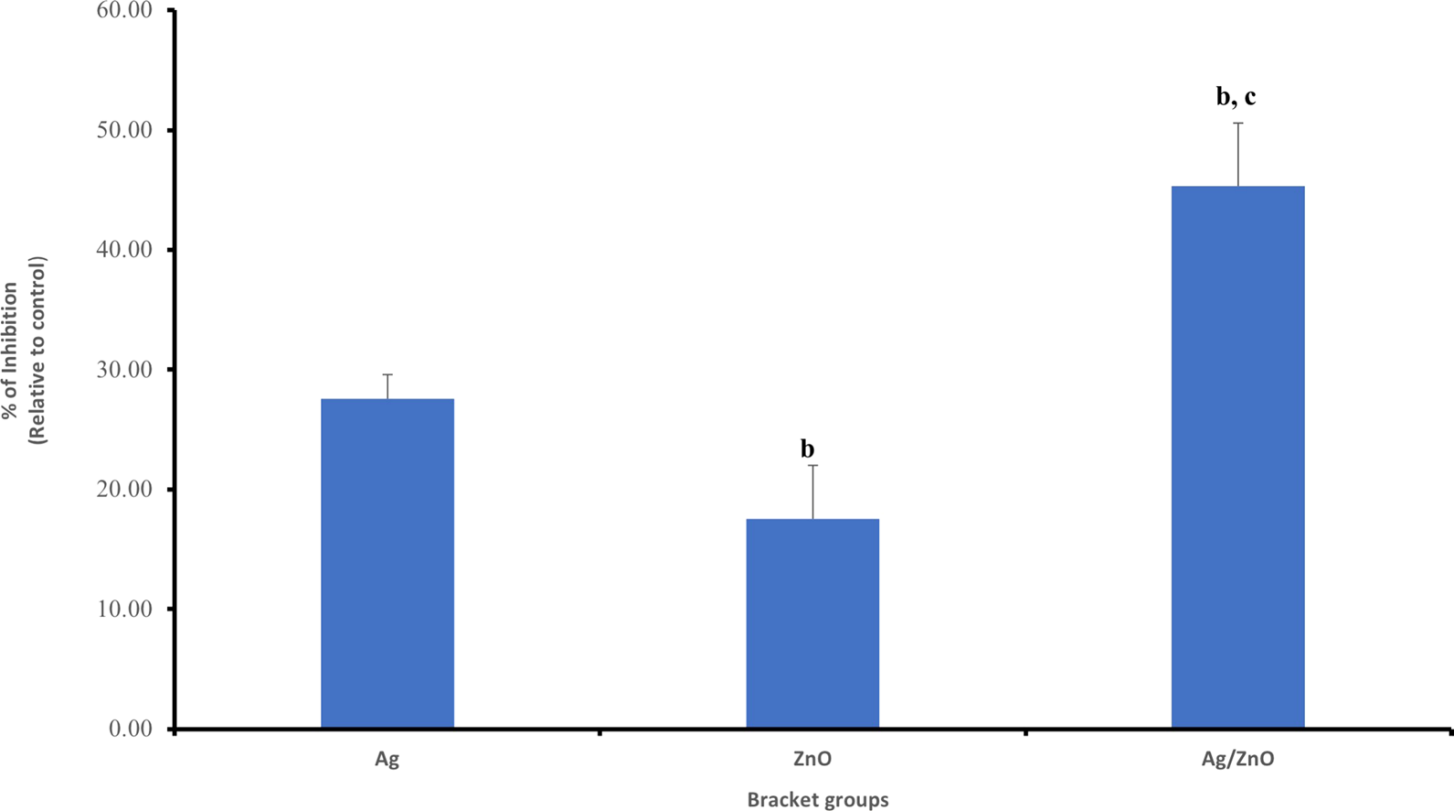
Showing percent of inhibition of the three experimental groups on *S. mutans* at T1, ^b^*P*-value < 0.05 versus Ag-group, ^C^*P*-value < 0.05 versus ZnO-group

Rate of survival of the *S. mutans* bacterial cells in control group was 1403.75 ± 4.20% CFU. In Ag coated group, the survival rate was 1016.25 ± 2.80% CFU. The survival rate in the ZnO coated group was, 1157.50 ± 5.40% CFU. While in the Ag/ ZnO coated group, the survival rate was 767.50 ± 9.60% CFU (as demonstrated in Table 1, and as provided in the additional file 1 for statistical analysis). *P*-value for the 3 experimental groups was < 0.05. It was found that the percent of inhibition of Ag is 27.60% ± 2.00%, ZnO is 17.54% ± 4.49% and Ag/ZnO is 45.32% ± 5.26%. Therefore, in comparison to the control group, the three experimental groups showed statistically significant difference in reduction of survival rate of *S. mutans*. However, the Ag/ ZnO was the most effective, followed by the Ag group and then the ZnO coated groups (Fig. 7).

**Table 1:** Group statistics for colony count of *S. mutans* at two-time intervals

Brackets groups	Colony count of *S. mutans*	
	T1	T2
Control	1403.75 ± 4.20	1326.00 ± 5.05
Ag	1016.25 ± 2.80 ^a^	1006.66 ± 9.08 ^a^
ZnO	1157.50 ± 5.40 ^a, b^	1122.00 ± 8.89 ^a, b^
Ag/ZnO	767.50 ± 9.60 ^a, b, c^	752.00 ± 9.38 ^a, b, c^

T1: test after coating, T2: after 3 months

Data expressed as mean ± S.D%. (n = 12). Data were analyzed using ANOVA followed by Post Hoc pairwise comparisons. Statistically significant at ^a^*P*-value < 0.05 vs control, ^b^*P*-value < 0.05 versus Ag-group, ^C^*P*-value < 0.05 versus ZnO-group

**Fig. 8:**
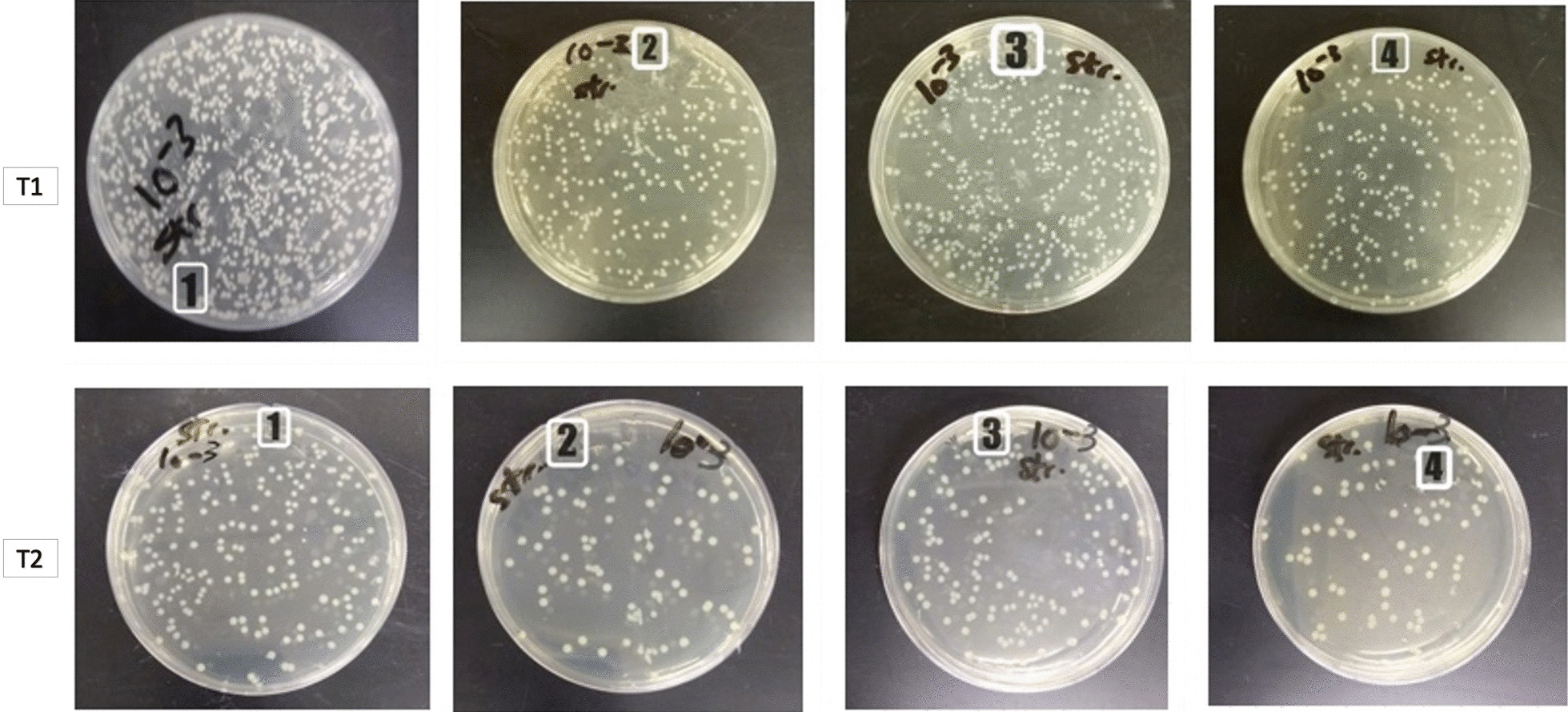
Petri dishes of *S. mutans* of the 4 groups, T1: immediately after coating and T2: post 3 months test (1 = control group, 2 = Ag coated group, 3 = ZnO coated group and 4 = Ag/ZnO coated group)

The Petri dishes showing the effect of the control and experimental groups on *S. mutans* at T1 are shown in Fig. 8.

## Antibacterial effect of brackets coating on *S. mutans* after 3 months (T2)

**Fig. 9:**
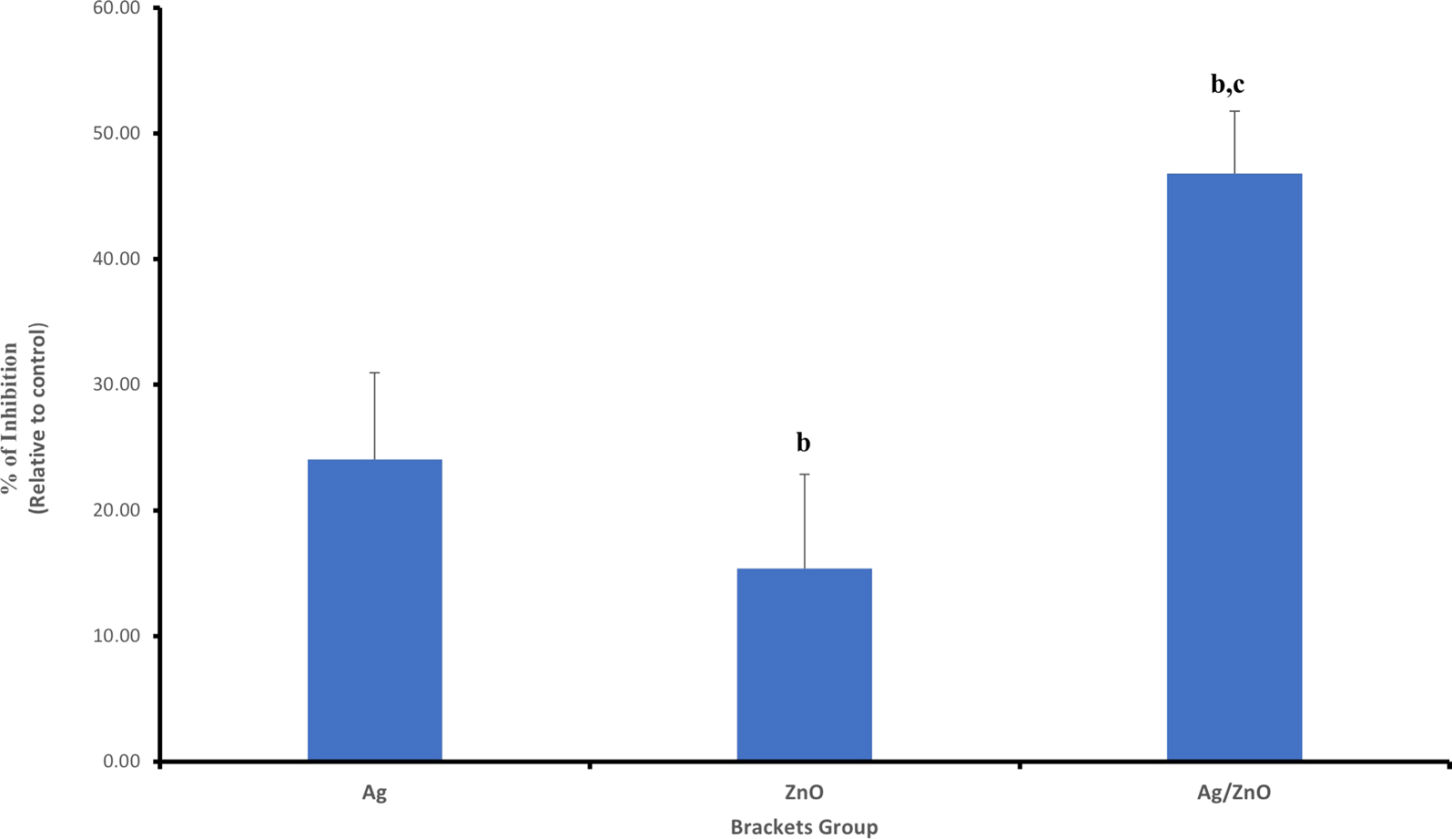
Showing percent of inhibition of the three experimental groups on *S. mutans* at T2, ^b^*P*-value < 0.05 versus Ag-group, ^C^*P*-value < 0.05 versus ZnO-group

The survival rate of bacteria in control group was 1326.00 ± 5.05% CFU. For the Ag coated brackets, the survival rate was 1006.66 ± 9.08% CFU. The effect of ZnO coating on survival rate of the bacterial cells was 1122.00 ± 8.89% CFU. And finally, for the Ag/ ZnO oxide group, the survival rate was 752.00 ± 9.38% CFU (as shown in Table 1, and as provided in the additional file 2 for statistical analysis). The *P*-value for the 3 experimental groups was < 0.05. The percent of inhibition of the Ag, ZnO and Ag/ZnO on the bacteria was 24.08% ± 6.89%, 15.38% ± 7.52% and 43.29% ± 5.32%, respectively. These results indicated that the three experimental groups showed statistically significant reduction on bacterial growth, with Ag/ ZnO group having the highest effect, followed by Ag coating and then ZnO (Fig. 9).

The Petri dishes showing the effect of the control and experimental groups on *S. mutans* at T2 (test carried out after 3 months) are shown in Fig. 8.

## Antibacterial effect of brackets coating on *L. acidophilus* immediately after coating (T1)

**Fig. 10:**
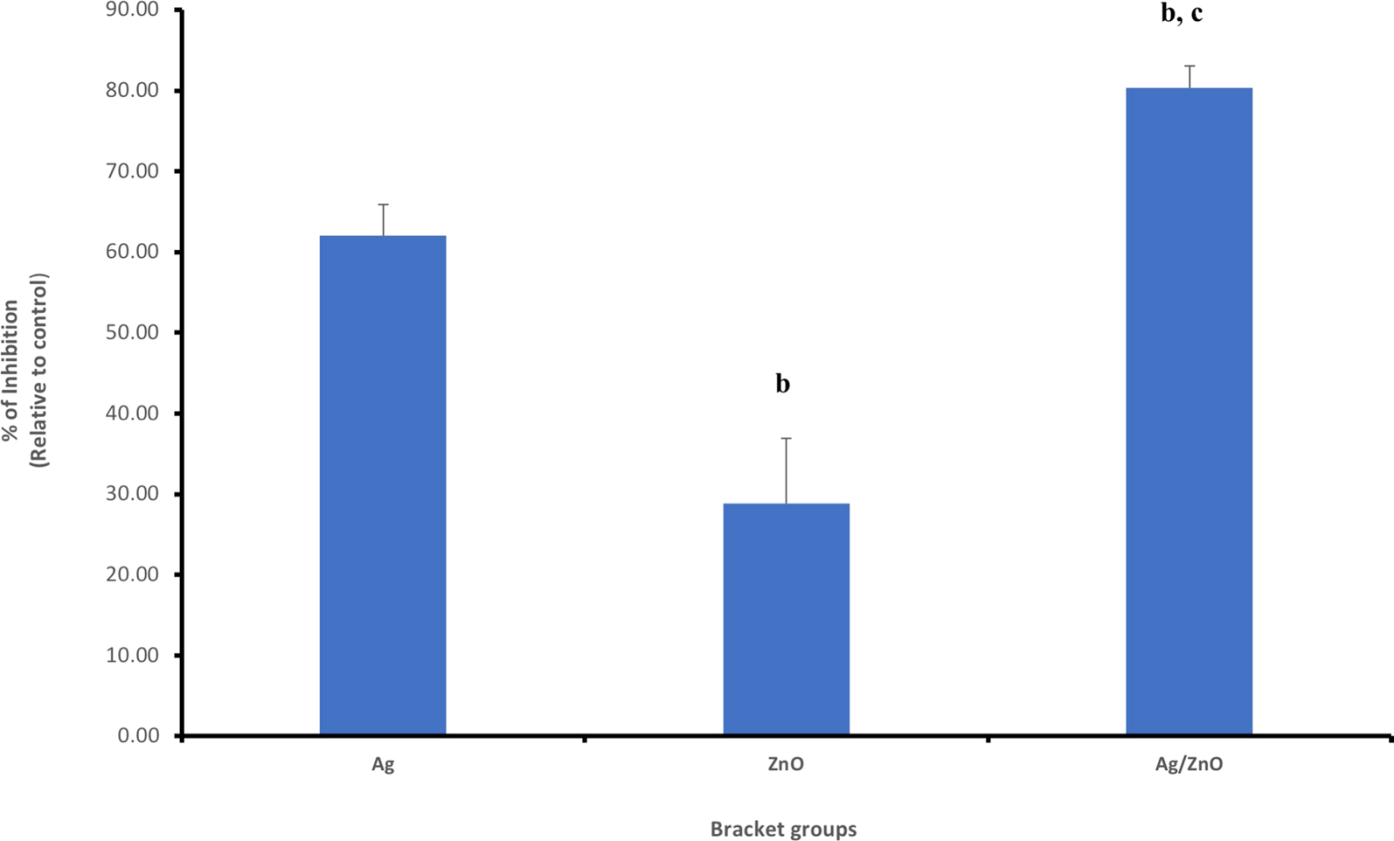
Showing percent of inhibition of the three experimental groups on *L. acidophilus* at T1, ^b^*P*-value < 0.05 versus Ag-group, ^C^*P*-value < 0.05 versus ZnO-group

The control group showed survival rate of 65 ± 11.40% CFU on *L. acidophilus*. The Ag coated brackets showed survival rate of 24.69 ± 13.68% CFU. For the ZnO group, the survival rate was found to be 46.25 ± 12.62% CFU and finally for the Ag/ZnO group, the survival rate was 12.81 ± 15.07% CFU (demonstrated in Table 2, and as provided in additional file 3 for statistical analysis). *P*-value was < 0.05. The percent of inhibition of Ag was 62.02%, for ZnO was 28.85% and for Ag/ZnO was 80.29%. The results of the three groups were statistically different when compared to the control group, with Ag/ZnO coated brackets have the highest effect on reducing the bacterial counts, followed by Ag and then ZnO (Fig. 10).

**Table 2:** Group statistics for colony count of *L. acidophilus* at two-time intervals

Brackets groups	Colony Count of *L. acidophilus*	
	T1	T2
Control	65.00 ± 11.40	71.25 ± 11.22
Ag	24.69 ± 13.68^a^	26.25 ± 10.55^a^
ZnO	46.25 ± 12.60^a, b^	48.75 ± 11.83 ^a, b^
Ag/ZnO	12.81 ± 15.07^a, b, c^	13.13 ± 14.92 ^a, b, c^

T1: test after coating, T2: after 3 months

Data expressed as mean ± S.D%. (n = 12). Data were analysed using ANOVA followed by Post Hoc pairwise comparisons. Statistically significant at ^a^*P*-value < 0.05 versus control, ^b^*P*-value < 0.05 versus Ag-group, ^C^*P*-value < 0.05 versus ZnO-group

**Fig. 11:**
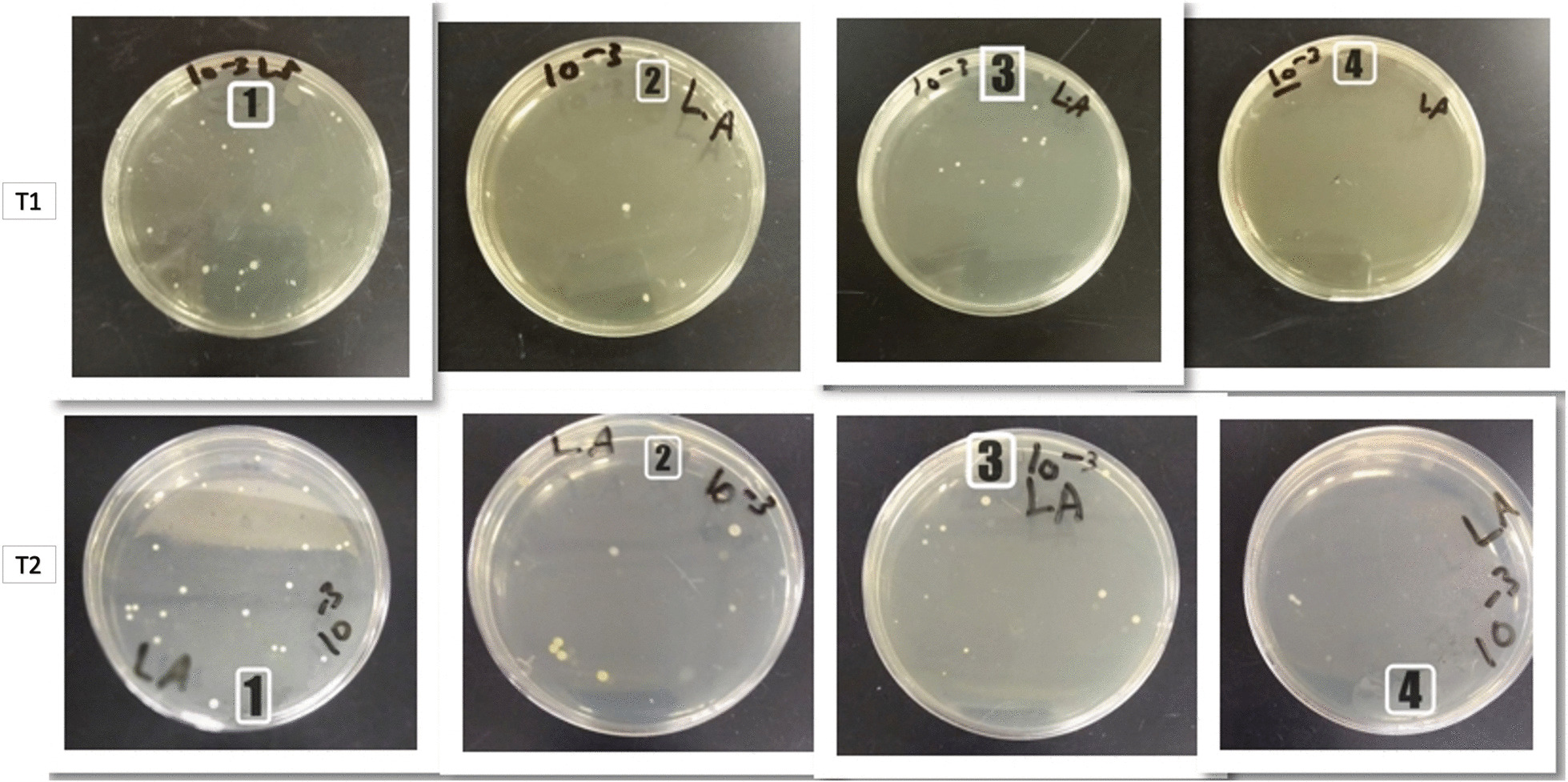
Petri dishes of *L. acidophilus* of the 4 groups, T1: immediately after coating and T2: post 3 months test (1 = control group, 2 = Ag coated group, 3 = ZnO coated group and 4 = Ag/ZnO coated group)

The Petri dishes showing the effect of the control and experimental groups on *L. acidophilus* at T1 are shown in Fig. 11.

## Antibacterial effect of brackets coating on *L. acidophilus* after 3 months (T2)

**Fig. 12:**
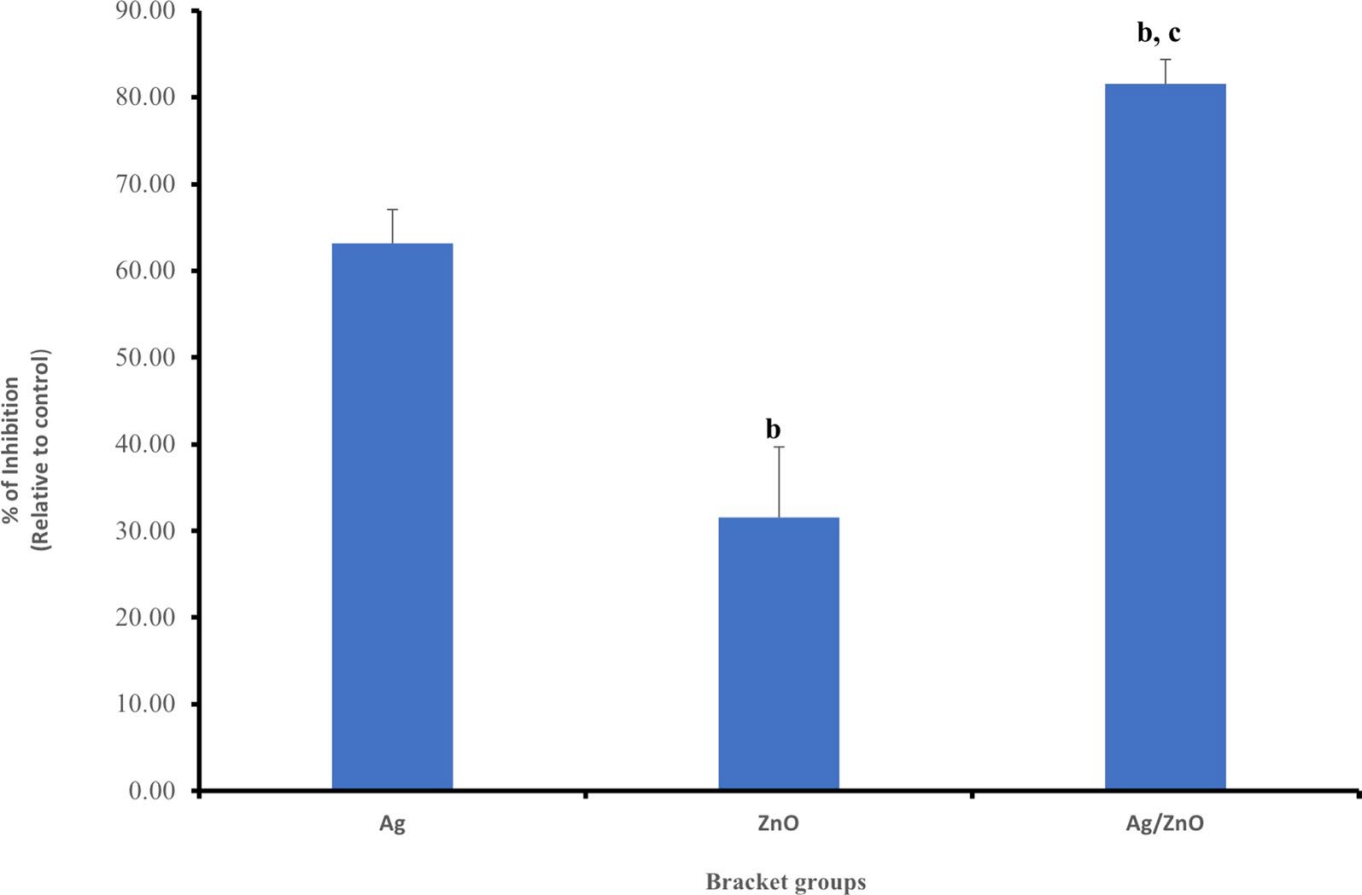
Showing percent of inhibition of the three experimental groups on *L. acidophilus* at T2, ^b^*P*-value < 0.05 versus Ag-group, ^C^*P*-value < 0.05 versus ZnO-group

The survival rate of *L. acidophilus* under the effect of control group after 3 months from the initial test was found to be 71.25 ± 11.22% CFU. The effect of Ag coating was 26.25 ± 10.55% CFU. For the ZnO, it was found to be 48.75 ± 11.80% CFU. And for the Ag/ZnO group, was found to be 13.13 ± 14.92% CFU (shown in Table 2, and as shown in additional file 4 for statistical analysis). The *P*-value was < 0.05. As for the percent of inhibition, it was found to be 63.16%, 31.58% and 81.58% for the Ag, ZnO and Ag/ZnO groups respectively. The results were statistically significant on reducing the *L. acidophilus* survival rate, with Ag/ZnO having the highest effect, followed by Ag and finally ZnO (Fig. 12).

The Petri dishes showing the effect of the control and experimental groups on *L. acidophilus* at T2 are shown in Fig. 11.

## Antibacterial effect at the two-time intervals (T1 vs T2) for the experimental coated brackets

The *P*-value was calculated between the results of the antibacterial tests carried out at T1 and T2 for the three experimental groups on both bacterial strains: *S. mutans* and *L. acidophilus*, to see if the effect of the coating changed over time significantly or not. The test was carried out using Student t test, with *P*-value < 0.05 was to be considered significantly different.

On *S. mutans*, the *P*-value between the mean of CFU was 0.744, 0.415 and 0.332 for the Ag, ZnO and Ag/ZnO coated brackets respectively, indicating no significant difference between the two-times intervals (as presented in the additional files 5, 6, 7, 8, 9 and 10 for statistical analysis).

On *L. acidophilus*, the *P*-value between the mean of CFU was 0.175, 0.339 and 0.674 for the Ag, ZnO and Ag/ZnO coated brackets respectively, indicating no significant difference between the two-times intervals (as presented in additional files 11, 12, 13, 14, 15 and 16 for statistical analysis).

## Discussion

This study aimed at evaluating the antibacterial effect of different surface modifications of stainless-steel orthodontic brackets through coating their surface with nano particles of silver, zinc oxide and combination of silver and zinc oxide. Possessing an antibacterial activity is an additional property that can help in overcoming one of the possible side effects of orthodontics especially in patients who don’t brush their teeth consistently [3, 4]. Leading to plaque accumulation, demineralization of teeth surface and white spot lesions formation [5, 47].

The null hypothesis presented, that no difference between the antibacterial effects of the three types of coatings on the orthodontic stainless-steel brackets, was rejected.

The results showed that brackets with surface coatings of Ag nanoparticles, ZnO nanoparticles and combination of Ag/ZnO nanoparticles showed significant difference in antibacterial effect against both *S. mutans* and *L. acidophilus* when compared to the control group. However, the Ag/ZnO nanoparticle group showed the highest antibacterial effect, followed by the Ag nanoparticle and then the ZnO nanoparticle groups.

Our results agreed with Nalini et al. [48] who found enhanced antibacterial effect for nano Ag coated brackets on *S. mutans* and *L. acidophilus*. Also, the results agreed with the findings of Hernández-Sierra et al. [49] in which the antimicrobial activity of nanoparticles of Ag, ZnO and gold (Au) on *S. mutans* were investigated, and the results presented higher antimicrobial activity of Ag nanoparticles than ZnO and Au nanoparticles. It also demonstrated higher antimicrobial activity for Ag nanoparticles at lower concentration than ZnO and Au nanoparticles. Which may suggest that the decreased effect of ZnO nanoparticles in our study might be due to insufficient concentration for the ZnO to express pleasant antibacterial effect. This also agreed with the findings of Yamamto et al. [50] who stated that the antibacterial activity for ZnO was directly related to its concentration.

Silver nanoparticles are characterized by its synergistic effect when combined with other natural or synthetic compounds [51]. As previously described by Cardozo et al. [52] on combining Ag nanoparticles with phenazine-1-carboxamide, lead to an increase in the antibacterial effect against *Staphylococcus aureus* through morphological alterations of the bacterial cell wall. As also revealed by Biasi-Garbin et al*.* [53], Ag nanoparticles showed strong synergistic effect when combined with eugenol significantly reducing the minimum concentrations needed from both compounds to inhibit the bacterial growth of *Streptococcus agalactiae*. Comparably, this is consistent with the results of our study where the combination of Ag and ZnO nanoparticles resulted in an increased antibacterial effect on both *S. mutans* and *L. acidophilus* although the concentration of Ag nanoparticles in this group was less than that of the group coated with Ag nanoparticles only. Where the coat thickness was standardized of average 50 nm for all groups, meaning that the percentage of Ag in the combination group is less than forming the whole thickness in Ag only coated group. These results agree with the findings of the study carried out to assess the antibacterial activity and mechanism of Ag/ZnO nanocomposite against *S. mutans* [25]*,* which displayed enhanced antibacterial effect against *S. mutans* in comparison to ZnO nanorods. This was explained as a result of damaging the cell structure and membrane, in addition to the formation of ROS through assessing malondialdehyde (MDA) which is an oxidized product of polyunsaturated fatty acid. This is indicative for the oxidative damage caused by Ag/ZnO. Additionally, a bioactive composite resin containing Ag/ZnO nanoparticles was developed by Kachoei et al*.* [54] as antimicrobial material. Its effect was measured on *S. mutans*, *L. acidophilus* and *Candida albicans*. The study compared the effect of ZnO nanoparticles, ZnO nanoparticles and Ag ions and Ag/ZnO nanoparticles. The three groups showed statistically significant results as antimicrobial agents with nanocomposite containing Ag/ZnO nanoparticles possessing the highest antimicrobial activity.

In our study the coating was of average thickness 50 nm, for the aim of standardization and avoiding cytotoxicity when applied in further in vivo studies. In literature, few studies using the PVD technique have mentioned the performed film thickness. Ghasemi [37], who used two thicknesses of 60 nm and 100 nm of Ag and TiO_2_ nanoparticles, stated that there was no difference between the two thicknesses as antibacterial effect on *S. mutans*. Also, an in vivo study conducted on rats [55], mandibular incisor brackets coated with 1 μm of Ag nanoparticles were found to be effective as antibacterial agent against *S. mutans* and regarding its cytotoxicity were considered safe, as the serum concentration of Ag ions was found to be 0.00175 μg/L. Accordingly, coating of 50 nm thickness was chosen for each bracket, as in patients 20 brackets will be used (50 nm × 20 = 1000 nm = 1 μm) and supposedly, they cause the same cumulative level of nano Ag in saliva which was considered safe.

The synthesis of combination of Ag and ZnO was for the purpose of reducing any possible risk for Ag nanoparticles to be cytotoxic and genotoxic on human cells [41, 42], and obtaining the benefits of its enhanced antibacterial activity over ZnO [40].

The experimental groups in our study were re-assessed after 3 months from the initial antibacterial test to check if the antibacterial effect of the coatings persisted. However, the effect of friction, that could result from several abrasive actions in the patients’ mouth, on the coating layer was not evaluated. As the main aim was testing the antibacterial activity and its persistence over time. Few studies have experimented the long-term antibacterial effect of the coated brackets. Of those studies, one was assessed after 30 days [56], and the other was carried out at three time intervals; 30 days, 60 days and 90 days [46], and both of them stated no statistically significant difference between the results of the tests carried out over time. Therefore, 3 months period was chosen for re-evaluation in our study. The results were that Ag/ZnO coated group had the highest effect on reduction of both *S. mutans* and *L. acidophilus*, followed by Ag then ZnO without significant difference concerning the antimicrobial effect of the three groups over time, indicating the persistence of the antimicrobial activity of the bracket coatings over time.

### Limitations of this study

1. Only 50 nm of film thickness was used for coating of the brackets.
2. The effect of friction on the nanoparticles coating layer was not tested in this study.

### Conclusion

- Ag/ZnO coated brackets had the highest antibacterial effect in comparison to Ag and ZnO individually coated brackets on *S. mutans* and *L. acidophilus*, and all types of coatings showed enhanced antibacterial effect in comparison to the uncoated bracket.
- Over a period of 3 months, the antibacterial effect of the 3 types of coatings persisted without significance difference compared to the effect after immediate coating.

Therefore, further studies are needed to evaluate the cytotoxic and other side effects for the brackets coated with nanoparticles of Ag/ZnO and its efficiency in reducing the incidence of WSLs clinically over the length of orthodontic treatment time. Also, to study the effect of abrasive actions and friction on the nanoparticles coating layer.

## Supplementary Information

**Additional file 1:** Comparison of the CFU of the four groups on *S. mutans* at T1.

**Additional file 2:** Comparison of the CFU of the four groups on *S. mutans* at T2.

**Additional file 3:** Comparison of the CFU of the four groups on *L. acidophilus* at T1.

**Additional file 4:** Comparison of the CFU of the four groups on *L. acidophilus* at T2.

**Additional file 5:** CFU at T1 vs T2 for Ag coated group on *S. mutans*.

**Additional file 6:** CFU at T1 vs T2 for ZnO coated group on *S. mutans*.

**Additional file 7:** CFU at T1 vs T2 for Ag/ ZnO coated group on *S. mutans*.

**Additional file 8:** Percent of inhibition at T1 vs T2 for Ag coated group on *S. mutans*.

**Additional file 9:** Percent of inhibition at T1 vs T2 for ZnO coated group on *S. mutans*.

**Additional file 10:** Percent of inhibition at T1 vs T2 for Ag/ ZnO coated group on *S. mutans*.

**Additional file 11:** CFU at T1 vs T2 for Ag coated group on *L. acidophilus*.

**Additional file 12:** CFU at T1 vs T2 for ZnO coated group on *L. acidophilus*.

**Additional file 13:** CFU at T1 vs T2 for Ag/ ZnO coated group on *L. acidophilus*.

**Additional file 14:** Percent of inhibition at T1 vs T2 for Ag coated group on *L. acidophilus*.

**Additional file 15:** Percent of inhibition at T1 vs T2 for ZnO coated group on *L. acidophilus*.

**Additional file 16:** Percent of inhibition at T1 vs T2 for Ag/ ZnO coated group on *L. acidophilus*.

## Data Availability

All data generated or analysed during this study are included in this published article in the form of tables and figures.

## Abbreviations

AFM: Atomic Force Microscope
Ag: Silver
CuO: Copper Oxide
L. acidophilus: Lactobacillus acidophilus
PVD: Physical Vapor Deposition
ROS: Reactive Oxygen Species
*S. mutans*: *Streptococcus mutans*
TiO_2_ NP: Titanium dioxide nanoparticles
UV: Ultraviolet
WSLs: White Spot lesions
XRD: X-ray Diffraction
ZnO: Zinc Oxide
