# Supplementary material for: The antibacterial effect of silver, zinc-oxide and combination of silver/ zinc oxide nanoparticles coating of orthodontic brackets (an in vitro study)
Source: BMC Oral Health. 2022 Jun 9;22:230. doi: 10.1186/s12903-022-02263-6 (PMC9185939; doi:10.1186/s12903-022-02263-6)
Supplement: Supplementary file 1 — Additional file 1: Comparison of the CFU of the four groups on S. mutans at T1. [file 12903_2022_2263_MOESM1_ESM.pdf]

Strepto_1

WORKSHEET 1

One-way ANOVA: Ag+Zno, zno, Ag, Control

Method

Null hypothesis	All means are equal
Alternative hypothesis	Not all means are equal
Significance level	α = 0.05

Equal variances were assumed for the analysis.

Factor Information

Factor	Levels	Values
Factor	4	Ag+Zno, zno, Ag, Control

Analysis of Variance

Source	DF	Adj SS	Adj MS	F-Value	P-Value
Factor	3	2.54861E+14	8.49537E+13	249.35	0.000
Error	44	1.49906E+13	3.40696E+11		
Total	47	2.69852E+14			

Model Summary

S	R-sq	R-sq(adj)	R-sq(pred)
583692	94.44%	94.07%	93.39%

Means

Factor	N	Mean	StDev	95% CI
Ag+Zno	12	7675000	738376	(7335416, 8014584)
zno	12	11575000	630814	(11235416, 11914584)
Ag	12	10162500	281079	(9822916, 10502084)
Control	12	14037500	583655	(13697916, 14377084)

Pooled StDev = 583692

Tukey Pairwise Comparisons

Grouping Information Using the Tukey Method and 95% Confidence

Factor	N	Mean	Grouping			
Control	12	14037500	A			
zno	12	11575000		B		
Ag	12	10162500			C	
Ag+Zno	12	7675000				D

Means that do not share a letter are significantly different.

Tukey Simultaneous Tests for Differences of Means

Difference of Levels	Difference of Means	SE of Difference	95% CI	T-Value	Adjusted P-Value
zno - Ag+Zno	3900000	238291	(3263080, 4536920)	16.37	0.000010
Ag - Ag+Zno	2487500	238291	(1850580, 3124420)	10.44	0.000010
Control - Ag+Zno	6362500	238291	(5725580, 6999420)	26.70	0.000010
Ag - zno	-1412500	238291	(-2049420, -775580)	-5.93	0.000012
Control - zno	2462500	238291	(1825580, 3099420)	10.33	0.000010
Control - Ag	3875000	238291	(3238080, 4511920)	16.26	0.000010

Individual confidence level = 98.95%

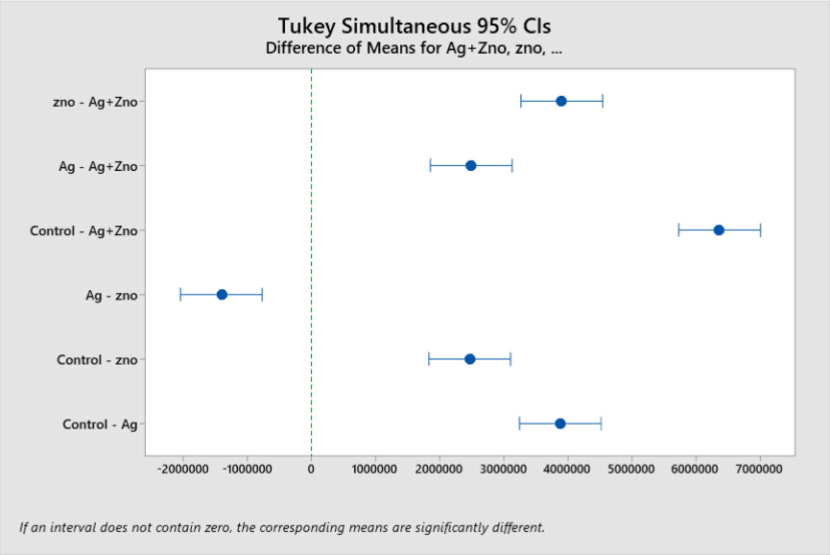

Dunnett Multiple Comparisons with a Control

Grouping Information Using the Dunnett Method and 95% Confidence

Factor	N	Mean	Grouping
Control (control)	12	14037500	A
zno	12	11575000	
Ag	12	10162500	
Ag+Zno	12	7675000	

Means not labeled with the letter A are significantly different from the control level mean.

Dunnett Simultaneous Tests for Level Mean - Control Mean

Difference of Levels	Difference of Means	SE of Difference	95% CI	T-Value	Adjusted P-Value
Ag+Zno - Control	-6362500	238291	(-6942242, -5782758)	-26.70	0.000
zno - Control	-2462500	238291	(-3042242, -1882758)	-10.33	0.000
Ag - Control	-3875000	238291	(-4454742, -3295258)	-16.26	0.000

Individual confidence level = 98.09%

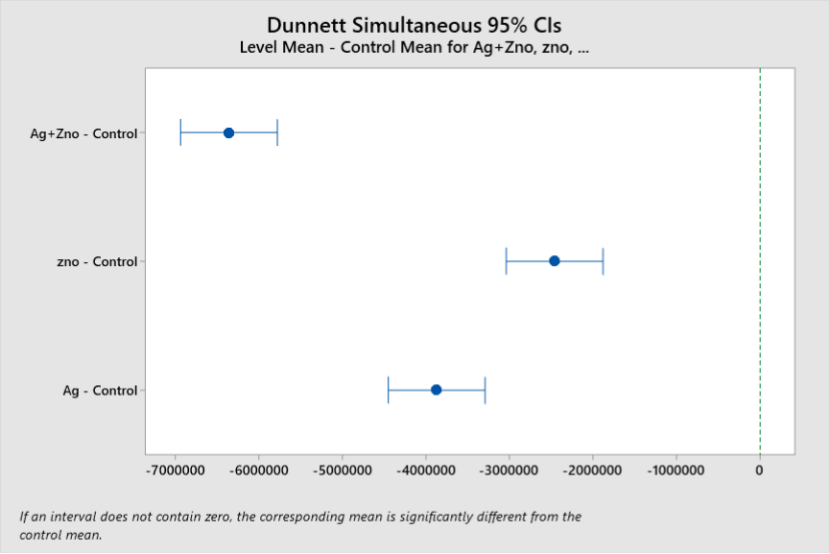

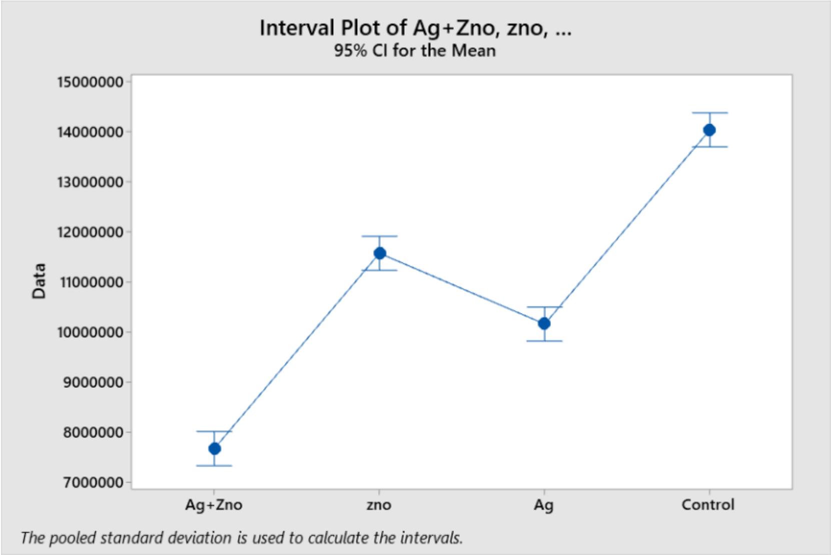
Interval Plot of Ag+Zno, zno, ...
95% CI for the Mean
Data
15000000
14000000
13000000
12000000
11000000
10000000
9000000
8000000
7000000
Ag+Zno
zno
Ag
Control
The pooled standard deviation is used to calculate the intervals.

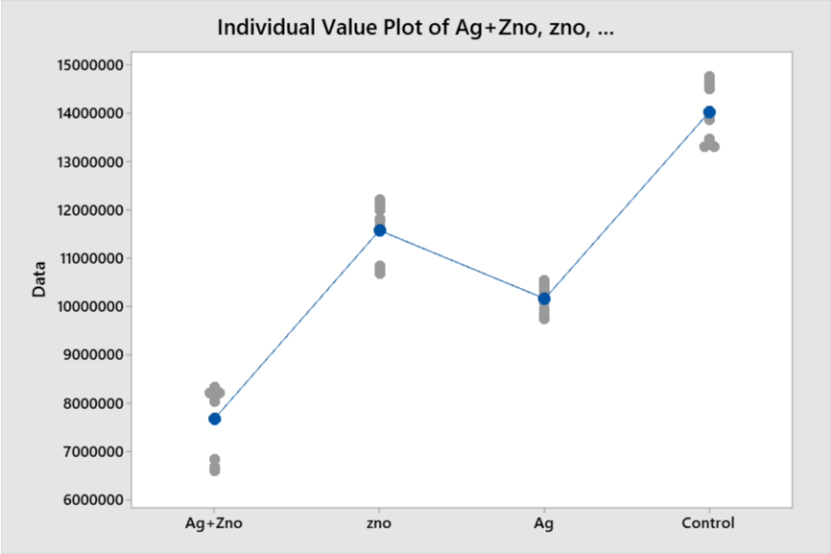
Individual Value Plot of Ag+Zno, zno, ...
Data
15000000
14000000
13000000
12000000
11000000
10000000
9000000
8000000
7000000
6000000
Ag+Zno
zno
Ag
Control

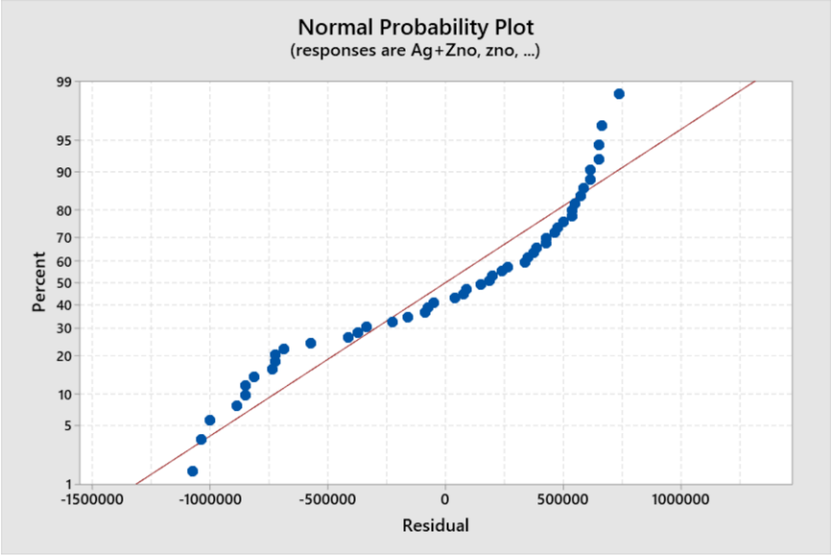
Normal Probability Plot
(responses are Ag+Zno, zno, ...)
Percent
99
95
90
80
70
60
50
40
30
20
10
5
1
-1500000
-1000000
-500000
0
500000
1000000
Residual

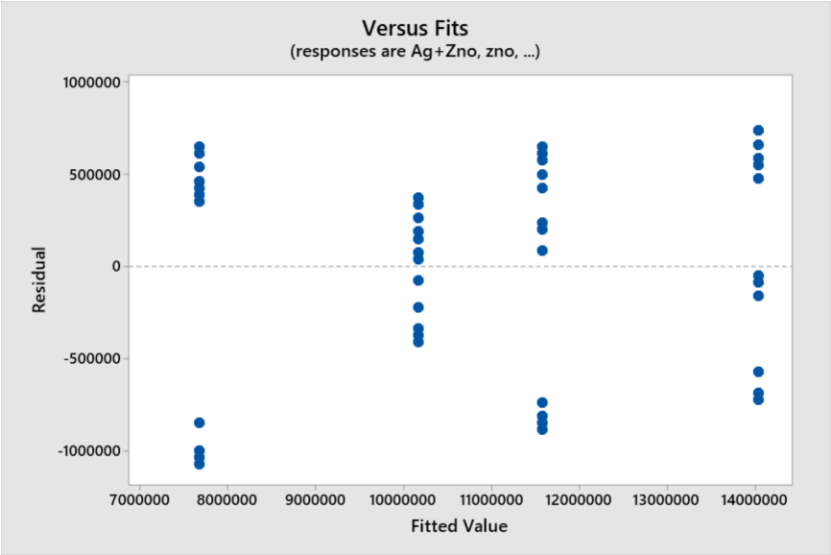
Versus Fits
(responses are Ag+Zno, zno, ...)
1000000
500000
0
-500000
-1000000
Residual
7000000
8000000
9000000
10000000
11000000
12000000
13000000
14000000
Fitted Value

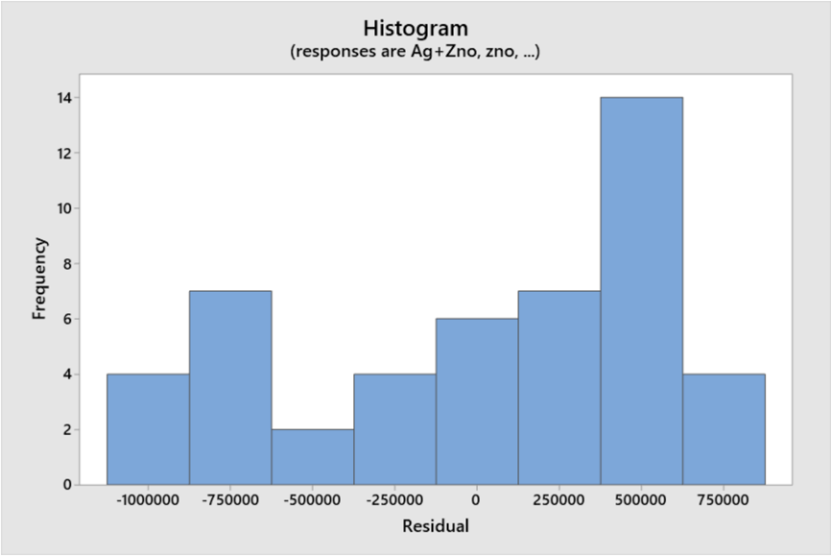
Histogram
(responses are Ag+Zno, zno, ...)
14
12
10
8
6
4
2
0
Frequency
-1000000
-750000
-500000
-250000
0
250000
500000
750000
Residual
