# Supplementary material for: The antibacterial effect of silver, zinc-oxide and combination of silver/ zinc oxide nanoparticles coating of orthodontic brackets (an in vitro study)
Source: BMC Oral Health. 2022 Jun 9;22:230. doi: 10.1186/s12903-022-02263-6 (PMC9185939; doi:10.1186/s12903-022-02263-6)
Supplement: Supplementary file 2 — Additional file 2: Comparison of the CFU of the four groups on S. mutans at T2. [file 12903_2022_2263_MOESM2_ESM.pdf]

WORKSHEET 1

One-way ANOVA: Ag, Ag+ZnO, Zno, Control

Method

Null hypothesis	All means are equal
Alternative hypothesis	Not all means are equal
Significance level	α = 0.05

Equal variances were assumed for the analysis.

Factor Information

Factor	Levels	Values
Factor	4	Ag, Ag+ZnO, Zno, Control

Analysis of Variance

Source	DF	Adj SS	Adj MS	F-Value	P-Value
Factor	3	2.06437E+14	6.88123E+13	99.17	0.0000000000
Error	44	3.05318E+13	6.93905E+11		
Total	47	2.36969E+14			

Model Summary

S	R-sq	R-sq(adj)	R-sq(pred)
833010	87.12%	86.24%	84.67%

Means

Factor	N	Mean	StDev	95% CI
Ag	12	10066667	913866	(9582033, 10551300)
Ag+ZnO	12	7520000	705717	(7035366, 8004634)
Zno	12	11220000	997287	(10735366, 11704634)
Control	12	13260000	669216	(12775366, 13744634)

Pooled StDev = 833010

Tukey Pairwise Comparisons

Grouping Information Using the Tukey Method and 95% Confidence

Factor	N	Mean	Grouping			
Control	12	13260000	A			
Zno	12	11220000		B		
Ag	12	10066667			C	
Ag+ZnO	12	7520000				D

Means that do not share a letter are significantly different.

Tukey Simultaneous Tests for Differences of Means

Difference of Levels	Difference of Means	SE of Difference	95% CI	T-Value	Adjusted P-Value
Ag+ZnO - Ag	-2546667	340075	(-3455640, -1637693)	-7.49	0.00000991327
Zno - Ag	1153333	340075	(244360, 2062307)	3.39	0.00778847101
Control - Ag	3193333	340075	(2284360, 4102307)	9.39	0.00000990244
Zno - Ag+ZnO	3700000	340075	(2791027, 4608973)	10.88	0.00000990243
Control - Ag+ZnO	5740000	340075	(4831027, 6648973)	16.88	0.00000990243
Control - Zno	2040000	340075	(1131027, 2948973)	6.00	0.00001182060

Individual confidence level = 98.95%

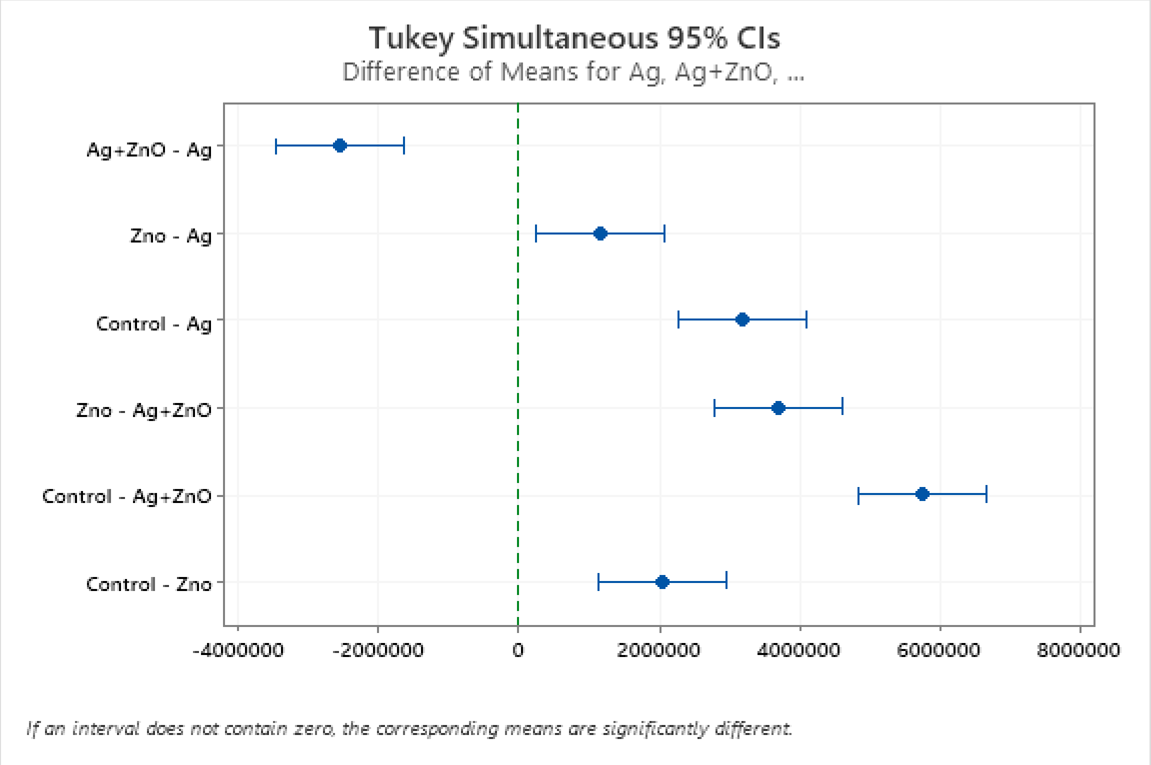

Fisher Pairwise Comparisons

Grouping Information Using the Fisher LSD Method and 95% Confidence

Factor	N	Mean	Grouping			
Control	12	13260000	A			
Zno	12	11220000		B		
Ag	12	10066667			C	
Ag+ZnO	12	7520000				D

Means that do not share a letter are significantly different.

Fisher Individual Tests for Differences of Means

Difference of Levels	Difference of Means	SE of Difference	95% CI	T-Value	Adjusted P-Value

Ag+ZnO - Ag	-2546667	340075	(-3232042, -1861291)	-7.49	0.000
Zno - Ag	1153333	340075	(467958, 1838709)	3.39	0.001
Control - Ag	3193333	340075	(2507958, 3878709)	9.39	0.000
Zno - Ag+ZnO	3700000	340075	(3014624, 4385376)	10.88	0.000
Control - Ag+ZnO	5740000	340075	(5054624, 6425376)	16.88	0.000
Control - Zno	2040000	340075	(1354624, 2725376)	6.00	0.000

Simultaneous confidence level = 80.20%

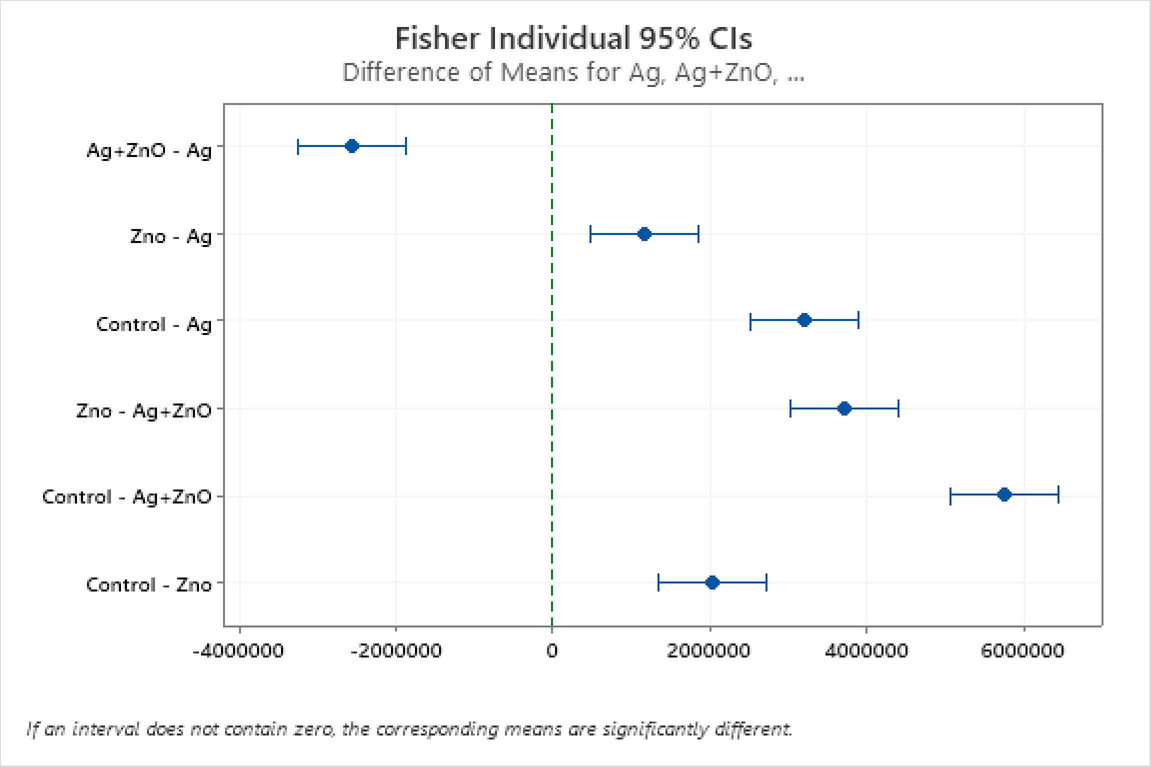

Dunnett Multiple Comparisons with a Control

Grouping Information Using the Dunnett Method and 95% Confidence

Factor	N	Mean	Grouping
Ag (control)	12	10066667	A
Control	12	13260000	
Zno	12	11220000	
Ag+ZnO	12	7520000	

Means not labeled with the letter A are significantly different from the control level mean.

Dunnett Simultaneous Tests for Level Mean - Control Mean

Difference of Levels	Difference of Means	SE of Difference	95% CI	T-Value	Adjusted P-Value
Ag+ZnO - Ag	-2546667	340075	(-3374039, -1719295)	-7.49	0.000
Zno - Ag	1153333	340075	(325961, 1980705)	3.39	0.004
Control - Ag	3193333	340075	(2365961, 4020705)	9.39	0.000

Individual confidence level = 98.09%

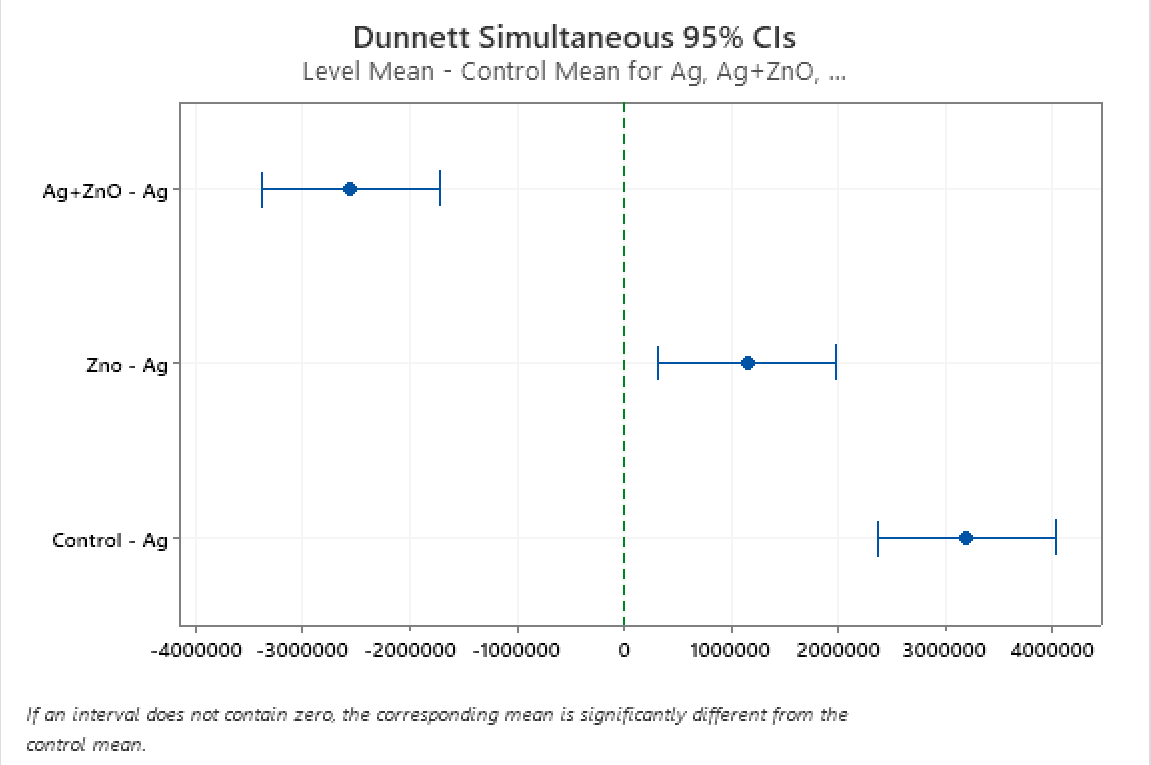

Hsu Multiple Comparisons with the Best (MCB)

Hsu Simultaneous Tests for Level Mean - Largest of Other Level Means

Difference of Levels	Difference of Means	SE of Difference	95% CI	T-Value	Adjusted P-Value
Ag - Control	-3193333	340075	(-3914162, 0)	-9.39	0.000
Ag+ZnO - Control	-5740000	340075	(-6460828, 0)	-16.88	0.000
Zno - Control	-2040000	340075	(-2760828, 0)	-6.00	0.000
Control - Zno	2040000	340075	(0, 2760828)	6.00	0.000

Individual confidence level = 96.03%

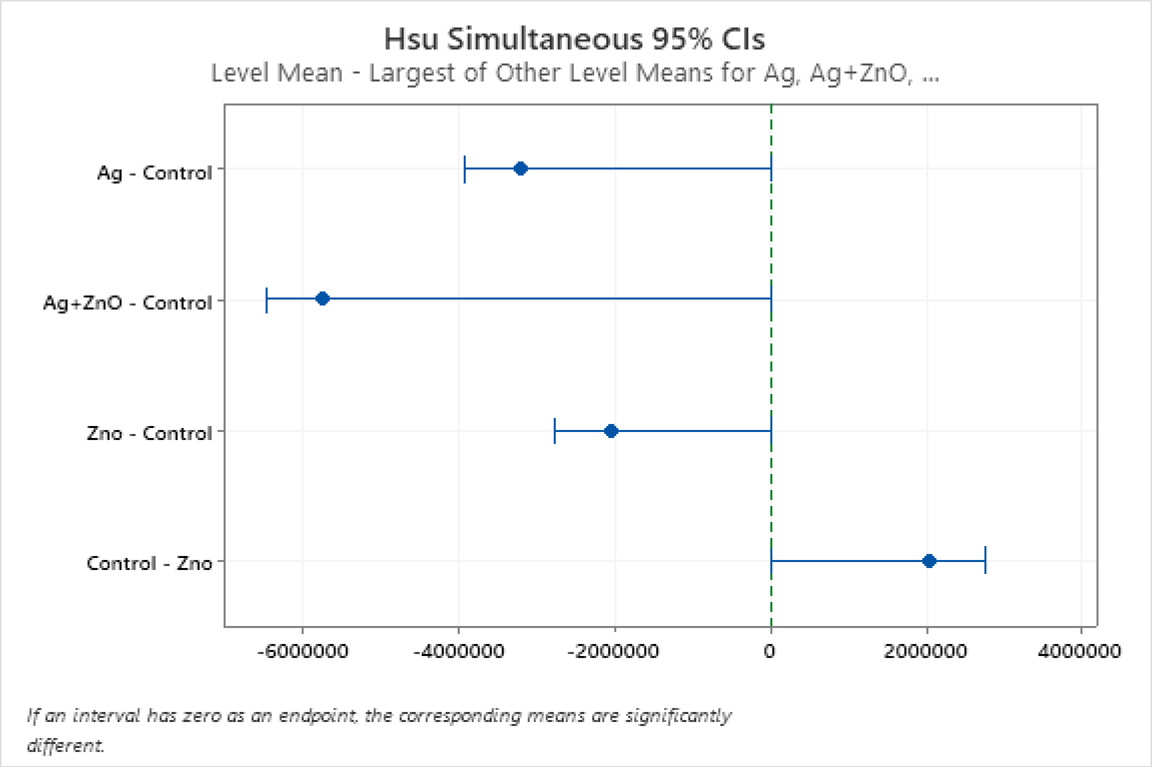
Hsu Simultaneous 95% CIs
Level Mean - Largest of Other Level Means for Ag, Ag+ZnO, ...
Ag - Control
Ag+ZnO - Control
Zno - Control
Control - Zno
-6000000
-4000000
-2000000
0
2000000
4000000
If an interval has zero as an endpoint, the corresponding means are significantly different.

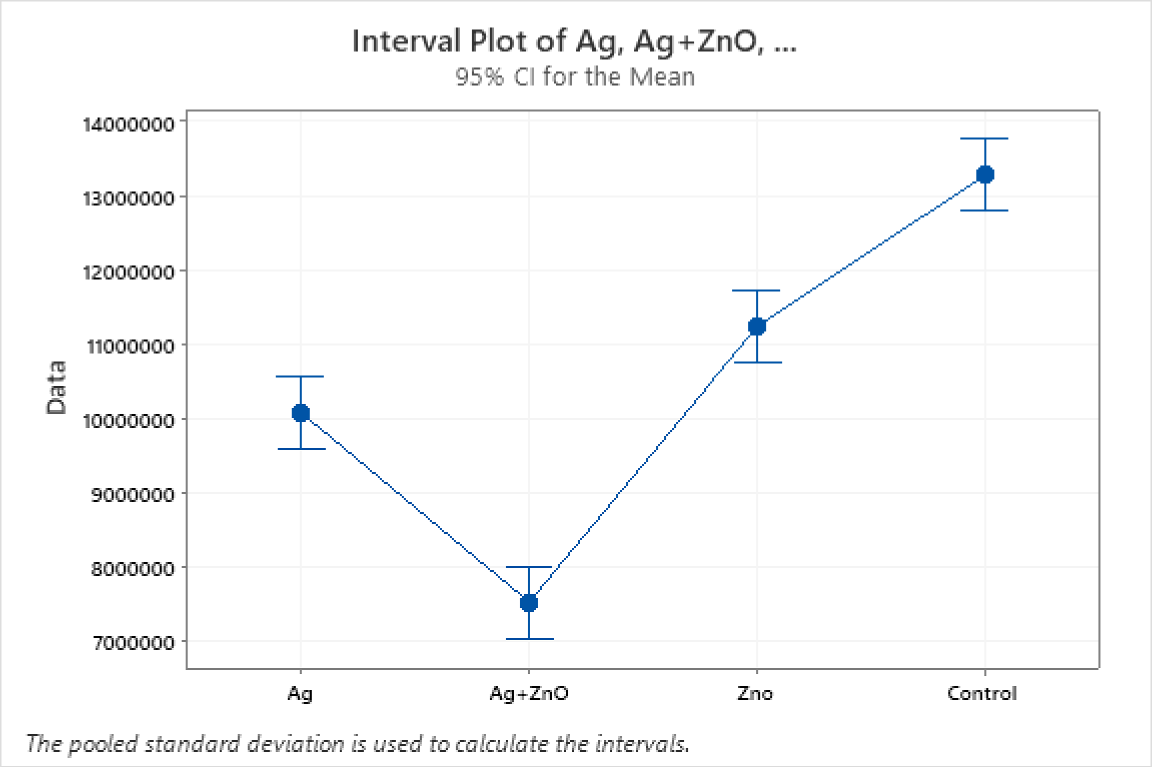
Interval Plot of Ag, Ag+ZnO, ...
95% CI for the Mean
Data
14000000
13000000
12000000
11000000
10000000
9000000
8000000
7000000
Ag
Ag+ZnO
Zno
Control
The pooled standard deviation is used to calculate the intervals.

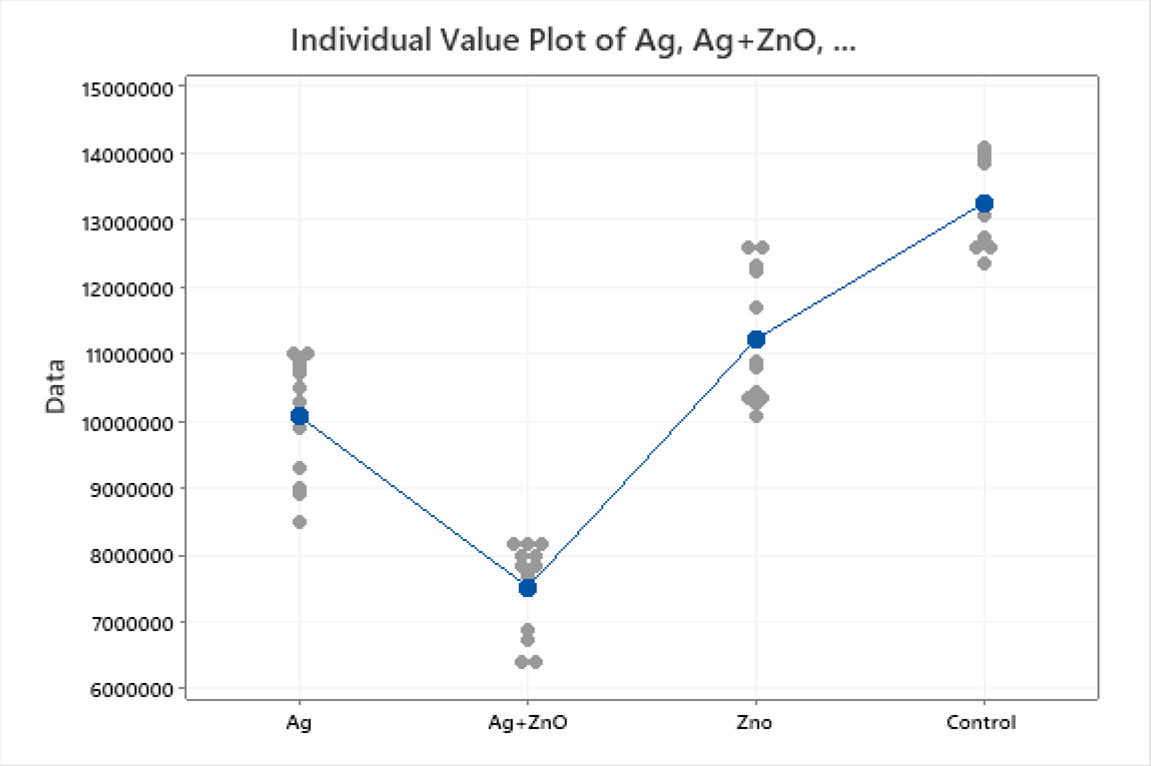
Individual Value Plot of Ag, Ag+ZnO, ...
Data
15000000
14000000
13000000
12000000
11000000
10000000
9000000
8000000
7000000
6000000
Ag
Ag+ZnO
Zno
Control

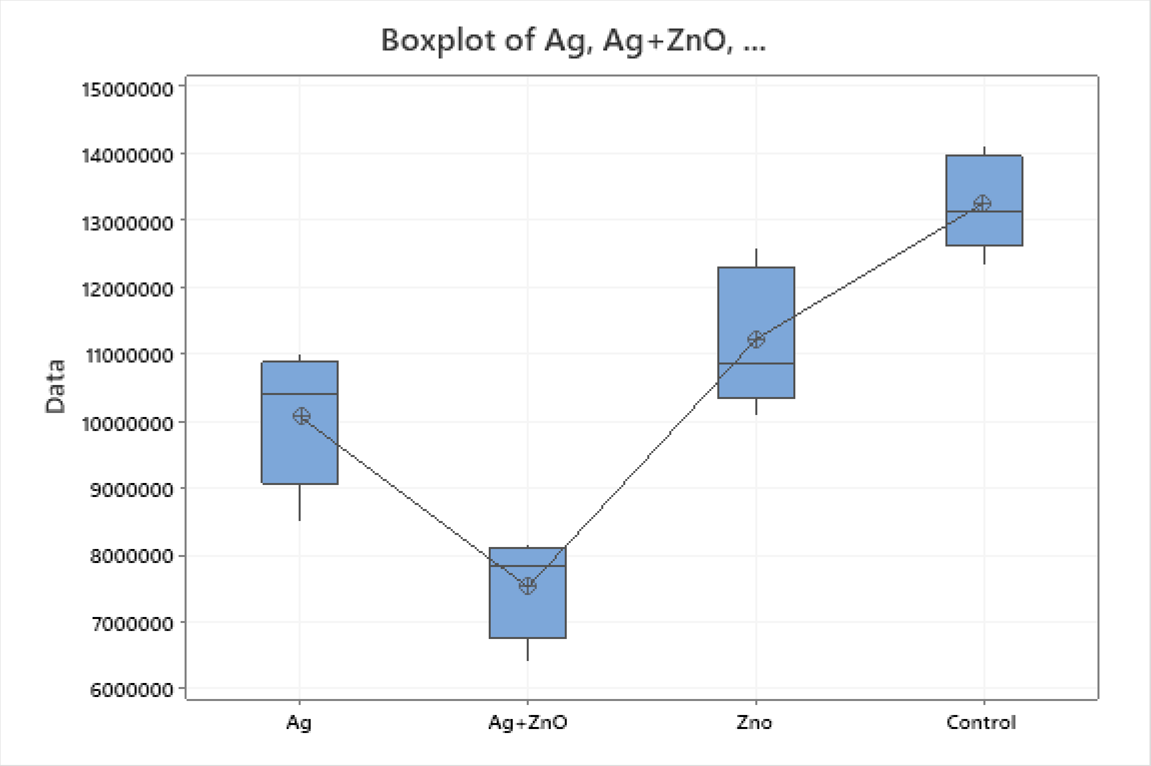
Boxplot of Ag, Ag+ZnO, ...
Data
15000000
14000000
13000000
12000000
11000000
10000000
9000000
8000000
7000000
6000000
Ag
Ag+ZnO
Zno
Control

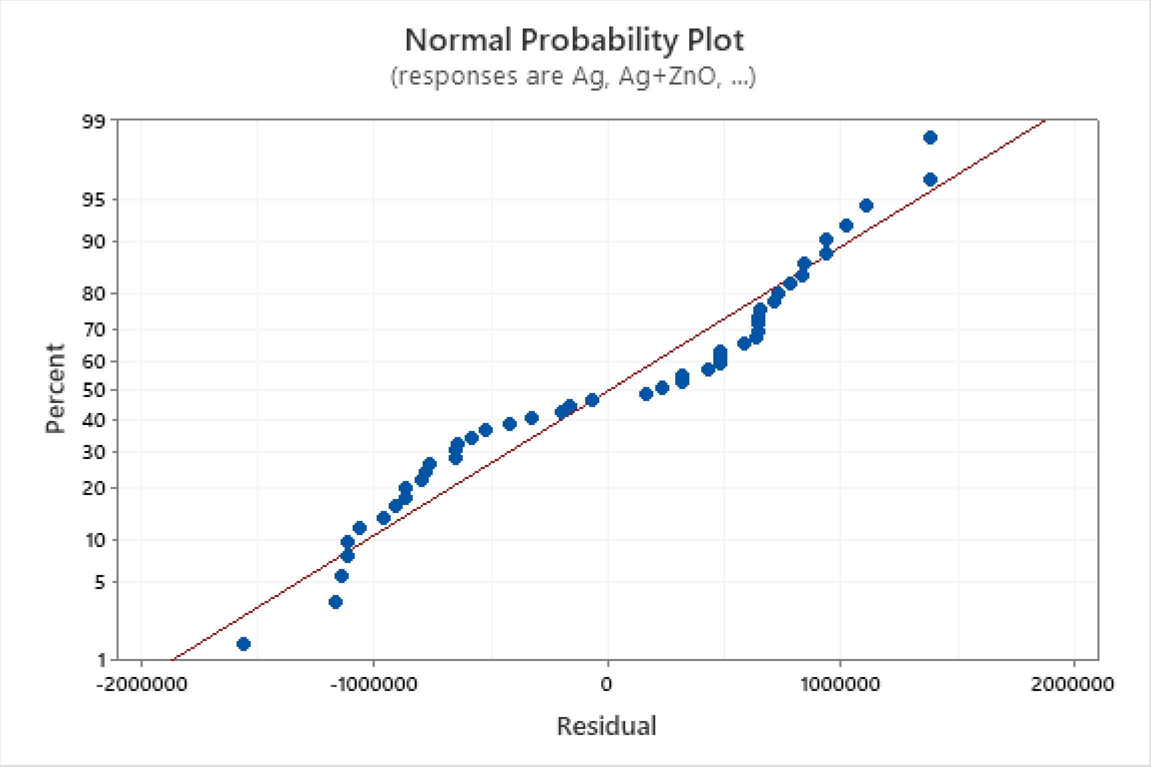
Normal Probability Plot
(responses are Ag, Ag+ZnO, ...)
Percent
99
95
90
80
70
60
50
40
30
20
10
5
1
-2000000
-1000000
0
1000000
2000000
Residual

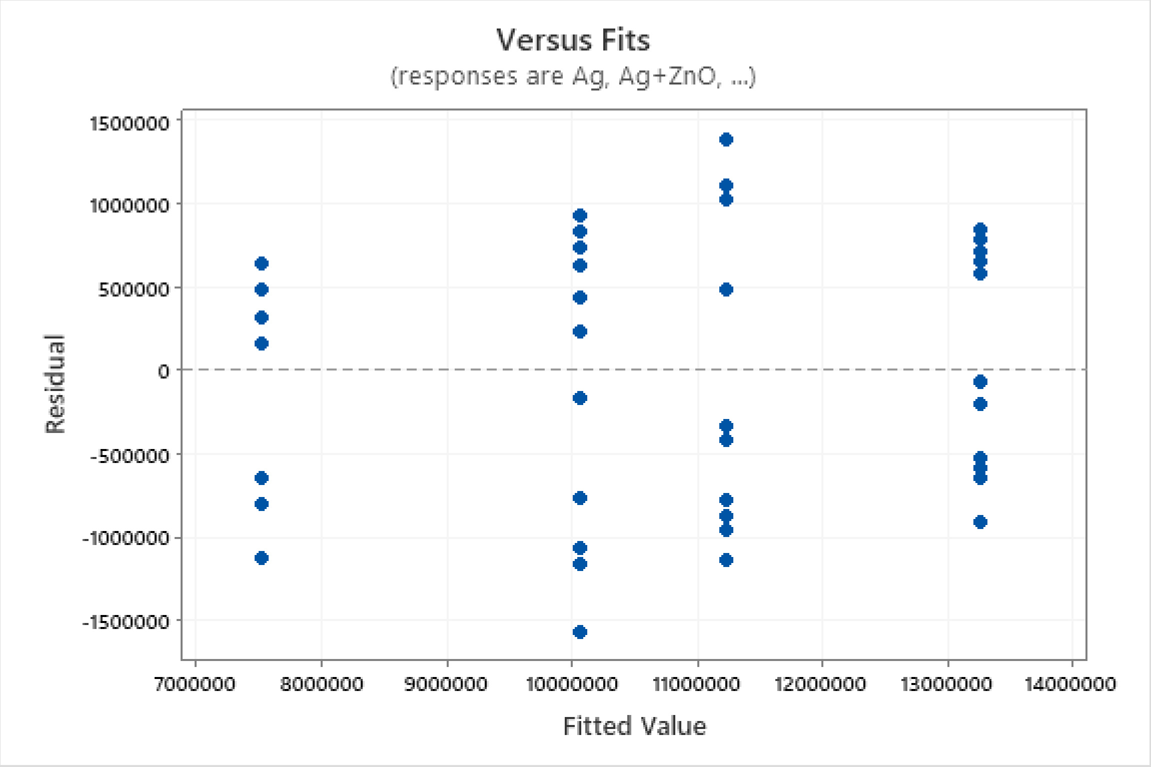
Versus Fits
(responses are Ag, Ag+ZnO, ...)
Residual
1500000
1000000
500000
0
-500000
-1000000
-1500000
7000000
8000000
9000000
10000000
11000000
12000000
13000000
14000000
Fitted Value

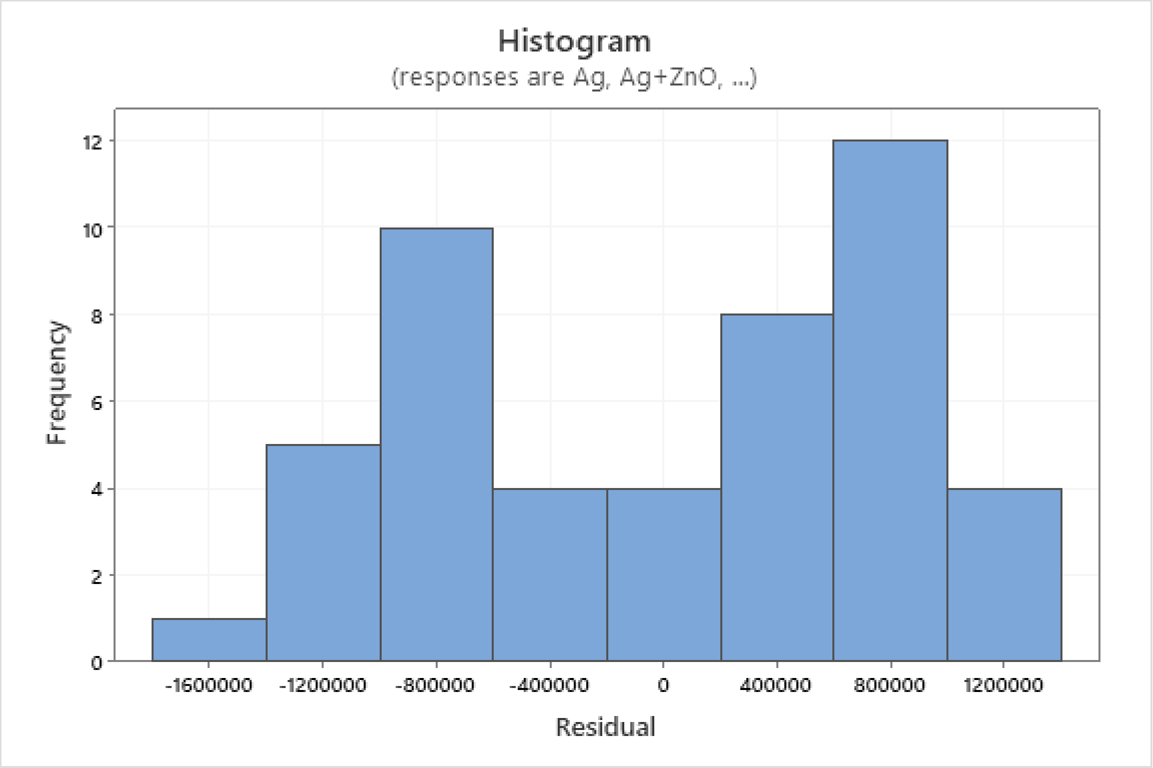
Histogram
(responses are Ag, Ag+ZnO, ...)
Frequency
12
10
8
6
4
2
0
-1600000
-1200000
-800000
-400000
0
400000
800000
1200000
Residual
