# Supplementary material for: The antibacterial effect of silver, zinc-oxide and combination of silver/ zinc oxide nanoparticles coating of orthodontic brackets (an in vitro study)
Source: BMC Oral Health. 2022 Jun 9;22:230. doi: 10.1186/s12903-022-02263-6 (PMC9185939; doi:10.1186/s12903-022-02263-6)
Supplement: Supplementary file 3 — Additional file 3: Comparison of the CFU of the four groups on L. acidophilus at T1. [file 12903_2022_2263_MOESM3_ESM.pdf]

WORKSHEET 1

One-way ANOVA: Control, Ag, Zn0, Ag/Zno

Method

Null hypothesis	All means are equal
Alternative hypothesis	Not all means are equal
Significance level	α = 0.05

Equal variances were assumed for the analysis.

Factor Information

Factor	Levels	Values
Factor	4	Control, Ag, Zn0, Ag/Zno

Analysis of Variance

Source	DF	Seq SS	Contribution	Adj SS	Adj MS	F-Value	P-Value
Factor	3	1.92727E+12	94.41%	1.92727E+12	6.42422E+11	247.65	0.000
Error	44	1.14141E+11	5.59%	1.14141E+11	2594105114		
Total	47	2.04141E+12	100.00%				

Model Summary

S	R-sq	R-sq(adj)	PRESS	R-sq(pred)
50932.4	94.41%	94.03%	1.35837E+11	93.35%

Means

Factor	N	Mean	StDev	95% CI
Control	12	650000	73855	(620368, 679632)
Ag	12	246875	33763	(217243, 276507)
Zn0	12	462500	58387	(432868, 492132)
Ag/Zno	12	128125	19310	(98493, 157757)

Pooled StDev = 50932.4

Tukey Pairwise Comparisons

Grouping Information Using the Tukey Method and 95% Confidence

Factor	N	Mean	Grouping			
Control	12	650000	A			
Zn0	12	462500		B		
Ag	12	246875			C	
Ag/Zno	12	128125				D

Means that do not share a letter are significantly different.

Tukey Simultaneous Tests for Differences of Means

Difference of Levels	Difference of Means	SE of Difference	95% CI	T-Value	Adjusted P-Value

Ag - Control	-403125	20793	(-458702, -347548)	-19.39	0.000009902
Zn0 - Control	-187500	20793	(-243077, -131923)	-9.02	0.000009902
Ag/Zno - Control	-521875	20793	(-577452, -466298)	-25.10	0.000009902
Zn0 - Ag	215625	20793	(160048, 271202)	10.37	0.000009902
Ag/Zno - Ag	-118750	20793	(-174327, -63173)	-5.71	0.000014989
Ag/Zno - Zn0	-334375	20793	(-389952, -278798)	-16.08	0.000009902

Individual confidence level = 98.95%

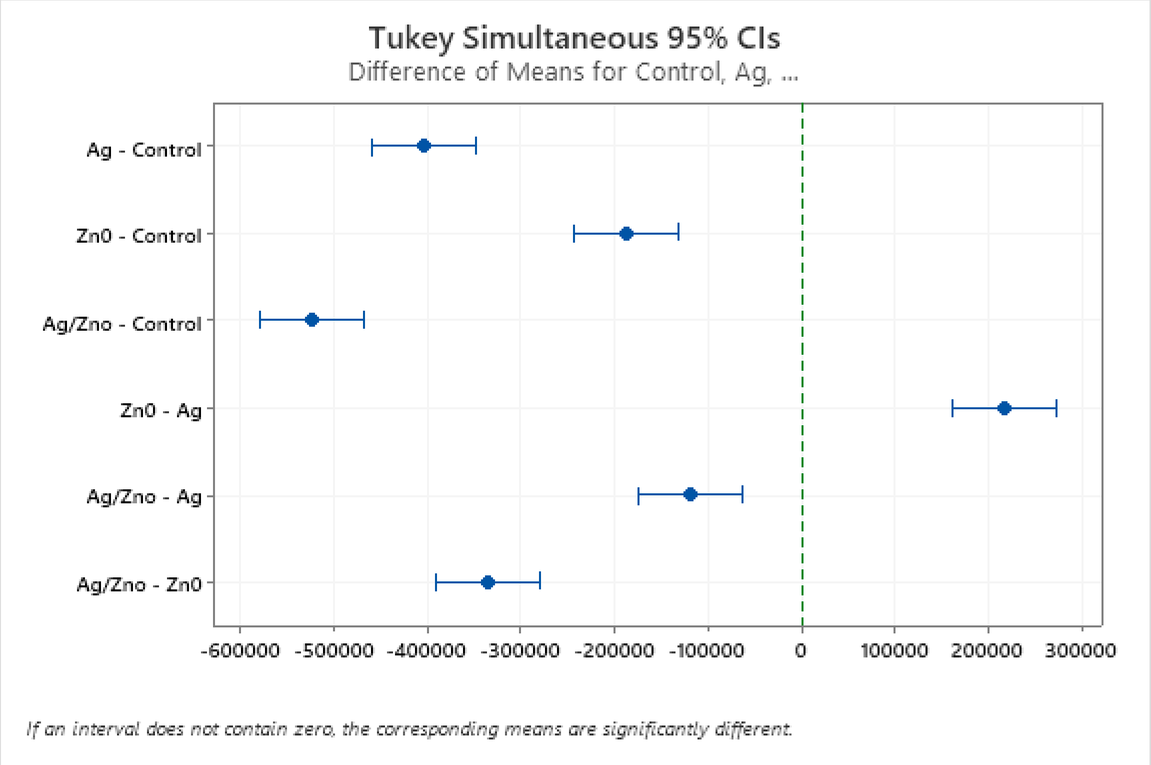

Fisher Pairwise Comparisons

Grouping Information Using the Fisher LSD Method and 95% Confidence

Factor	N	Mean	Grouping
Control	12	650000	A
Zn0	12	462500	B
Ag	12	246875	C
Ag/Zno	12	128125	D

Means that do not share a letter are significantly different.

Fisher Individual Tests for Differences of Means

Difference of Levels	Difference of Means	SE of Difference	95% CI	T-Value	Adjusted P-Value
Ag - Control	-403125	20793	(-445031, -361219)	-19.39	0.000
Zn0 - Control	-187500	20793	(-229406, -145594)	-9.02	0.000

Ag/Zno - Control	-521875	20793	(-563781, -479969)	-25.10	0.000
Zn0 - Ag	215625	20793	(173719, 257531)	10.37	0.000
Ag/Zno - Ag	-118750	20793	(-160656, -76844)	-5.71	0.000
Ag/Zno - Zn0	-334375	20793	(-376281, -292469)	-16.08	0.000

Simultaneous confidence level = 80.20%

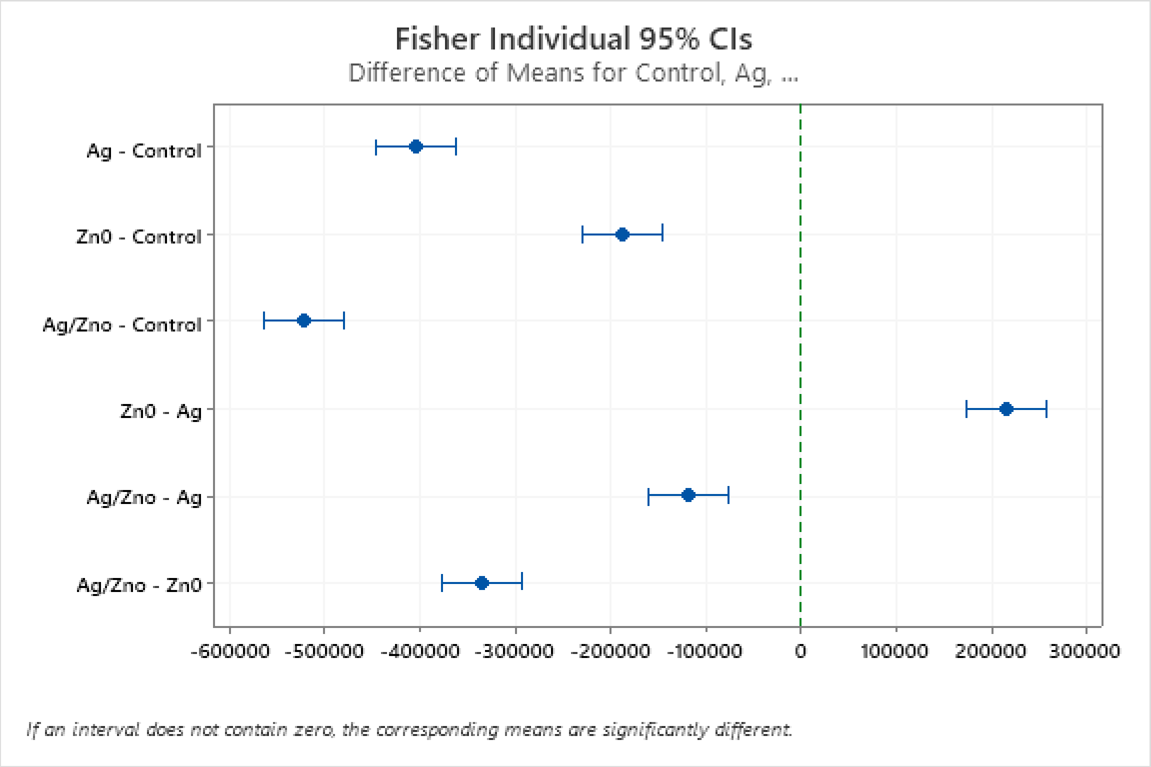

Dunnett Multiple Comparisons with a Control

Grouping Information Using the Dunnett Method and 95% Confidence

Factor	N	Mean	Grouping
Control (control)	12	650000	A
Zn0	12	462500	
Ag	12	246875	
Ag/Zno	12	128125	

Means not labeled with the letter A are significantly different from the control level mean.

Dunnett Simultaneous Tests for Level Mean - Control Mean

Difference of Levels	Difference of Means	SE of Difference	95% CI	T-Value	Adjusted P-Value
Ag - Control	-403125	20793	(-453713, -352537)	-19.39	0.000
Zn0 - Control	-187500	20793	(-238088, -136912)	-9.02	0.000
Ag/Zno - Control	-521875	20793	(-572463, -471287)	-25.10	0.000

Individual confidence level = 98.09%

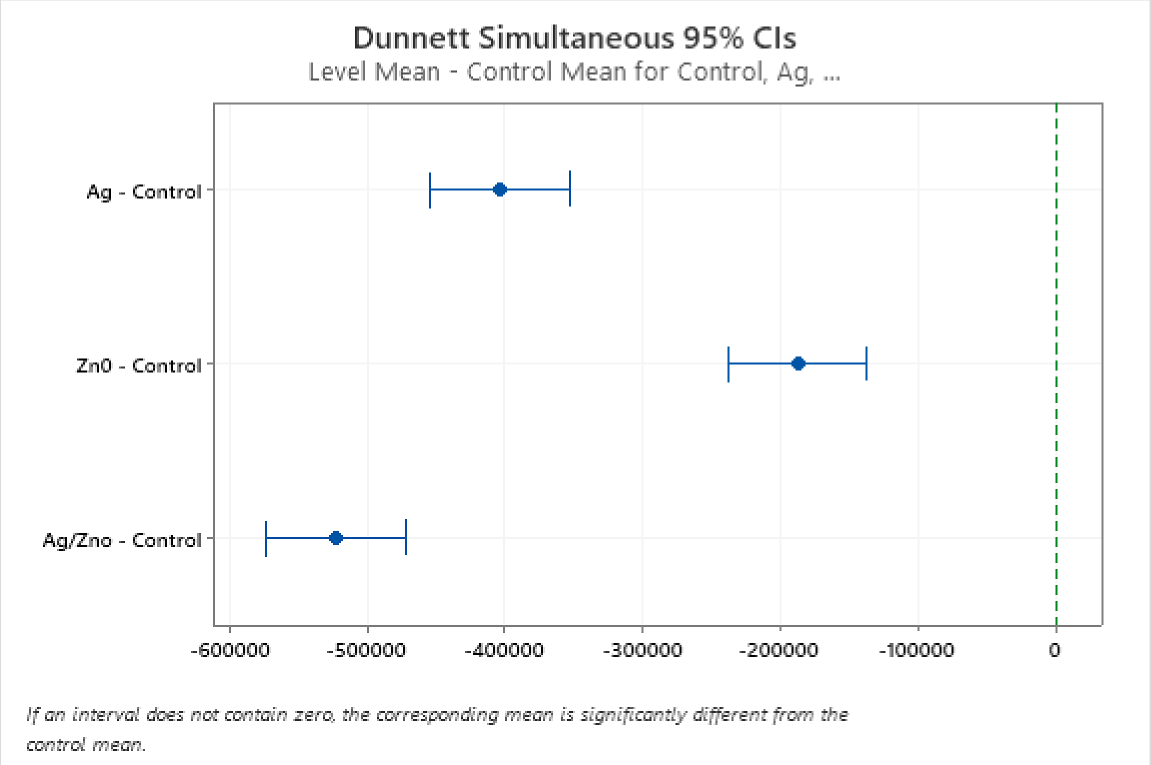

Hsu Multiple Comparisons with the Best (MCB)

Hsu Simultaneous Tests for Level Mean - Largest of Other Level Means

Difference of Levels	Difference of Means	SE of Difference	95% CI	T-Value	Adjusted P-Value
Control - Zn0	187500	20793	(0, 231573)	9.02	0.000
Ag - Control	-403125	20793	(-447198, 0)	-19.39	0.000
Zn0 - Control	-187500	20793	(-231573, 0)	-9.02	0.000
Ag/Zno - Control	-521875	20793	(-565948, 0)	-25.10	0.000

Individual confidence level = 96.03%

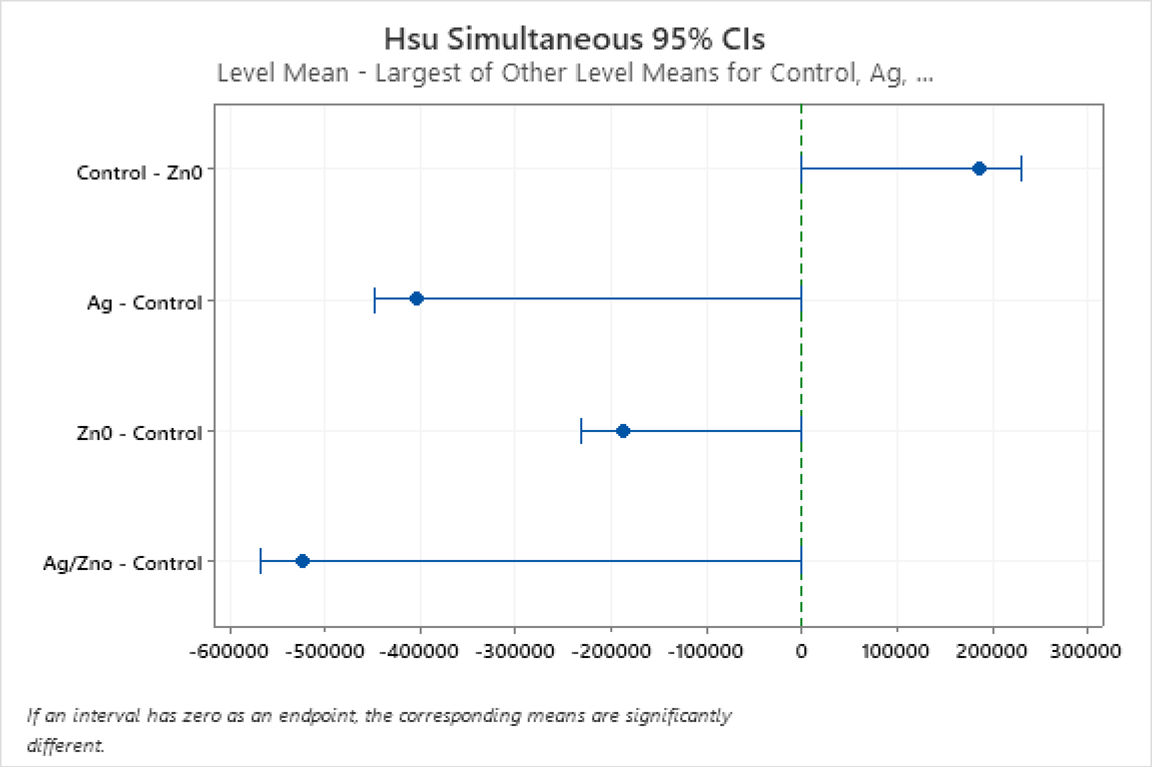
Hsu Simultaneous 95% CIs
Level Mean - Largest of Other Level Means for Control, Ag, ...
Control - Zn0
Ag - Control
Zn0 - Control
Ag/Zno - Control
-600000
-500000
-400000
-300000
-200000
-100000
0
100000
200000
300000
If an interval has zero as an endpoint, the corresponding means are significantly different.

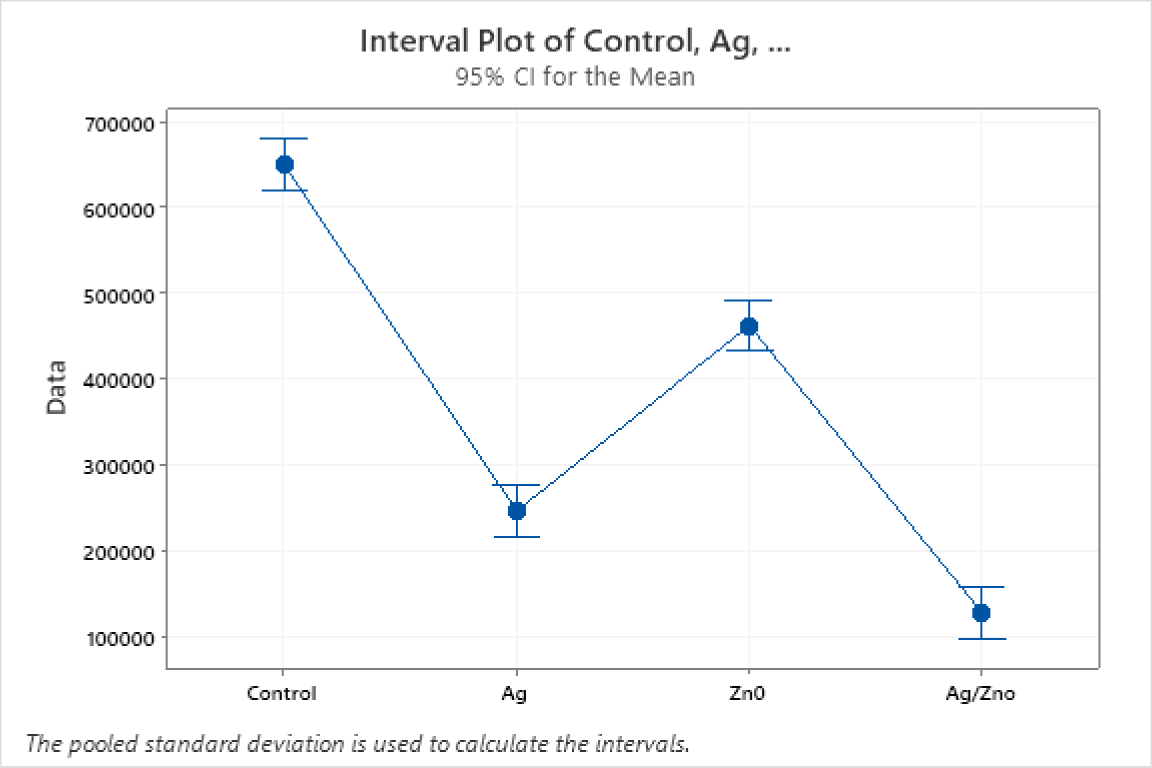
Interval Plot of Control, Ag, ...
95% CI for the Mean
Data
700000
600000
500000
400000
300000
200000
100000
Control
Ag
Zn0
Ag/Zno
The pooled standard deviation is used to calculate the intervals.

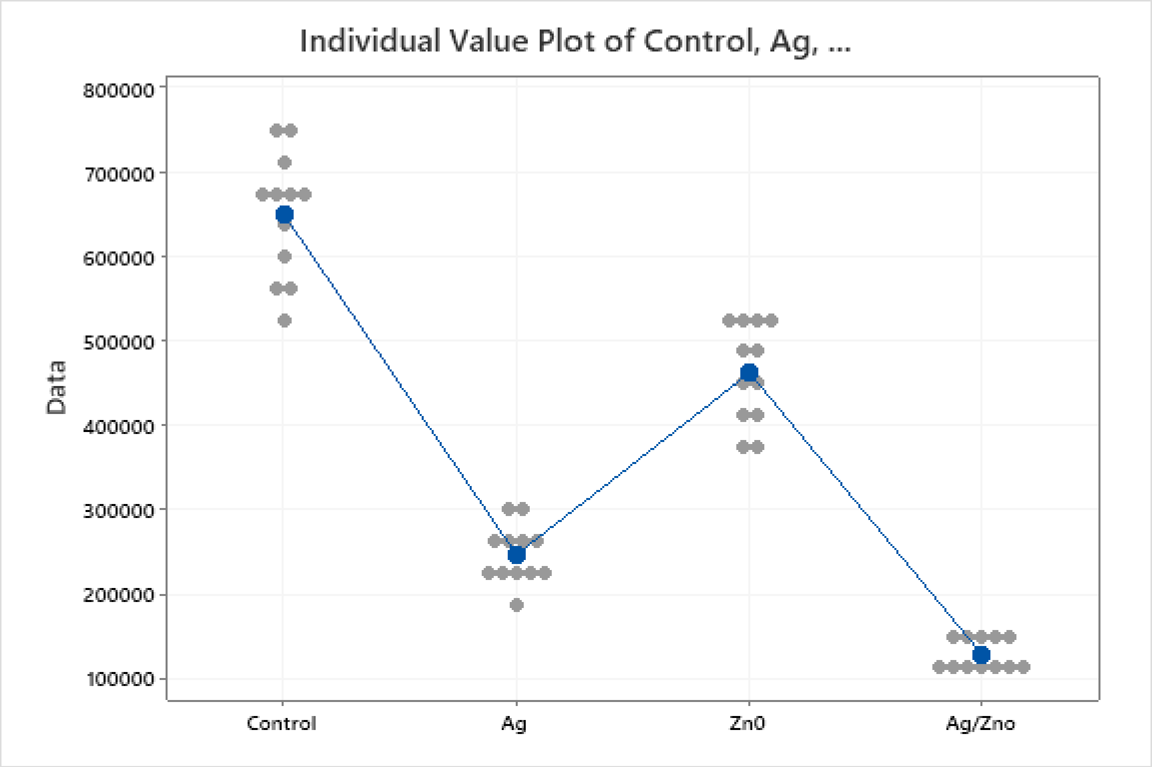
Individual Value Plot of Control, Ag, ...
800000
700000
600000
500000
400000
300000
200000
100000
Data
Control
Ag
Zn0
Ag/Zno

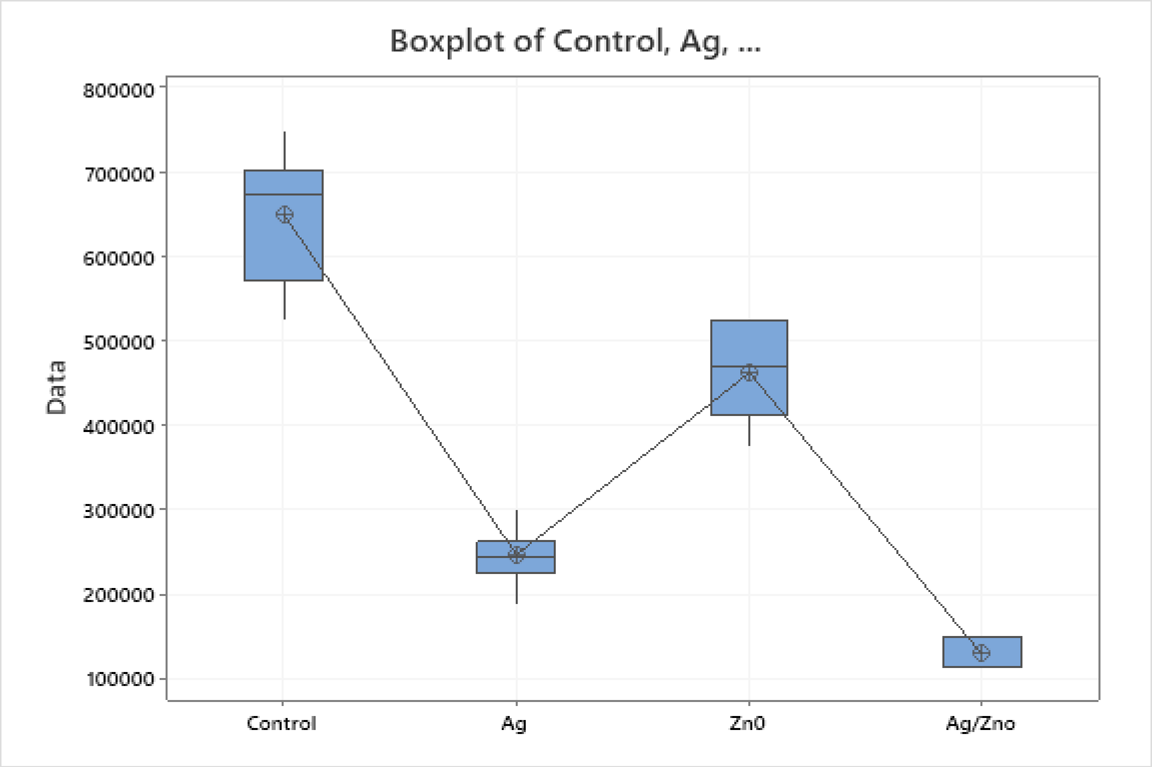
Boxplot of Control, Ag, ...
800000
700000
600000
500000
400000
300000
200000
100000
Data
Control
Ag
Zn0
Ag/Zno

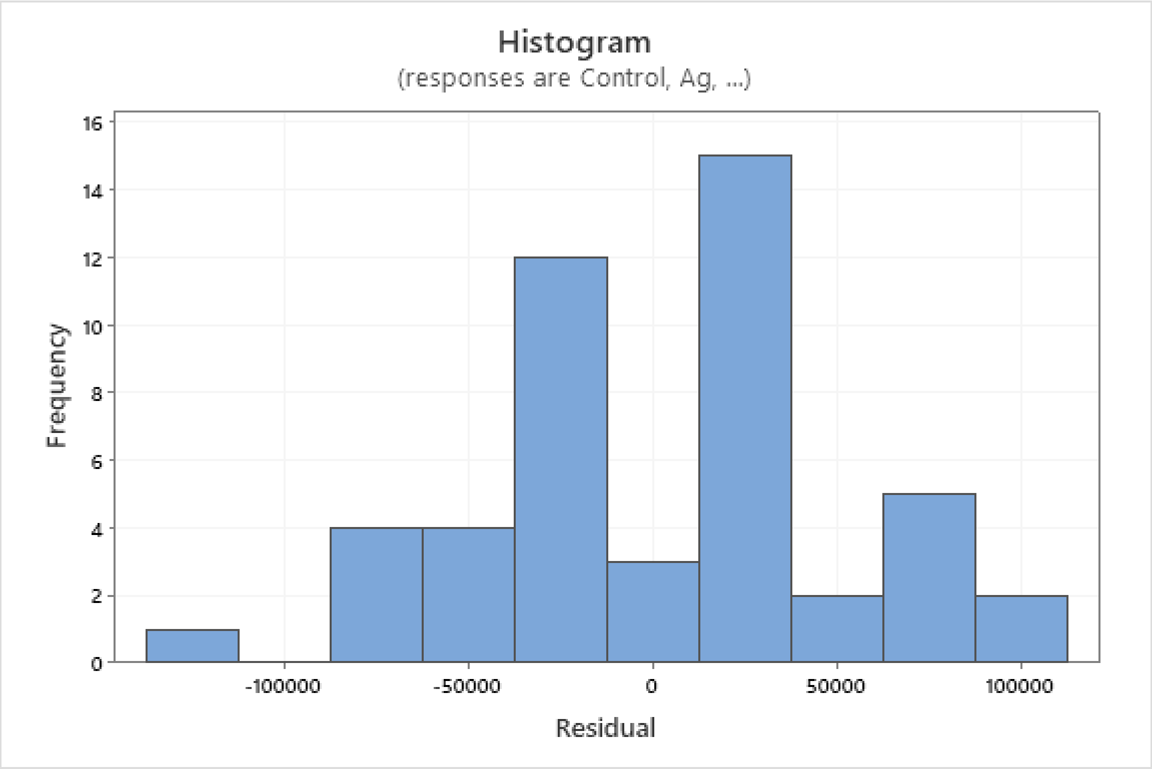
Histogram
(responses are Control, Ag, ...)
Frequency
Residual
0
2
4
6
8
10
12
14
16
-100000
-50000
0
50000
100000
