# Supplementary material for: The antibacterial effect of silver, zinc-oxide and combination of silver/ zinc oxide nanoparticles coating of orthodontic brackets (an in vitro study)
Source: BMC Oral Health. 2022 Jun 9;22:230. doi: 10.1186/s12903-022-02263-6 (PMC9185939; doi:10.1186/s12903-022-02263-6)
Supplement: Supplementary file 4 — Additional file 4: Comparison of the CFU of the four groups on L. acidophilus at T2. [file 12903_2022_2263_MOESM4_ESM.pdf]

WORKSHEET 1

One-way ANOVA: Control, Ag, Zn0, Ag/Zno

Method

Null hypothesis	All means are equal
Alternative hypothesis	Not all means are equal
Significance level	α = 0.05

Equal variances were assumed for the analysis.

Factor Information

Factor	Levels	Values
Factor	4	Control, Ag, Zn0, Ag/Zno

Analysis of Variance

Source	DF	Seq SS	Contribution	Adj SS	Adj MS	F-Value	P-Value
Factor	3	2.35723E+12	95.17%	2.35723E+12	7.85742E+11	289.24	0.000
Error	44	1.19531E+11	4.83%	1.19531E+11	2716619318		
Total	47	2.47676E+12	100.00%				

Model Summary

S	R-sq	R-sq(adj)	PRESS	R-sq(pred)
52121.2	95.17%	94.84%	1.42252E+11	94.26%

Means

Factor	N	Mean	StDev	95% CI
Control	12	712500	79950	(682177, 742823)
Ag	12	262500	27696	(232177, 292823)
Zn0	12	487500	57653	(457177, 517823)
Ag/Zno	12	131250	19584	(100927, 161573)

Pooled StDev = 52121.2

Tukey Pairwise Comparisons

Grouping Information Using the Tukey Method and 95% Confidence

Factor	N	Mean	Grouping			
Control	12	712500	A			
Zn0	12	487500		B		
Ag	12	262500			C	
Ag/Zno	12	131250				D

Means that do not share a letter are significantly different.

Tukey Simultaneous Tests for Differences of Means

Difference of Levels	Difference of Means	SE of Difference	95% CI	T-Value	Adjusted P-Value
Ag - Control	-450000	21278	(-506874, -393126)	-21.15	0.000009902
Zn0 - Control	-225000	21278	(-281874, -168126)	-10.57	0.000009902
Ag/Zno - Control	-581250	21278	(-638124, -524376)	-27.32	0.000009902
Zn0 - Ag	225000	21278	(168126, 281874)	10.57	0.000009902
Ag/Zno - Ag	-131250	21278	(-188124, -74376)	-6.17	0.000010974
Ag/Zno - Zn0	-356250	21278	(-413124, -299376)	-16.74	0.000009902

Individual confidence level = 98.95%

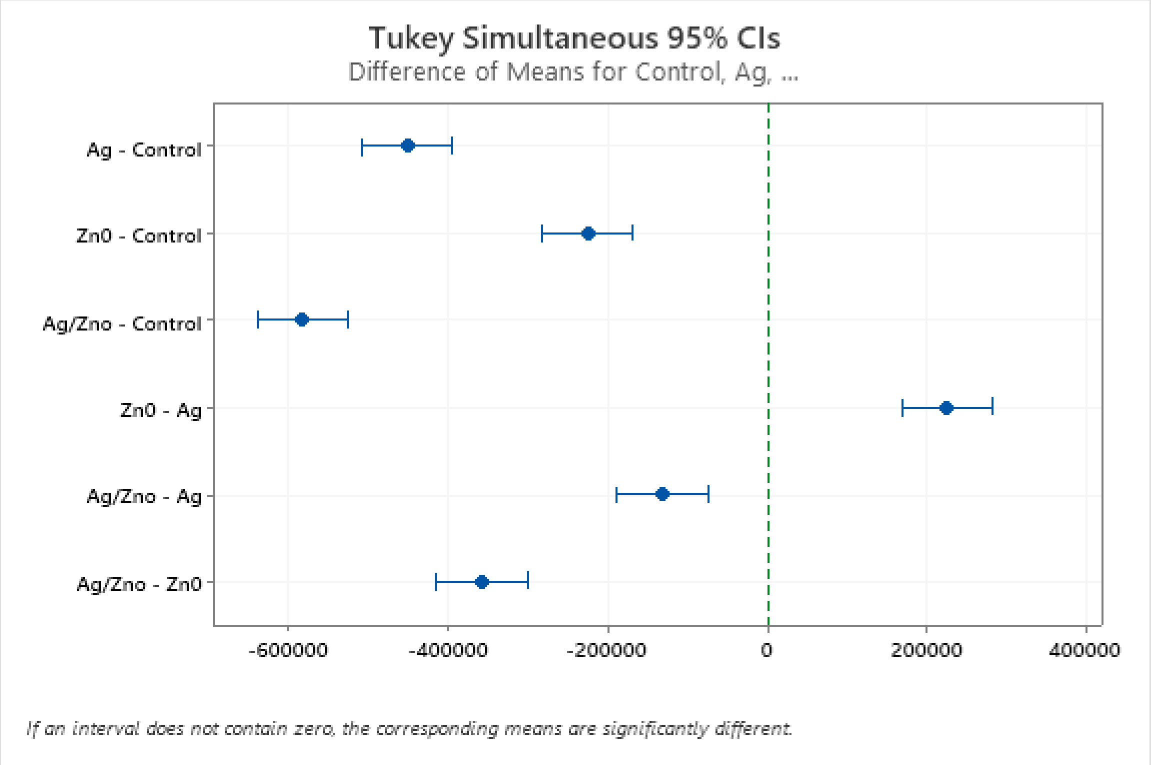

Fisher Pairwise Comparisons

Grouping Information Using the Fisher LSD Method and 95% Confidence

Factor	N	Mean	Grouping			
Control	12	712500	A			
Zn0	12	487500		B		
Ag	12	262500			C	
Ag/Zno	12	131250				D

Means that do not share a letter are significantly different.

Fisher Individual Tests for Differences of Means

Difference of Levels	Difference of Means	SE of Difference	95% CI	T-Value	Adjusted P-Value
Ag - Control	-450000	21278	(-492884, -407116)	-21.15	0.000

Zn0 - Control	-225000	21278	(-267884, -182116)	-10.57	0.000
Ag/Zno - Control	-581250	21278	(-624134, -538366)	-27.32	0.000
Zn0 - Ag	225000	21278	(182116, 267884)	10.57	0.000
Ag/Zno - Ag	-131250	21278	(-174134, -88366)	-6.17	0.000
Ag/Zno - Zn0	-356250	21278	(-399134, -313366)	-16.74	0.000

Simultaneous confidence level = 80.20%

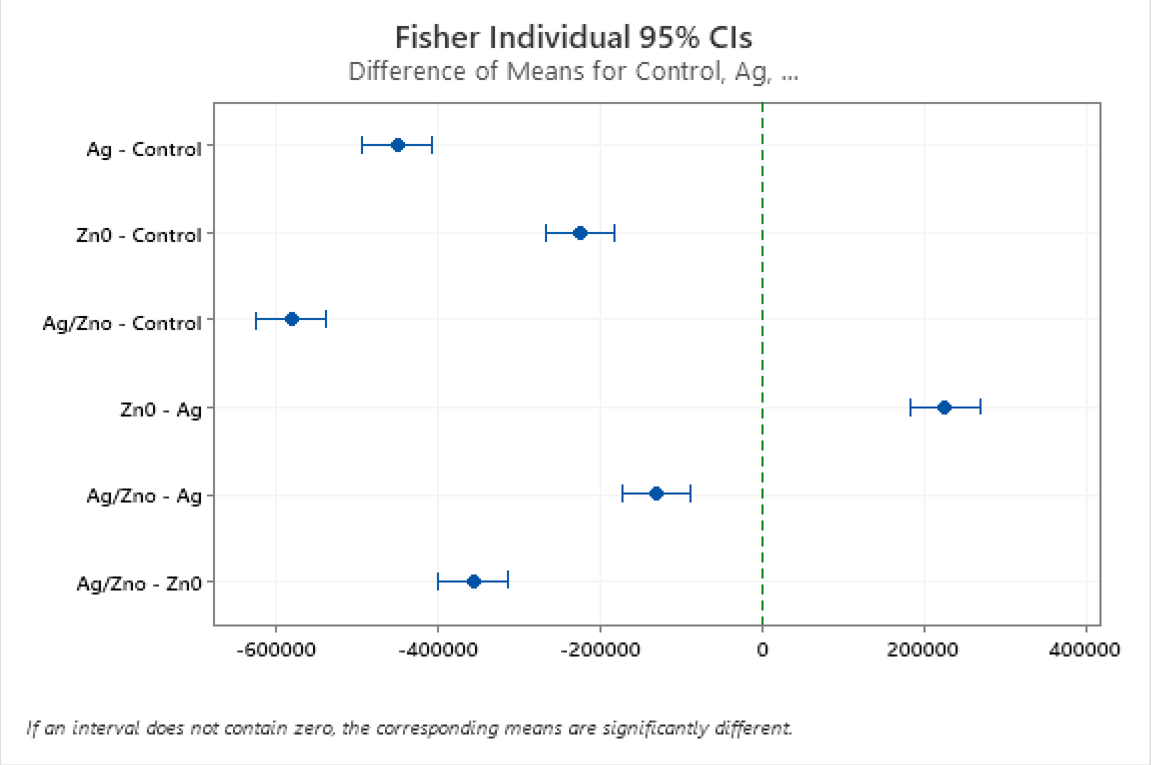

Dunnett Multiple Comparisons with a Control

Grouping Information Using the Dunnett Method and 95% Confidence

Factor	N	Mean	Grouping
Control (control)	12	712500	A
Zn0	12	487500	
Ag	12	262500	
Ag/Zno	12	131250	

Means not labeled with the letter A are significantly different from the control level mean.

Dunnett Simultaneous Tests for Level Mean - Control Mean

Difference of Levels	Difference of Means	SE of Difference	95% CI	T-Value	Adjusted P-Value
Ag - Control	-450000	21278	(-501768, -398232)	-21.15	0.000
Zn0 - Control	-225000	21278	(-276768, -173232)	-10.57	0.000

Ag/Zno - Control	-581250	21278	(-633018, -529482)	-27.32	0.000

Individual confidence level = 98.09%

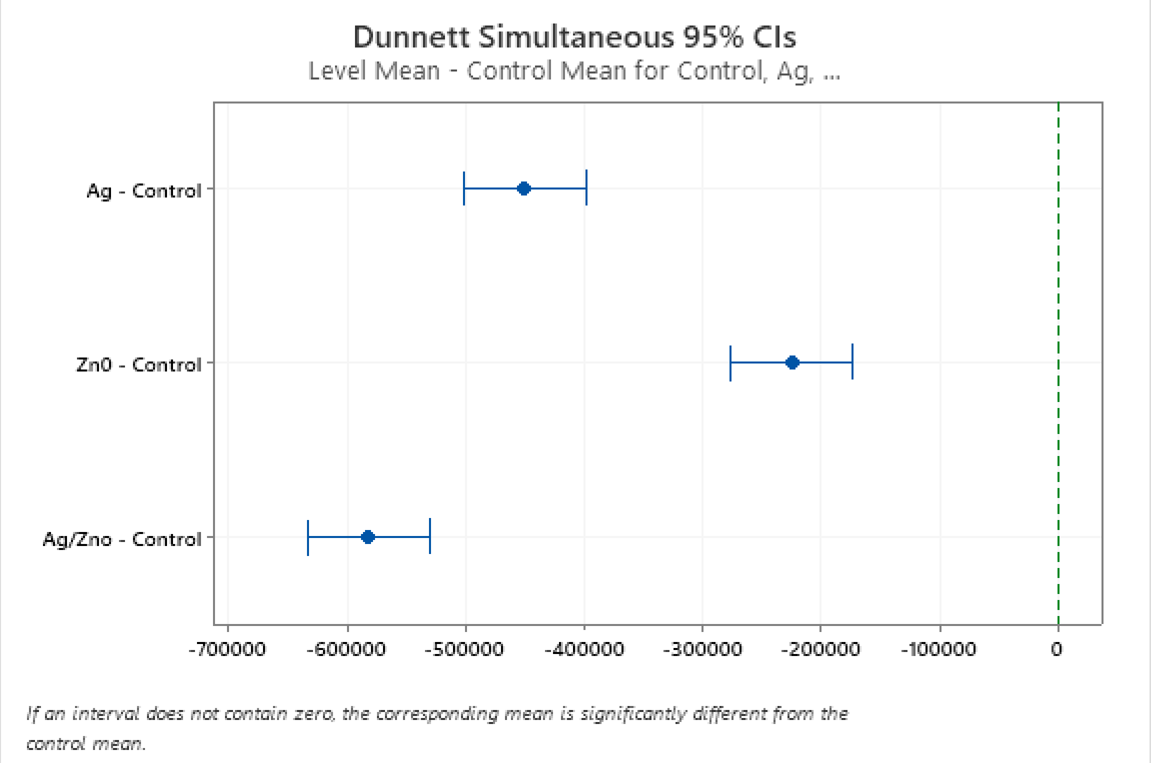

Hsu Multiple Comparisons with the Best (MCB)

Hsu Simultaneous Tests for Level Mean - Largest of Other Level Means

Difference of Levels	Difference of Means	SE of Difference	95% CI	T-Value	Adjusted P-Value
Control - Zn0	225000	21278	(0, 270102)	10.57	0.000
Ag - Control	-450000	21278	(-495102, 0)	-21.15	0.000
Zn0 - Control	-225000	21278	(-270102, 0)	-10.57	0.000
Ag/Zno - Control	-581250	21278	(-626352, 0)	-27.32	0.000

Individual confidence level = 96.03%

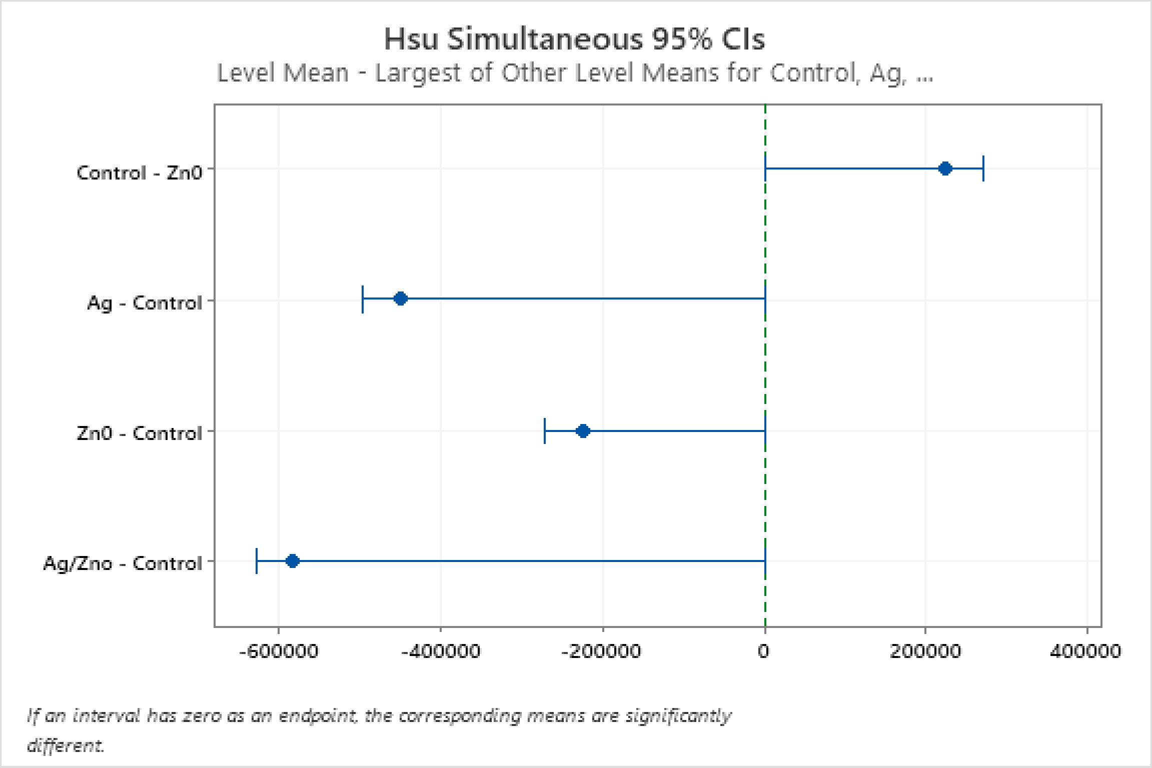
Hsu Simultaneous 95% CIs
Level Mean - Largest of Other Level Means for Control, Ag, ...
Control - Zn0
Ag - Control
Zn0 - Control
Ag/Zno - Control
-600000
-400000
-200000
0
200000
400000
If an interval has zero as an endpoint, the corresponding means are significantly different.

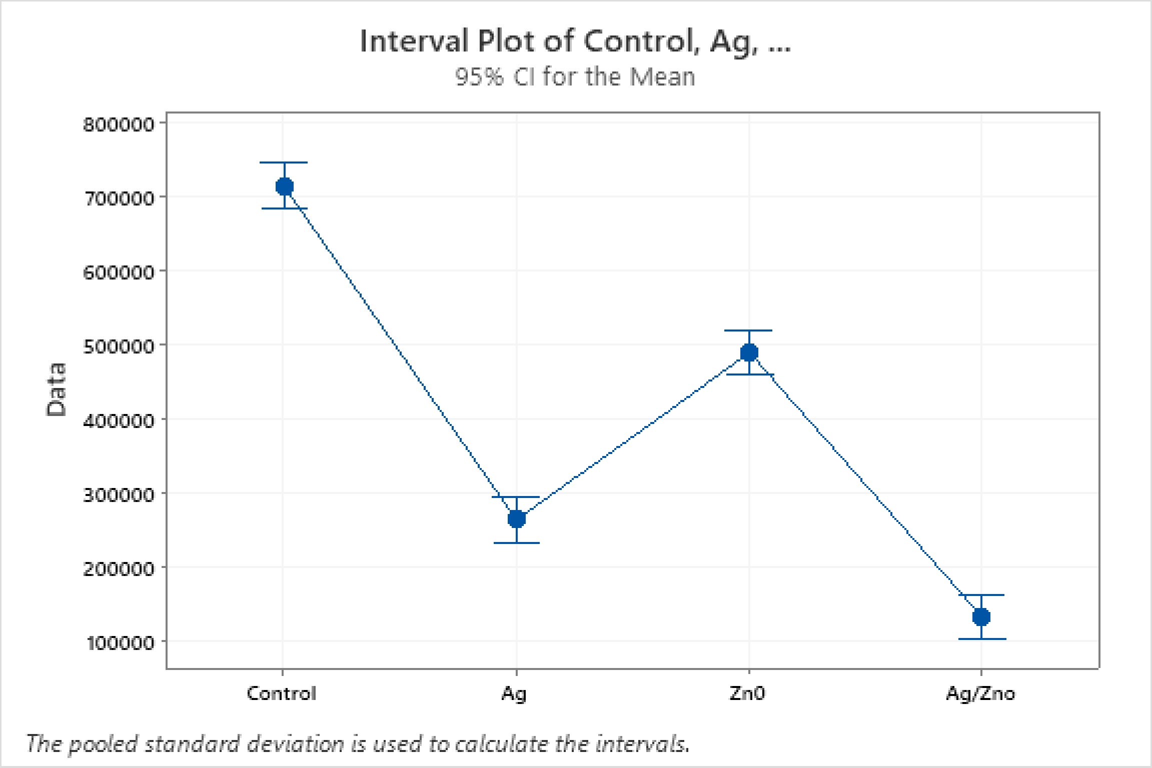
Interval Plot of Control, Ag, ...
95% CI for the Mean
800000
700000
600000
500000
400000
300000
200000
100000
Data
Control
Ag
Zn0
Ag/Zno
The pooled standard deviation is used to calculate the intervals.

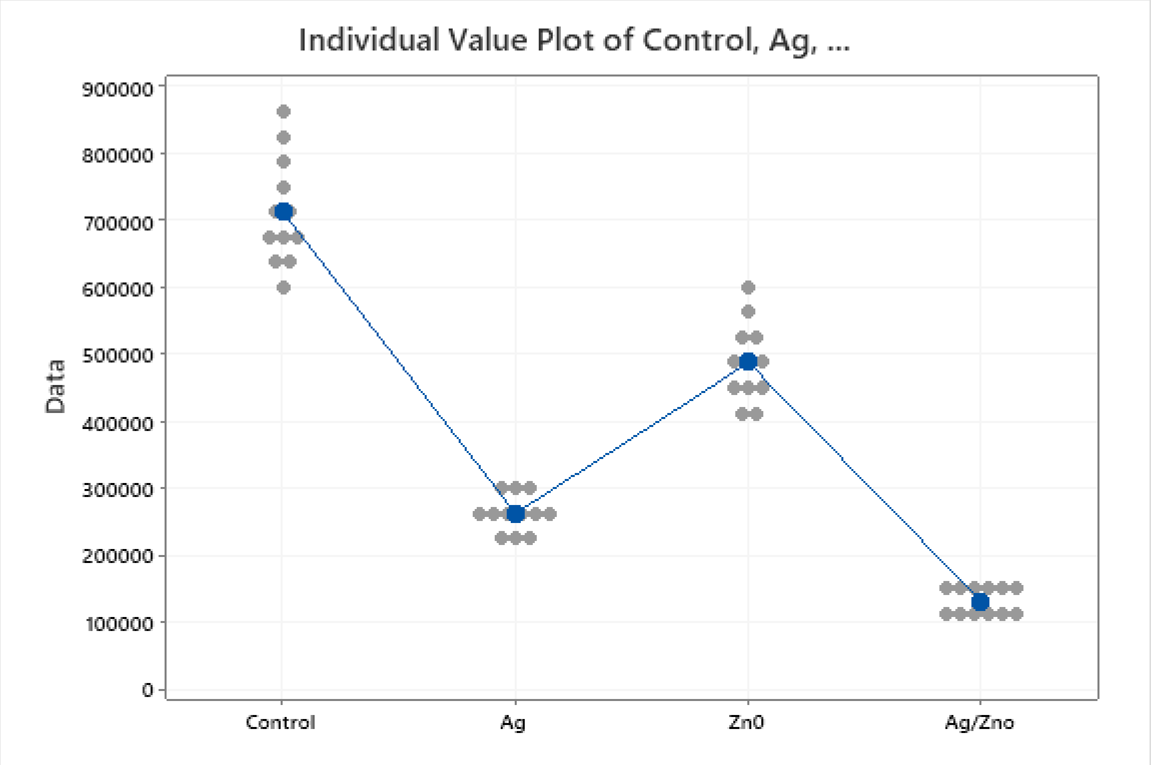
Individual Value Plot of Control, Ag, ...
Data
900000
800000
700000
600000
500000
400000
300000
200000
100000
0
Control
Ag
Zn0
Ag/Zno

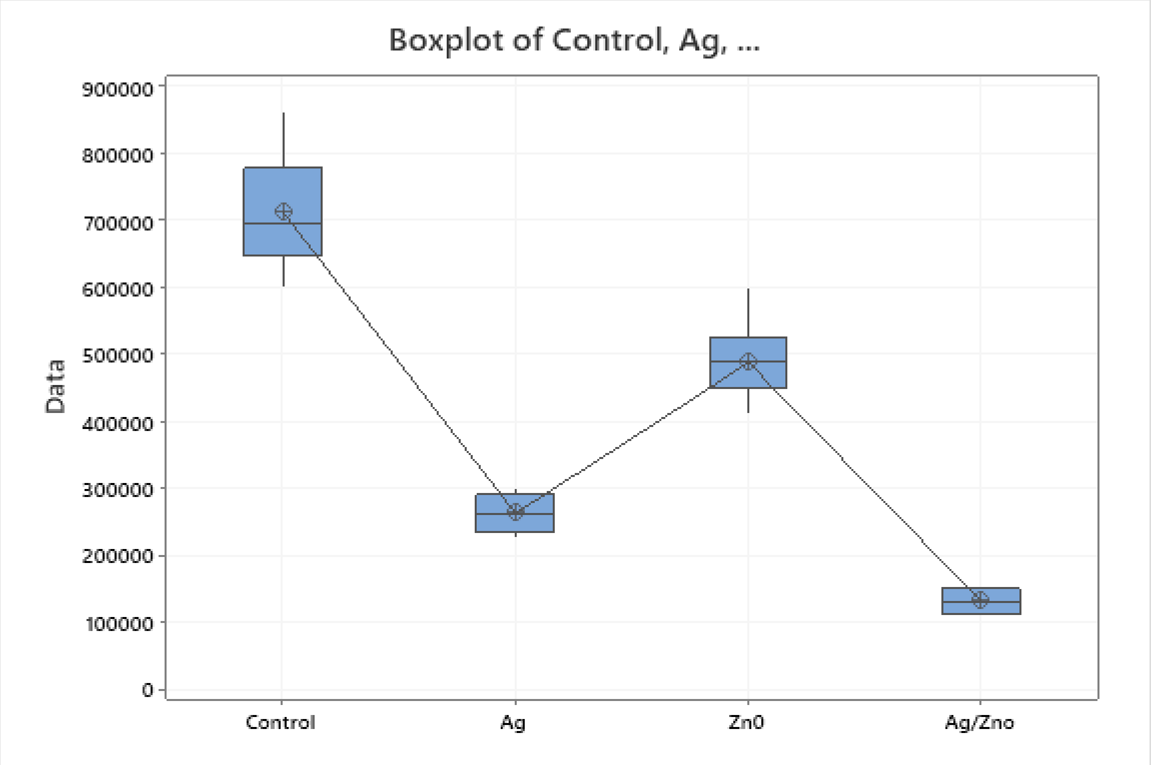
Boxplot of Control, Ag, ...
Data
900000
800000
700000
600000
500000
400000
300000
200000
100000
0
Control
Ag
Zn0
Ag/Zno

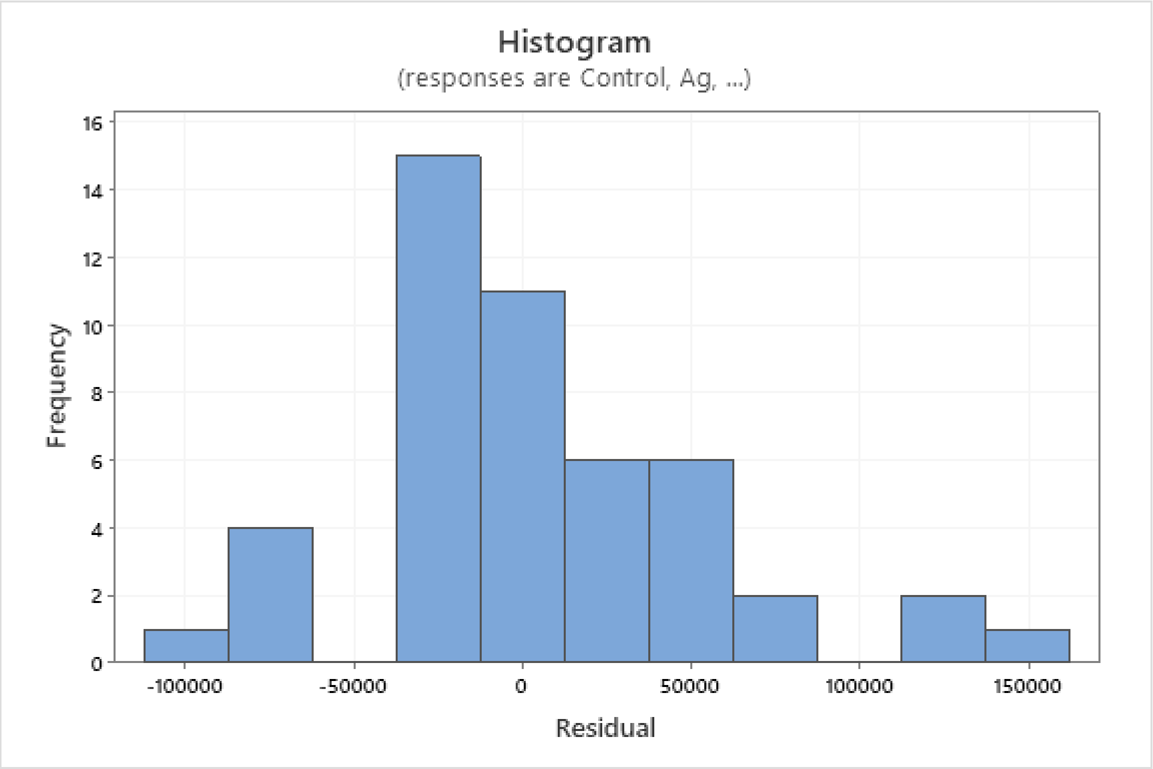
Histogram
(responses are Control, Ag, ...)
Frequency
Residual
-100000
-50000
0
50000
100000
150000
0
2
4
6
8
10
12
14
16
