# Supplementary material for: The antibacterial effect of silver, zinc-oxide and combination of silver/ zinc oxide nanoparticles coating of orthodontic brackets (an in vitro study)
Source: BMC Oral Health. 2022 Jun 9;22:230. doi: 10.1186/s12903-022-02263-6 (PMC9185939; doi:10.1186/s12903-022-02263-6)
Supplement: Supplementary file 6 — Additional file 6: CFU at T1 vs T2 for ZnO coated group on S. mutans. [file 12903_2022_2263_MOESM6_ESM.pdf]

WORKSHEET 1

Paired T-Test and CI: zno_ST_T1, zno_ST_T2

Descriptive Statistics

Sample	N	Mean	StDev	SE Mean
zno_ST_T1	12	11575000	630814	182100
zno_ST_T2	12	11220000	997287	287892

Estimation for Paired Difference

Mean	StDev	SE Mean	95% CI for μ_difference
355000	1450489	418720	(-566596, 1276596)

μ_difference: population mean of (zno_ST_T1 - zno_ST_T2)

Test

Null hypothesis	H_0: μ_difference = 0
Alternative hypothesis	H_1: μ_difference ≠ 0

T-Value	P-Value
0.85	0.415

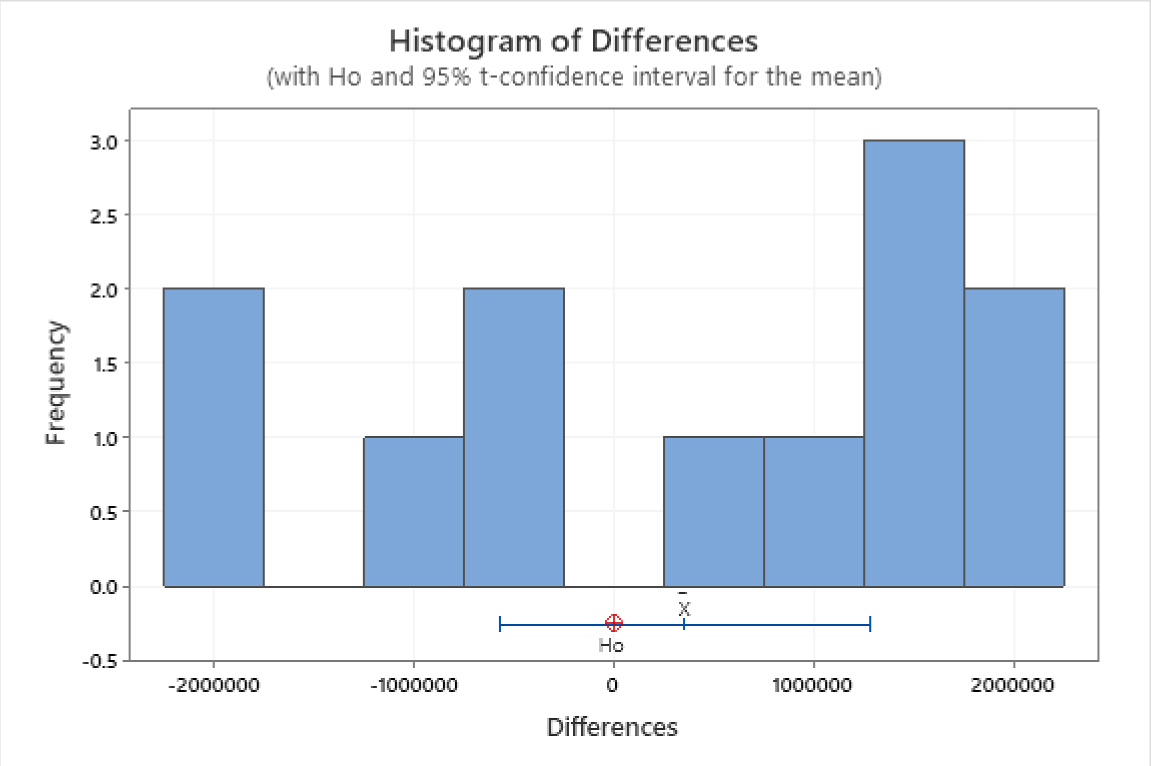

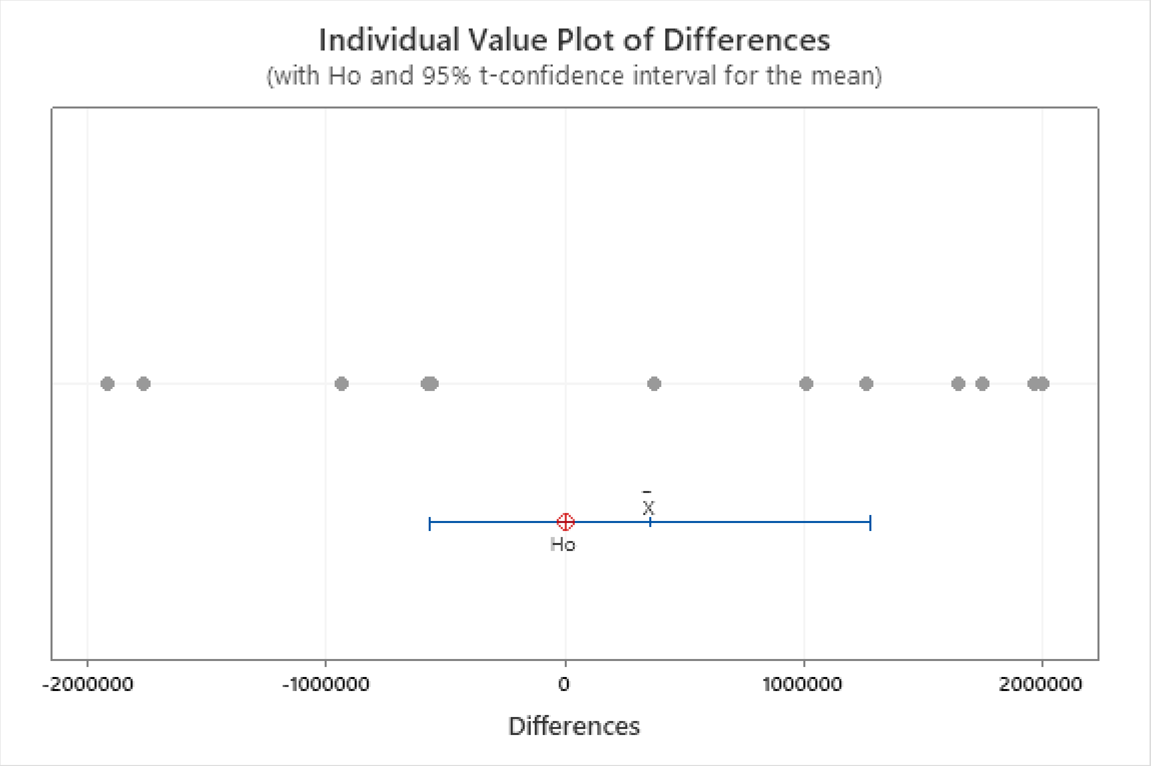
Individual Value Plot of Differences
(with Ho and 95% t-confidence interval for the mean)
X
Ho
-2000000
-1000000
0
1000000
2000000
Differences

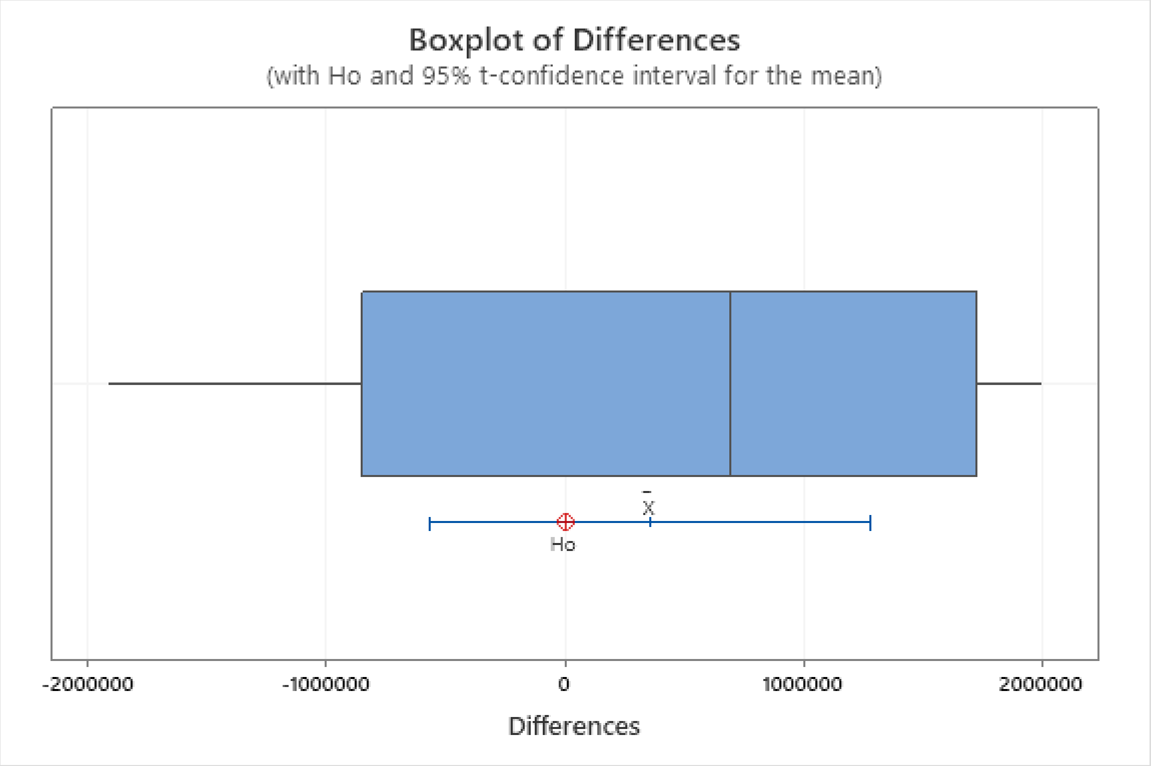
Boxplot of Differences
(with Ho and 95% t-confidence interval for the mean)
X
Ho
-2000000
-1000000
0
1000000
2000000
Differences
