# Supplementary material for: The antibacterial effect of silver, zinc-oxide and combination of silver/ zinc oxide nanoparticles coating of orthodontic brackets (an in vitro study)
Source: BMC Oral Health. 2022 Jun 9;22:230. doi: 10.1186/s12903-022-02263-6 (PMC9185939; doi:10.1186/s12903-022-02263-6)
Supplement: Supplementary file 7 — Additional file 7: CFU at T1 vs T2 for Ag/ ZnO coated group on S. mutans. [file 12903_2022_2263_MOESM7_ESM.pdf]

WORKSHEET 1

Paired T-Test and CI: zno+Ag_strept_T1, zno+Ag_strepto_T2

Descriptive Statistics

Sample	N	Mean	StDev	SE Mean
zno+Ag_strept_T1	12	7675000	738376	213151
zno+Ag_strepto_T2	12	7520000	705717	203723

Estimation for Paired Difference

Mean	StDev	SE Mean	95% CI for μ_difference
155000	528771	152643	(-180965, 490965)

μ_difference: population mean of (zno+Ag_strept_T1 - zno+Ag_strepto_T2)

Test

Null hypothesis	H_0: μ_difference = 0
Alternative hypothesis	H_1: μ_difference ≠ 0

T-Value	P-Value
1.02	0.332

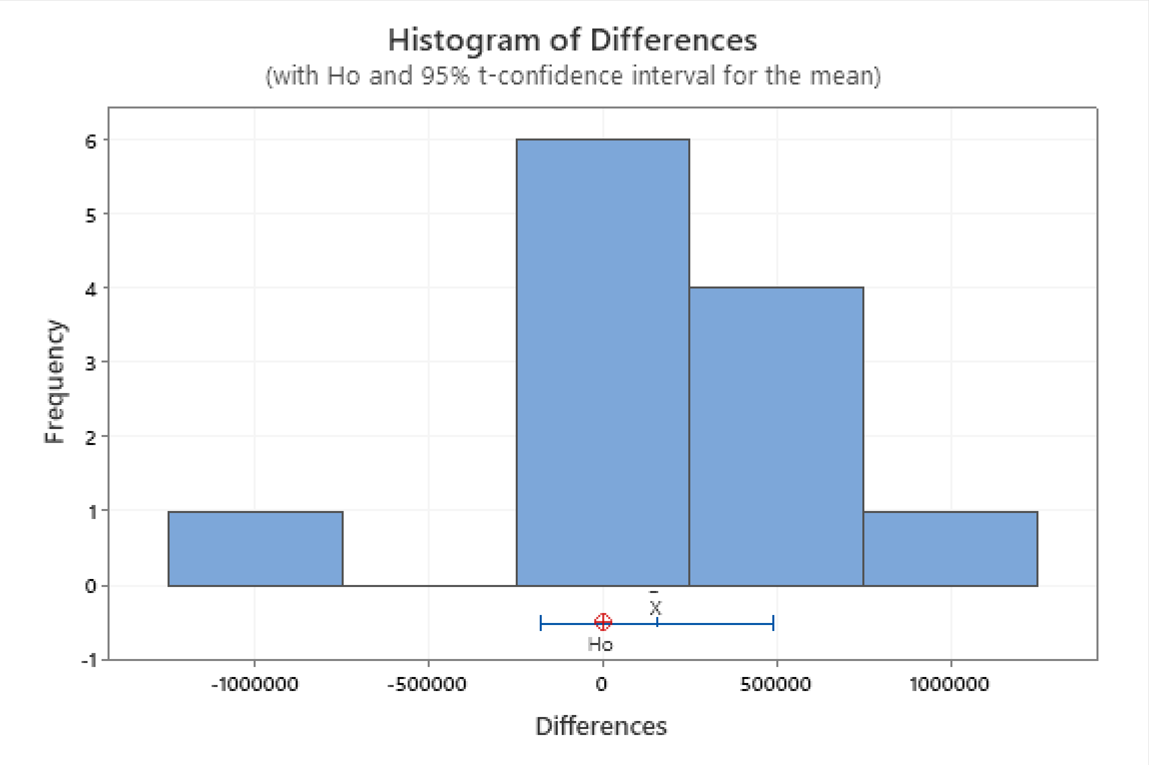

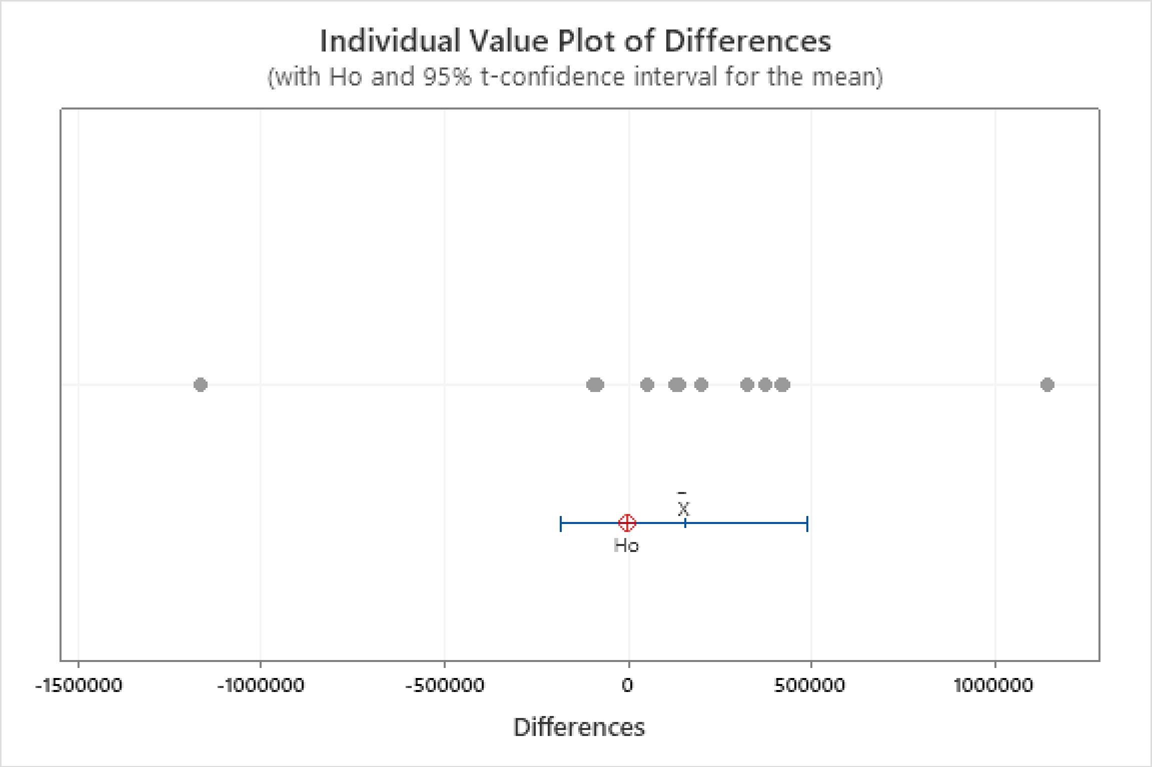
Individual Value Plot of Differences
(with Ho and 95% t-confidence interval for the mean)
X̄
Ho
-1500000
-1000000
-500000
0
500000
1000000
Differences

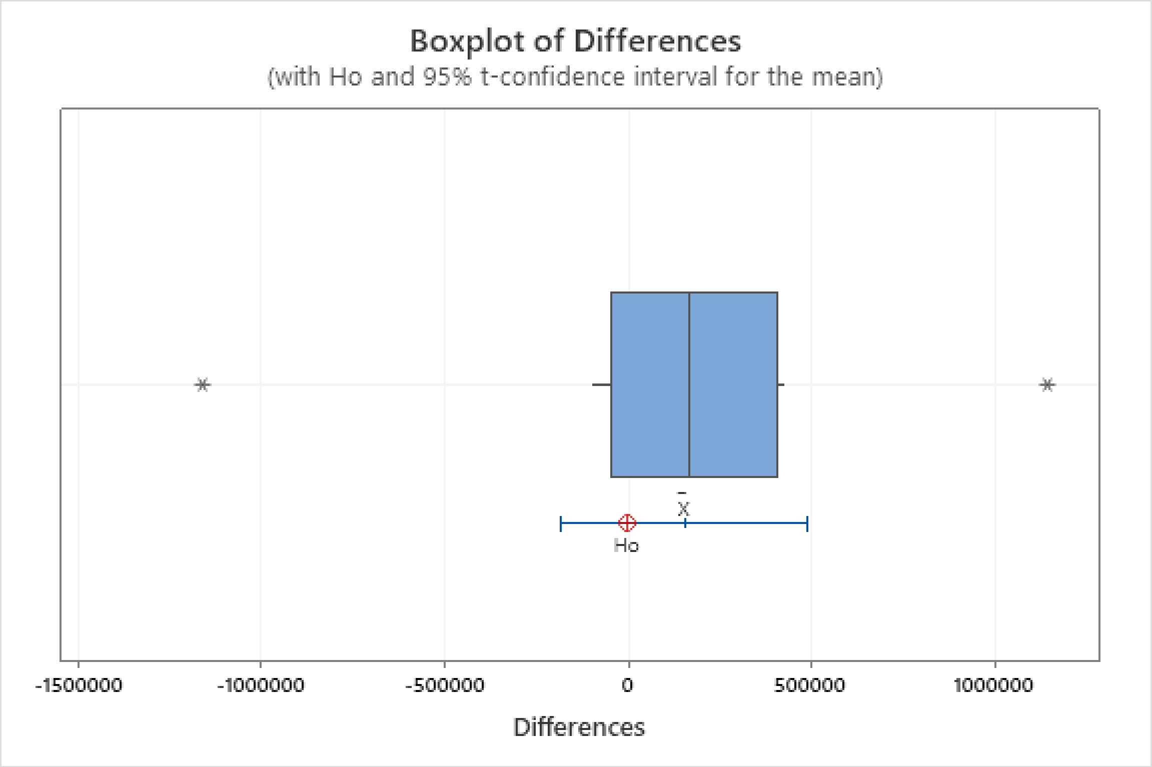
Boxplot of Differences
(with Ho and 95% t-confidence interval for the mean)
X̄
Ho
-1500000
-1000000
-500000
0
500000
1000000
Differences
