# Supplementary material for: The antibacterial effect of silver, zinc-oxide and combination of silver/ zinc oxide nanoparticles coating of orthodontic brackets (an in vitro study)
Source: BMC Oral Health. 2022 Jun 9;22:230. doi: 10.1186/s12903-022-02263-6 (PMC9185939; doi:10.1186/s12903-022-02263-6)
Supplement: Supplementary file 8 — Additional file 8: Percent of inhibition at T1 vs T2 for Ag coated group on S. mutans. [file 12903_2022_2263_MOESM8_ESM.pdf]

WORKSHEET 1

Paired T-Test and CI: Ag_strept_T1, Ag_strepto_T2

Descriptive Statistics

Sample	N	Mean	StDev	SE Mean
Ag_strept_T1	12	27.60	2.00	0.58
Ag_strepto_T2	12	24.08	6.89	1.99

Estimation for Paired Difference

Mean	StDev	SE Mean	95% CI for μ_difference
3.52	7.42	2.14	(-1.19, 8.24)

μ_difference: population mean of (Ag_strept_T1 - Ag_strepto_T2)

Test

Null hypothesis	H_0: μ_difference = 0
Alternative hypothesis	H_1: μ_difference ≠ 0

T-Value	P-Value
1.64	0.128

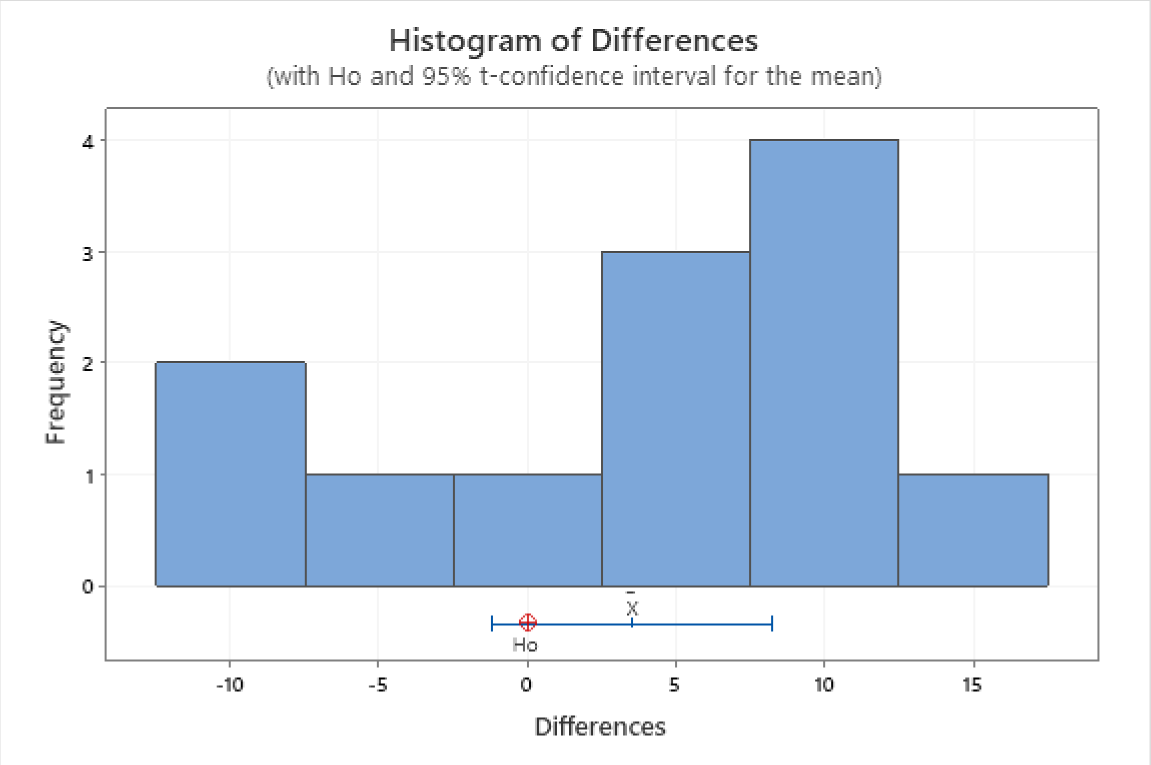

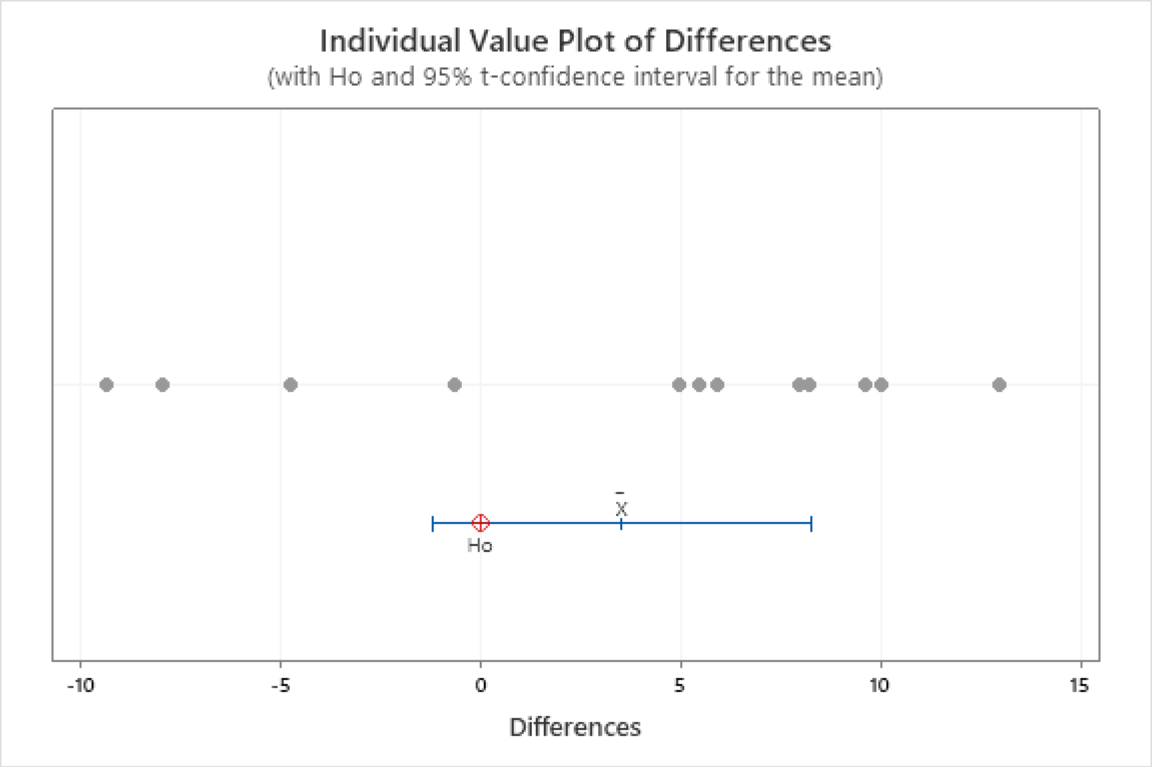
Individual Value Plot of Differences
(with Ho and 95% t-confidence interval for the mean)
X̄
Ho
-10
-5
0
5
10
15
Differences

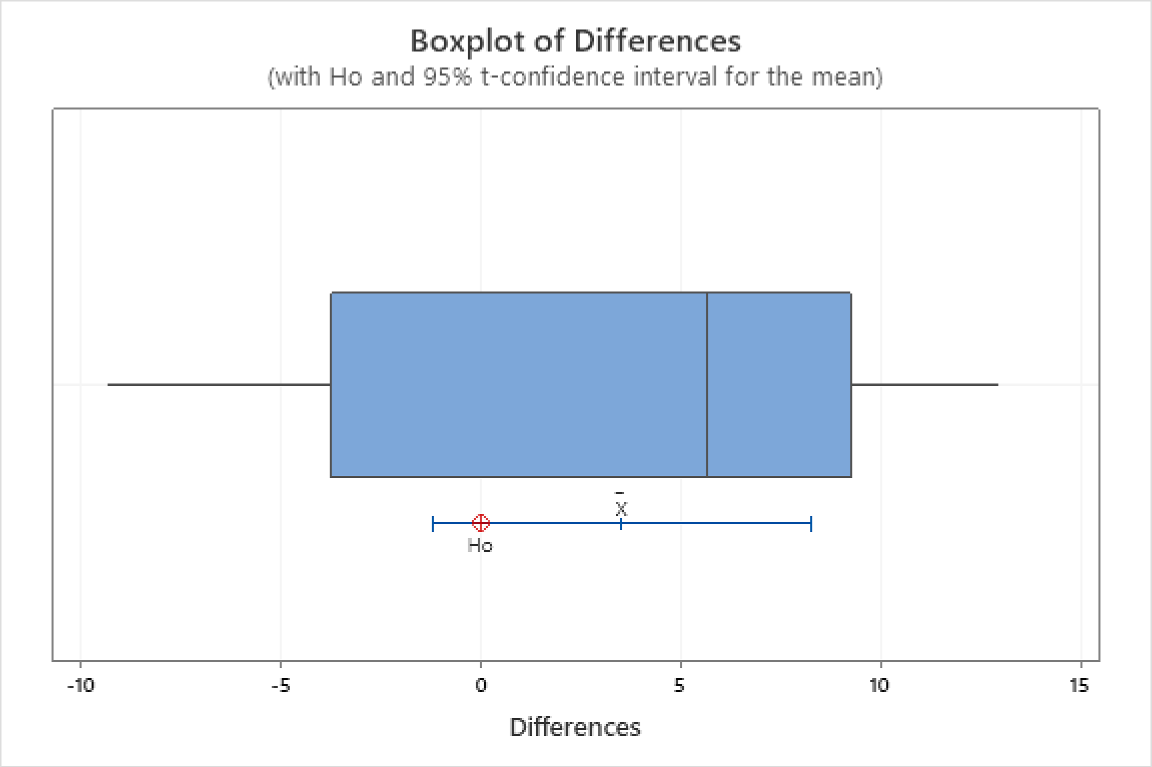
Boxplot of Differences
(with Ho and 95% t-confidence interval for the mean)
X̄
Ho
-10
-5
0
5
10
15
Differences
