# Supplementary material for: The antibacterial effect of silver, zinc-oxide and combination of silver/ zinc oxide nanoparticles coating of orthodontic brackets (an in vitro study)
Source: BMC Oral Health. 2022 Jun 9;22:230. doi: 10.1186/s12903-022-02263-6 (PMC9185939; doi:10.1186/s12903-022-02263-6)
Supplement: Supplementary file 9 — Additional file 9: Percent of inhibition at T1 vs T2 for ZnO coated group on S. mutans. [file 12903_2022_2263_MOESM9_ESM.pdf]

WORKSHEET 1

Paired T-Test and CI: zno_strept_T1, zno_strepto_T2

Descriptive Statistics

Sample	N	Mean	StDev	SE Mean
zno_strept_T1	12	17.54	4.49	1.30
zno_strepto_T2	12	15.38	7.52	2.17

Estimation for Paired Difference

Mean	StDev	SE Mean	95% CI for μ_difference
2.16	10.72	3.10	(-4.66, 8.97)

μ_difference: population mean of (zno_strept_T1 - zno_strepto_T2)

Test

Null hypothesis	H_0: μ_difference = 0
Alternative hypothesis	H_1: μ_difference ≠ 0

T-Value	P-Value
0.70	0.500

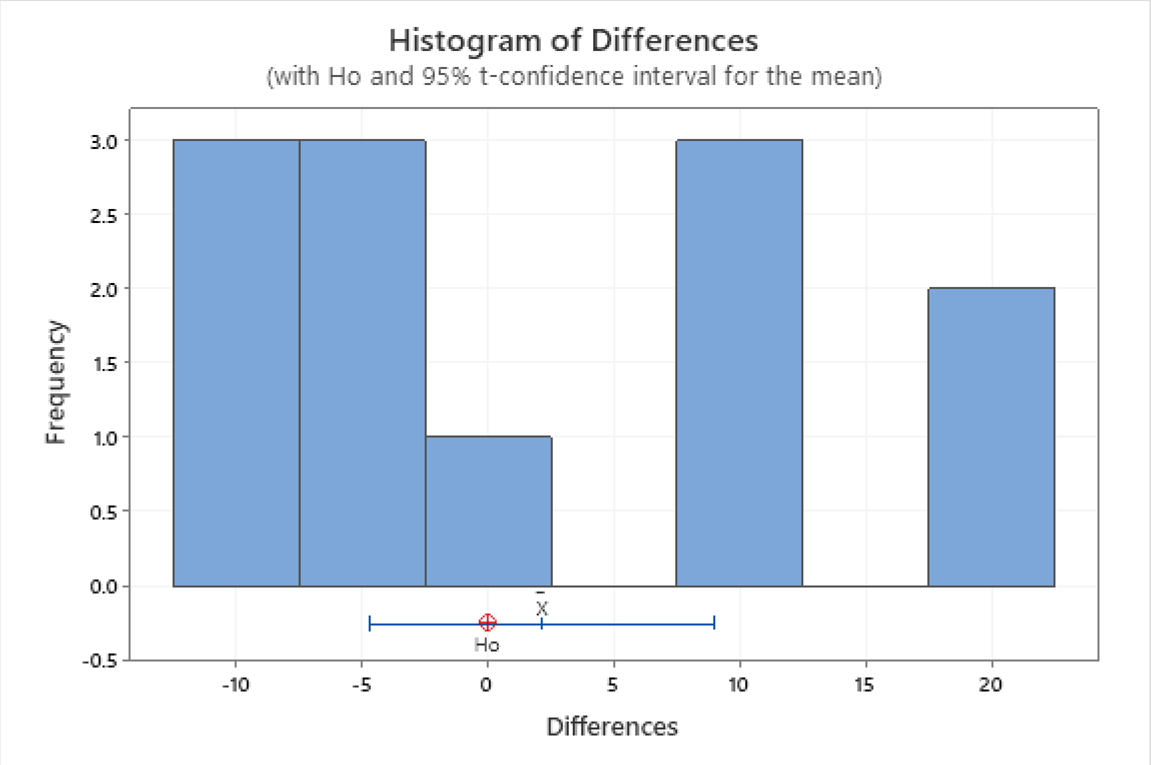

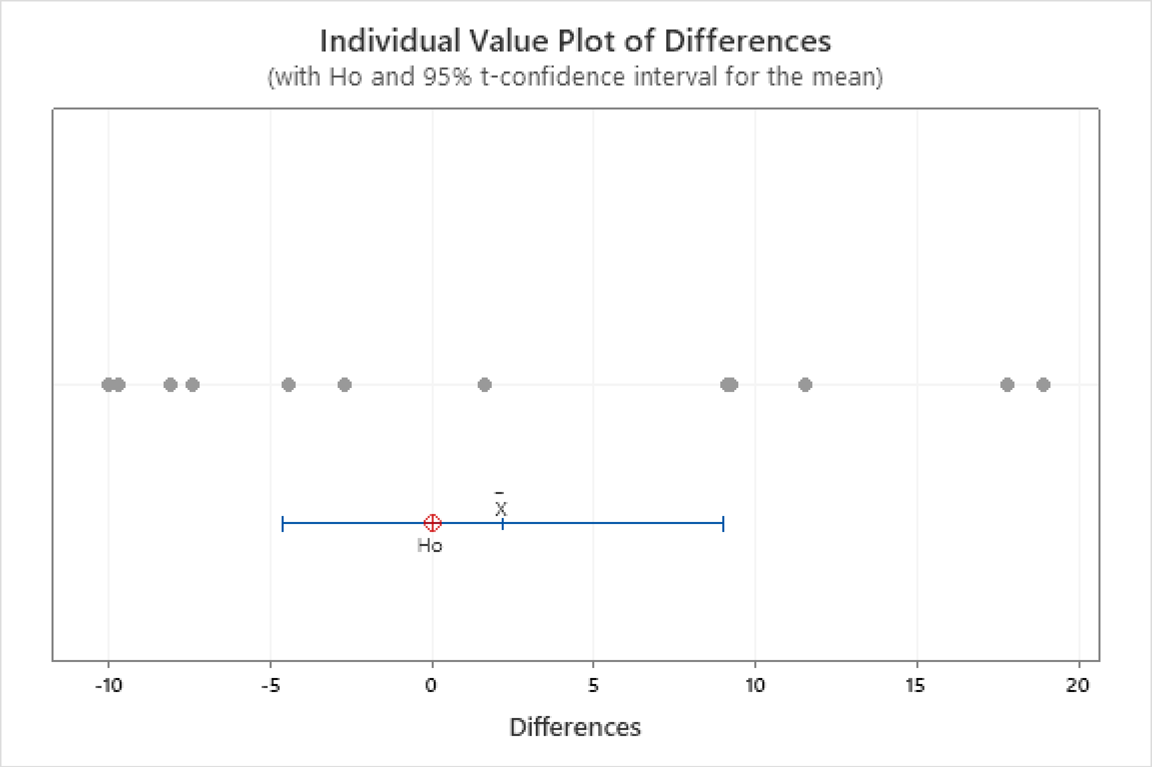
Individual Value Plot of Differences
(with Ho and 95% t-confidence interval for the mean)
X̄
Ho
-10
-5
0
5
10
15
20
Differences

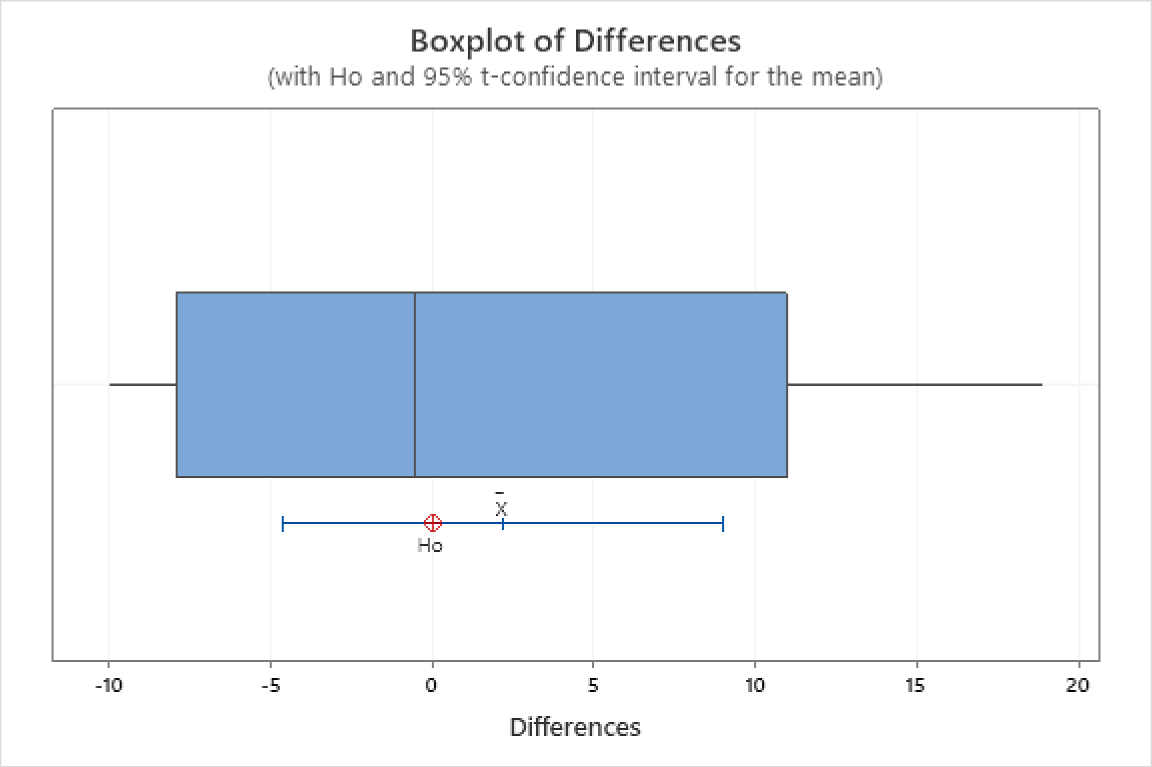
Boxplot of Differences
(with Ho and 95% t-confidence interval for the mean)
X̄
Ho
-10
-5
0
5
10
15
20
Differences
