# Supplementary material for: The antibacterial effect of silver, zinc-oxide and combination of silver/ zinc oxide nanoparticles coating of orthodontic brackets (an in vitro study)
Source: BMC Oral Health. 2022 Jun 9;22:230. doi: 10.1186/s12903-022-02263-6 (PMC9185939; doi:10.1186/s12903-022-02263-6)
Supplement: Supplementary file 12 — Additional file 12: CFU at T1 vs T2 for ZnO coated group on L. acidophilus. [file 12903_2022_2263_MOESM12_ESM.pdf]

WORKSHEET 1

Paired T-Test and CI: zno_lacto_T1, zno _lacto_T2

Descriptive Statistics

Sample	N	Mean	StDev	SE Mean
zno_lacto_T1	12	462500	58387	16855
zno _lacto_T2	12	487500	57653	16643

Estimation for Paired Difference

Mean	StDev	SE Mean	95% CI for μ_difference
-25000	86603	25000	(-80025, 30025)

μ_difference: population mean of (zno_lacto_T1 - zno _lacto_T2)

Test

Null hypothesis	H_0: μ_difference = 0
Alternative hypothesis	H_1: μ_difference ≠ 0

T-Value	P-Value
-1.00	0.339

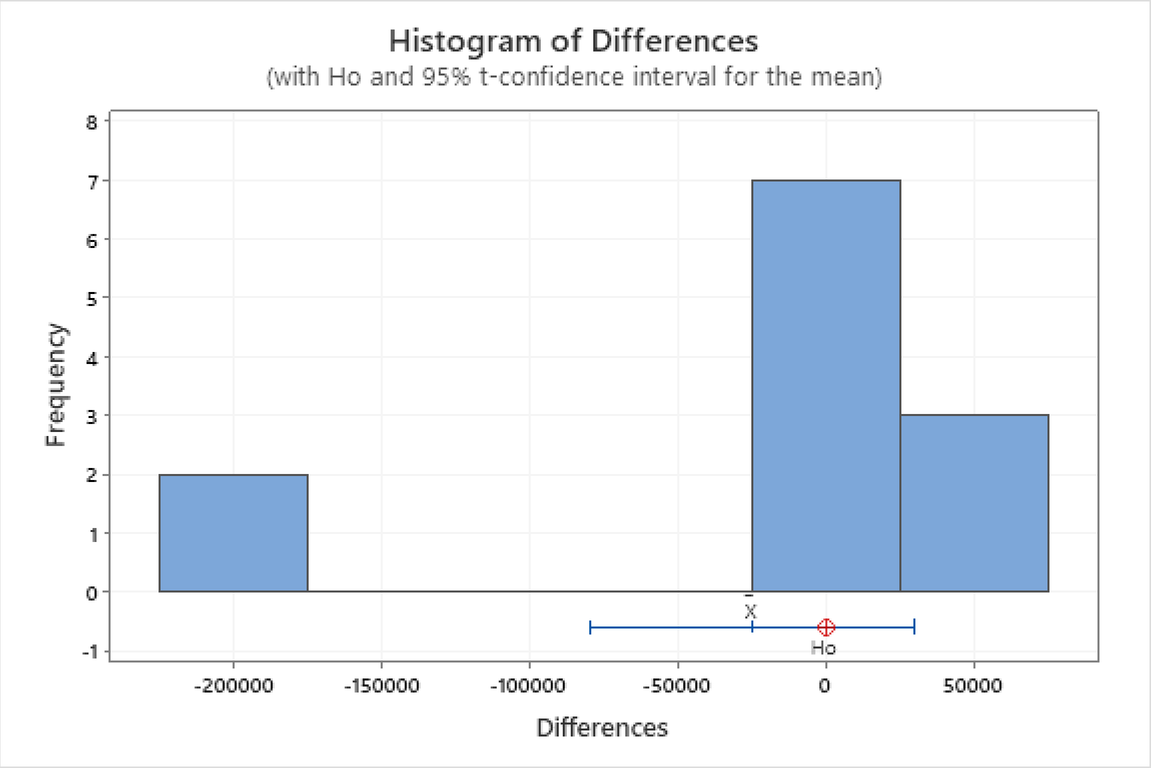

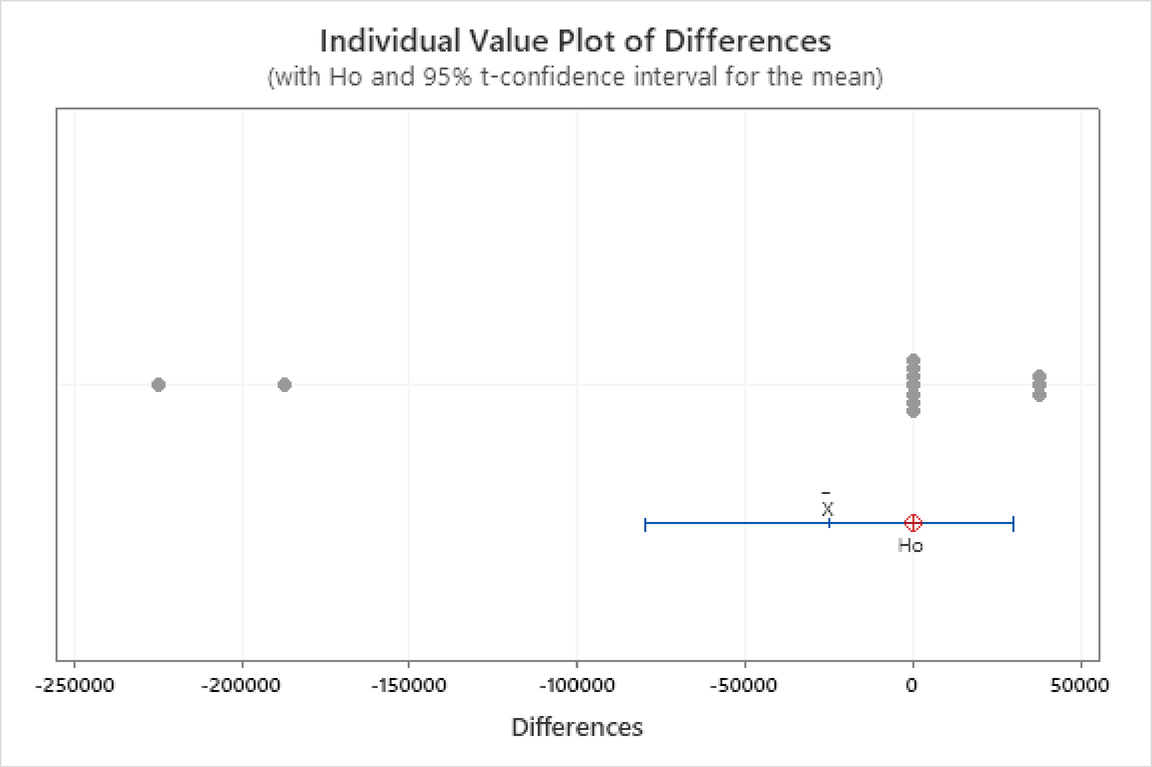
Individual Value Plot of Differences
(with Ho and 95% t-confidence interval for the mean)
X̄
Ho
-250000
-200000
-150000
-100000
-50000
0
50000
Differences

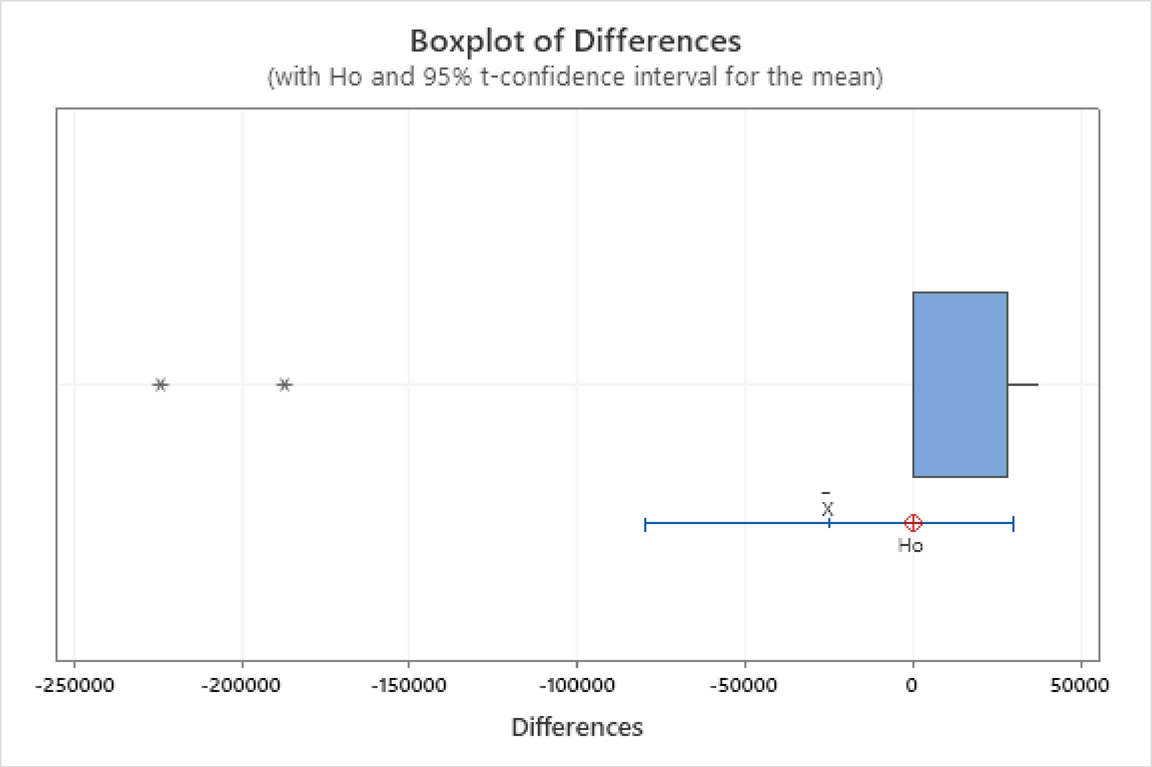
Boxplot of Differences
(with Ho and 95% t-confidence interval for the mean)
X̄
Ho
-250000
-200000
-150000
-100000
-50000
0
50000
Differences
