# Supplementary material for: The antibacterial effect of silver, zinc-oxide and combination of silver/ zinc oxide nanoparticles coating of orthodontic brackets (an in vitro study)
Source: BMC Oral Health. 2022 Jun 9;22:230. doi: 10.1186/s12903-022-02263-6 (PMC9185939; doi:10.1186/s12903-022-02263-6)
Supplement: Supplementary file 13 — Additional file 13: CFU at T1 vs T2 for Ag/ ZnO coated group on L. acidophilus. [file 12903_2022_2263_MOESM13_ESM.pdf]

WORKSHEET 1

Paired T-Test and CI: zno+Ag_lacto_T1, zno+Ag _lacto_T2

Descriptive Statistics

Sample	N	Mean	StDev	SE Mean
zno+Ag_lacto_T1	12	128125	19310	5574
zno+Ag _lacto_T2	12	131250	19584	5653

Estimation for Paired Difference

Mean	StDev	SE Mean	95% CI for μ_difference
-3125	25071	7237	(-19054, 12804)

μ_difference: population mean of (zno+Ag_lacto_T1 - zno+Ag _lacto_T2)

Test

Null hypothesis	H_0: μ_difference = 0
Alternative hypothesis	H_1: μ_difference ≠ 0

T-Value	P-Value
-0.43	0.674

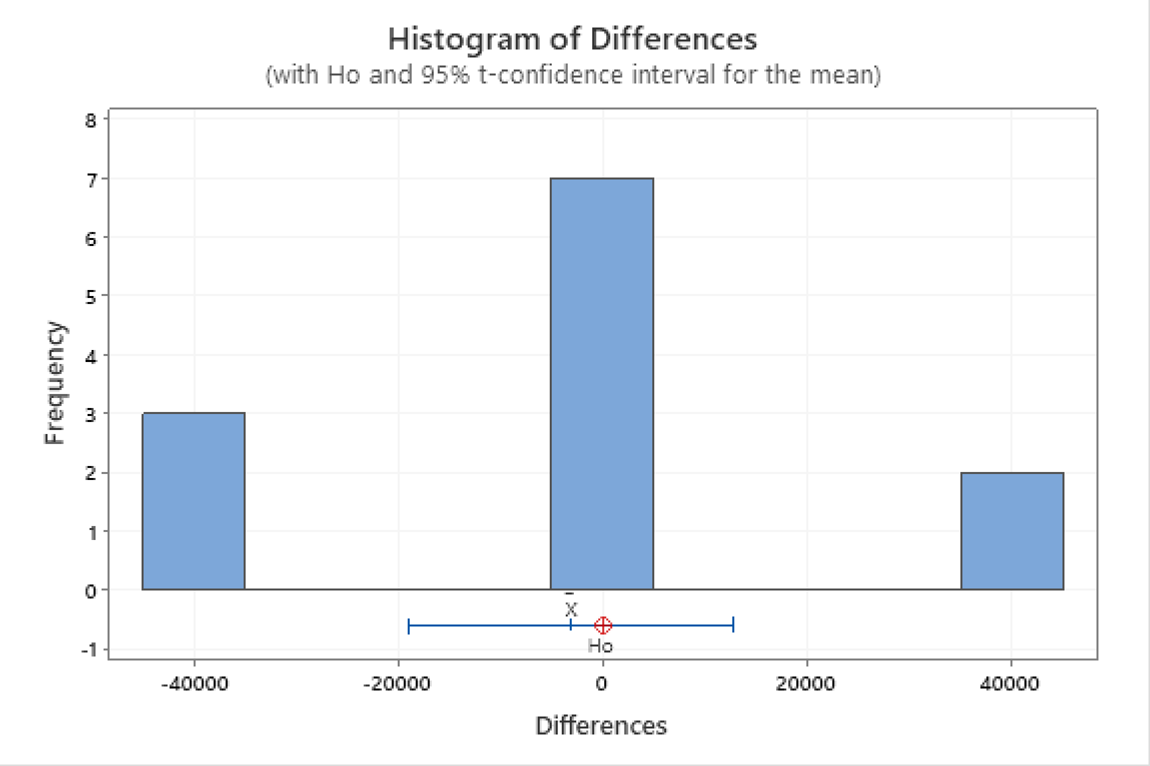

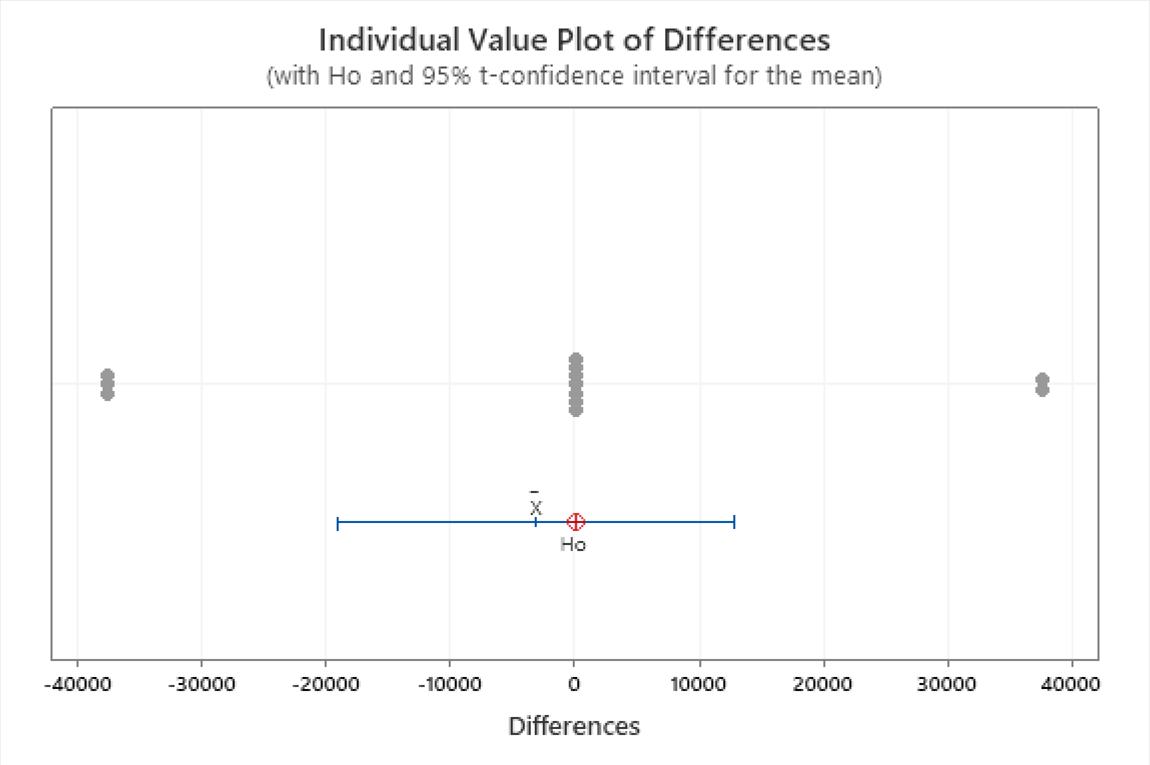
Individual Value Plot of Differences
(with Ho and 95% t-confidence interval for the mean)
X
Ho
-40000
-30000
-20000
-10000
0
10000
20000
30000
40000
Differences

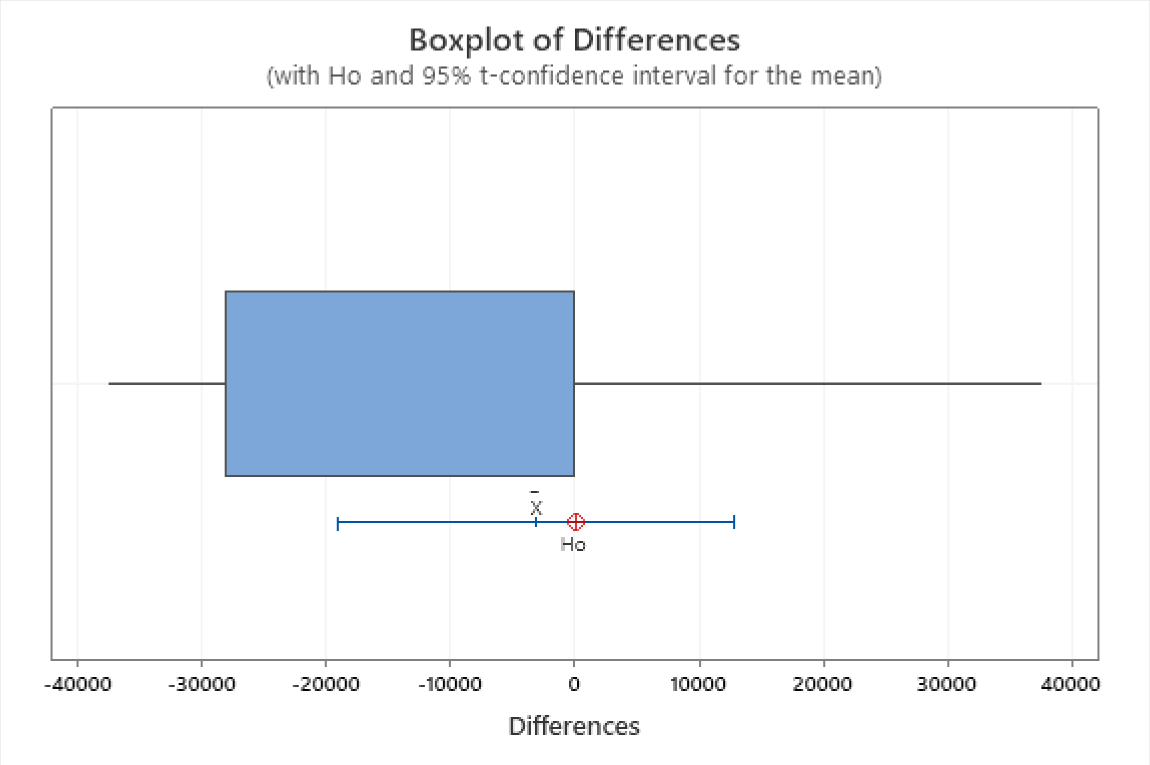
Boxplot of Differences
(with Ho and 95% t-confidence interval for the mean)
X
Ho
-40000
-30000
-20000
-10000
0
10000
20000
30000
40000
Differences
