# Supplementary material for: The antibacterial effect of silver, zinc-oxide and combination of silver/ zinc oxide nanoparticles coating of orthodontic brackets (an in vitro study)
Source: BMC Oral Health. 2022 Jun 9;22:230. doi: 10.1186/s12903-022-02263-6 (PMC9185939; doi:10.1186/s12903-022-02263-6)
Supplement: Supplementary file 14 — Additional file 14: Percent of inhibition at T1 vs T2 for Ag coated group on L. acidophilus. [file 12903_2022_2263_MOESM14_ESM.pdf]

WORKSHEET 1

Paired T-Test and CI: Ag_lacto_T1, Ag _lacto_T2

Descriptive Statistics

Sample	N	Mean	StDev	SE Mean
Ag_lacto_T1	12	62.02	5.19	1.50
Ag _lacto_T2	12	63.16	3.89	1.12

Estimation for Paired Difference

Mean	StDev	SE Mean	95% CI for μ_difference
-1.14	5.57	1.61	(-4.68, 2.40)

μ_difference: population mean of (Ag_lacto_T1 - Ag _lacto_T2)

Test

Null hypothesis	H_0: μ_difference = 0
Alternative hypothesis	H_1: μ_difference ≠ 0

T-Value	P-Value
-0.71	0.494

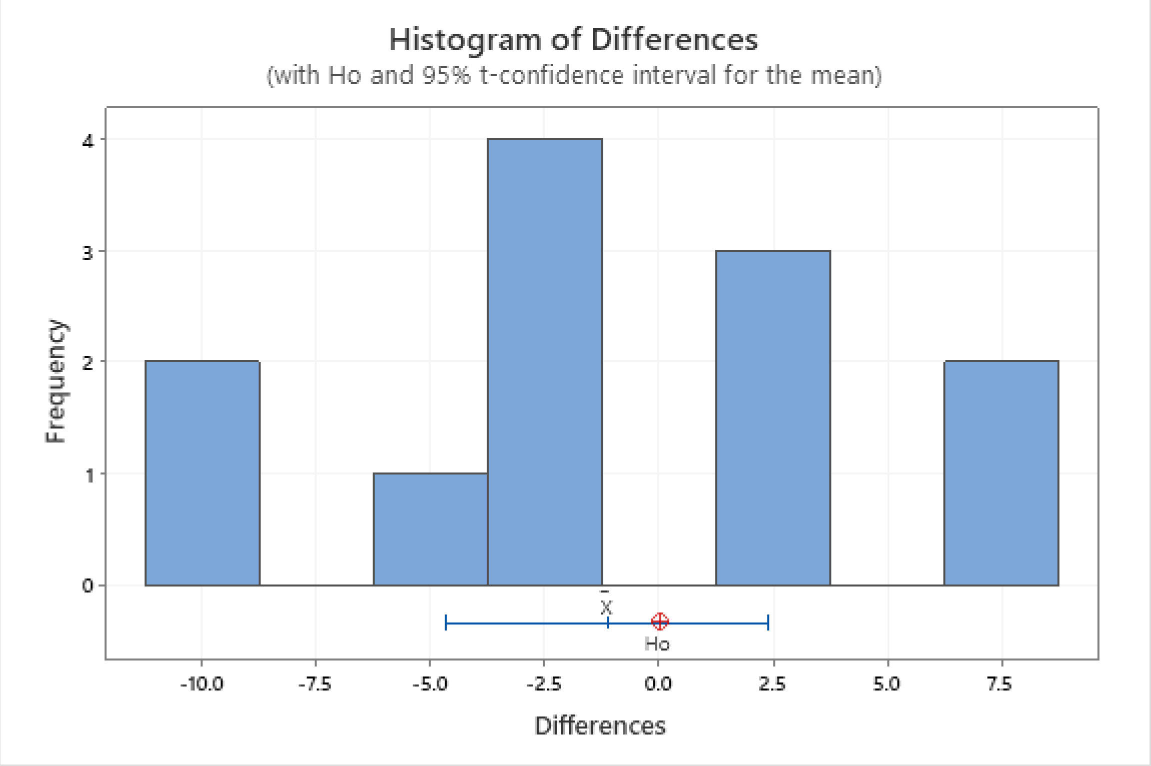

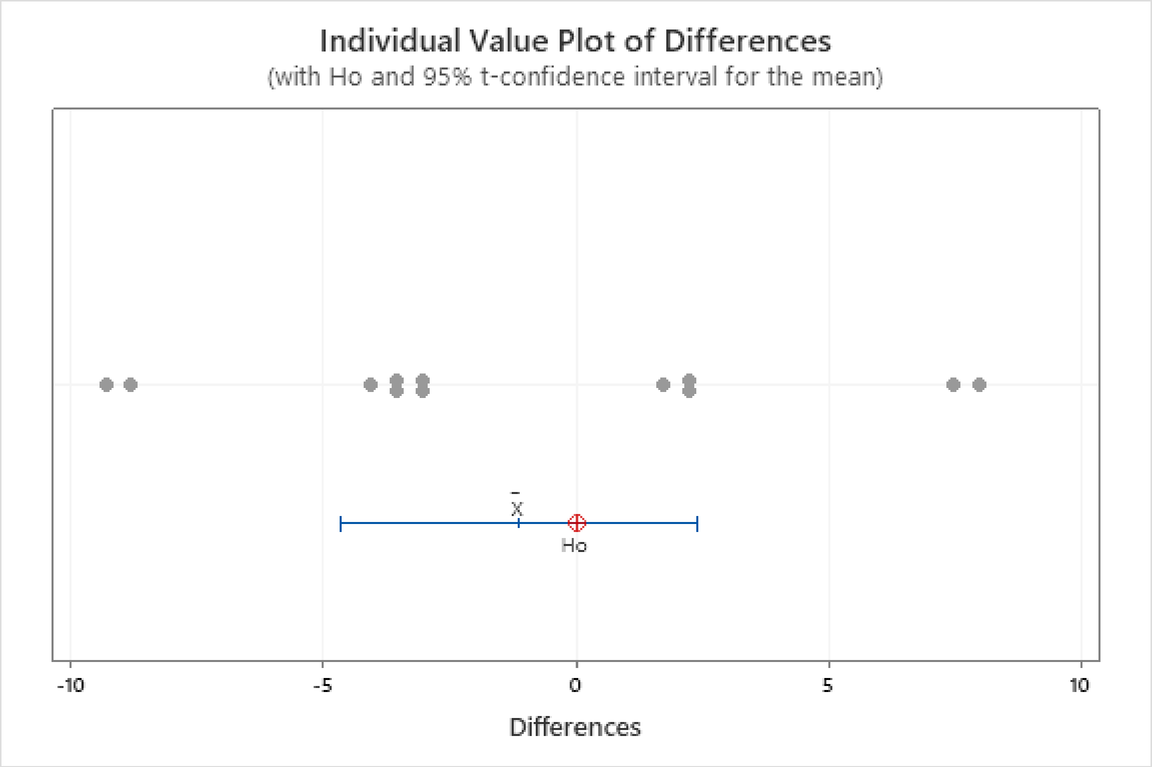
Individual Value Plot of Differences
(with Ho and 95% t-confidence interval for the mean)
X̄
Ho
-10
-5
0
5
10
Differences

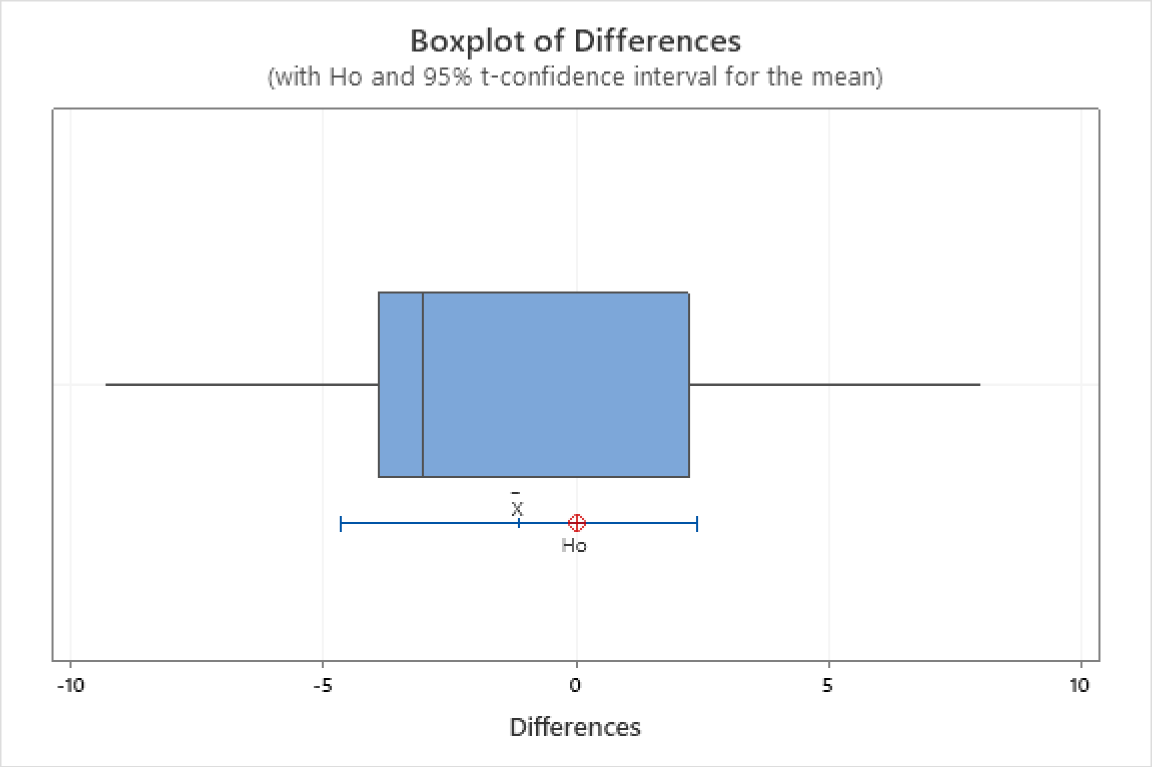
Boxplot of Differences
(with Ho and 95% t-confidence interval for the mean)
X̄
Ho
-10
-5
0
5
10
Differences
