# Supplementary material for: The antibacterial effect of silver, zinc-oxide and combination of silver/ zinc oxide nanoparticles coating of orthodontic brackets (an in vitro study)
Source: BMC Oral Health. 2022 Jun 9;22:230. doi: 10.1186/s12903-022-02263-6 (PMC9185939; doi:10.1186/s12903-022-02263-6)
Supplement: Supplementary file 15 — Additional file 15: Percent of inhibition at T1 vs T2 for ZnO coated group on L. acidophilus. [file 12903_2022_2263_MOESM15_ESM.pdf]

WORKSHEET 1

Paired T-Test and CI: Zno_lacto_T1, Zno_lacto_T2

Descriptive Statistics

Sample	N	Mean	StDev	SE Mean
Zno_lacto_T1	12	28.85	8.98	2.59
Zno_lacto_T2	12	31.58	8.09	2.34

Estimation for Paired Difference

Mean	StDev	SE Mean	95% CI for μ_difference
-2.73	12.76	3.68	(-10.84, 5.37)

μ_difference: population mean of (Zno_lacto_T1 - Zno_lacto_T2)

Test

Null hypothesis	H_0: μ_difference = 0
Alternative hypothesis	H_1: μ_difference ≠ 0

T-Value	P-Value
-0.74	0.474

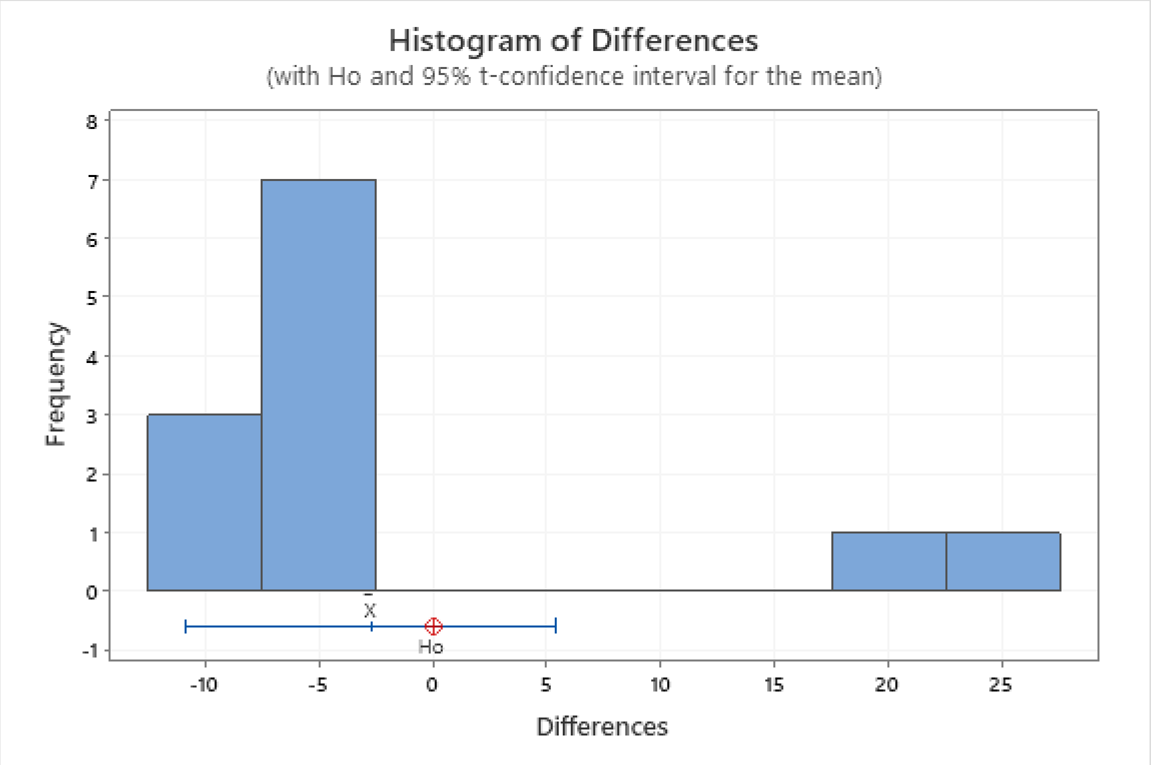

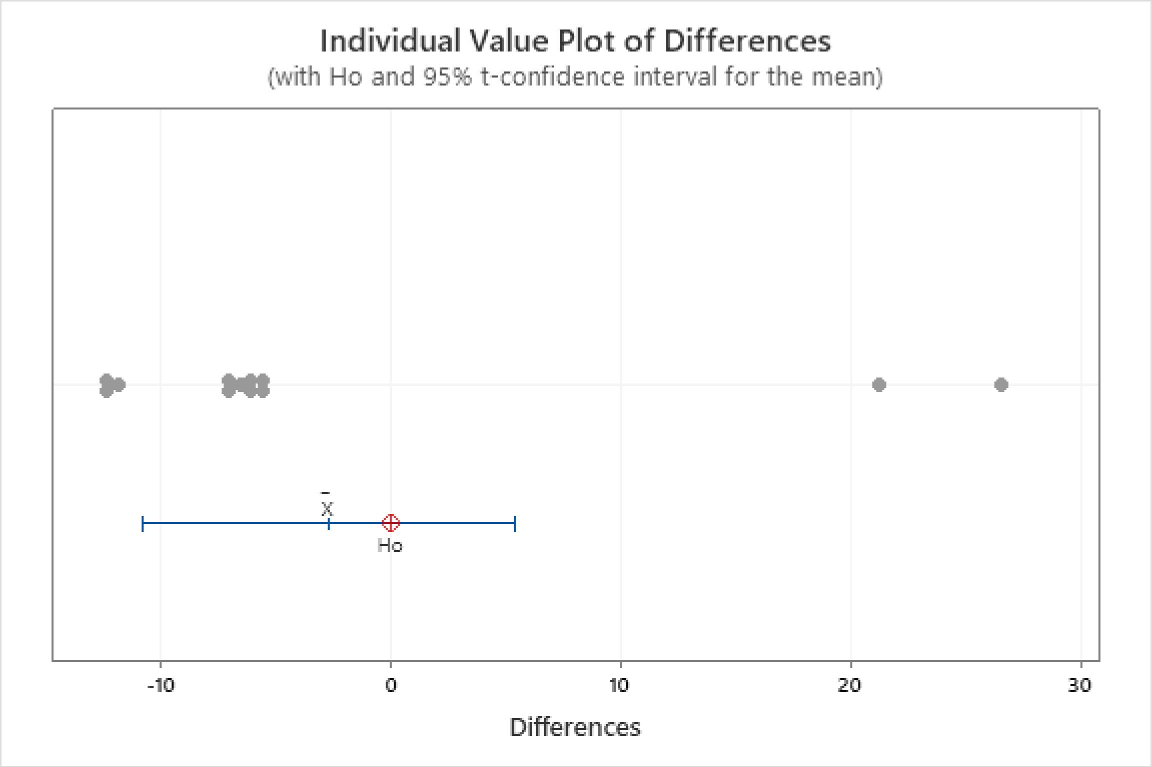
Individual Value Plot of Differences
(with Ho and 95% t-confidence interval for the mean)
X̄
Ho
-10
0
10
20
30
Differences

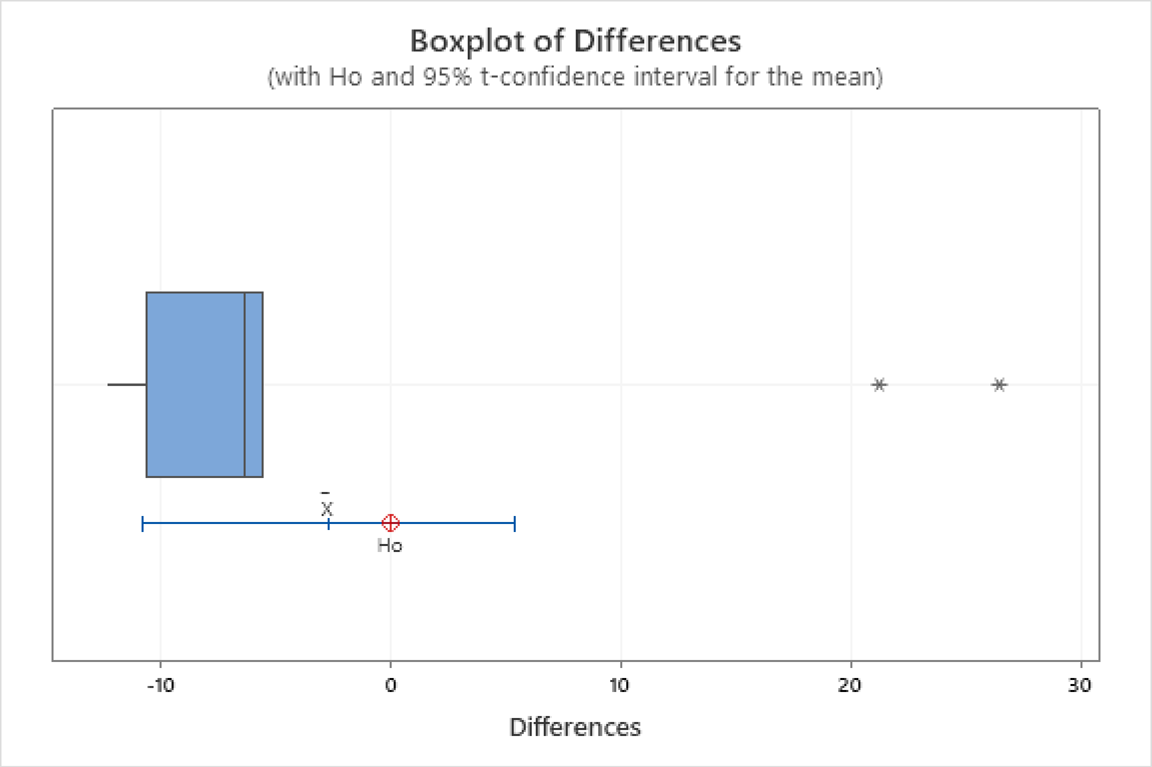
Boxplot of Differences
(with Ho and 95% t-confidence interval for the mean)
X̄
Ho
-10
0
10
20
30
Differences
