# Supplementary material for: The antibacterial effect of silver, zinc-oxide and combination of silver/ zinc oxide nanoparticles coating of orthodontic brackets (an in vitro study)
Source: BMC Oral Health. 2022 Jun 9;22:230. doi: 10.1186/s12903-022-02263-6 (PMC9185939; doi:10.1186/s12903-022-02263-6)
Supplement: Supplementary file 16 — Additional file 16: Percent of inhibition at T1 vs T2 for Ag/ ZnO coated group on L. acidophilus. [file 12903_2022_2263_MOESM16_ESM.pdf]

WORKSHEET 1

Paired T-Test and CI: Zno+Ag_lacto_T1, Zno+Ag_lacto_T2

Descriptive Statistics

Sample	N	Mean	StDev	SE Mean
Zno+Ag_lacto_T1	12	80.288	2.971	0.858
Zno+Ag_lacto_T2	12	81.579	2.749	0.793

Estimation for Paired Difference

Mean	StDev	SE Mean	95% CI for μ_difference
-1.29	3.69	1.07	(-3.64, 1.05)

μ_difference: population mean of (Zno+Ag_lacto_T1 - Zno+Ag_lacto_T2)

Test

Null hypothesis	H_0: μ_difference = 0
Alternative hypothesis	H_1: μ_difference ≠ 0

T-Value	P-Value
-1.21	0.251

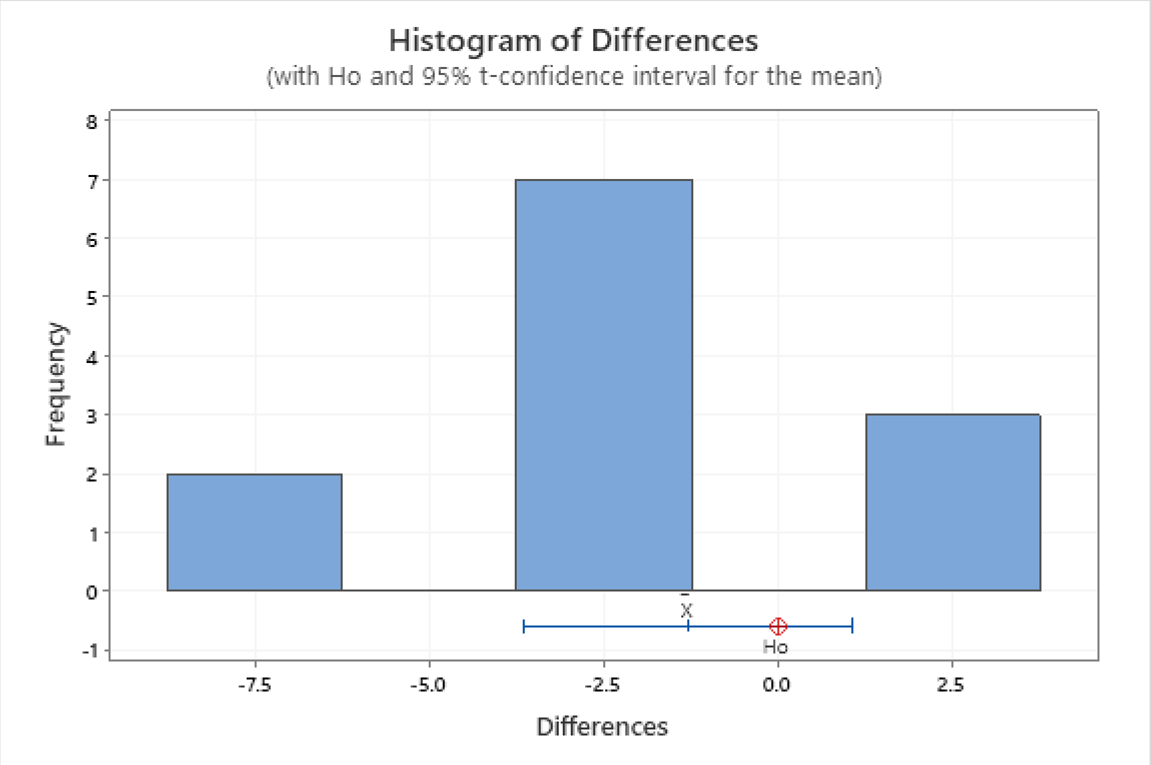

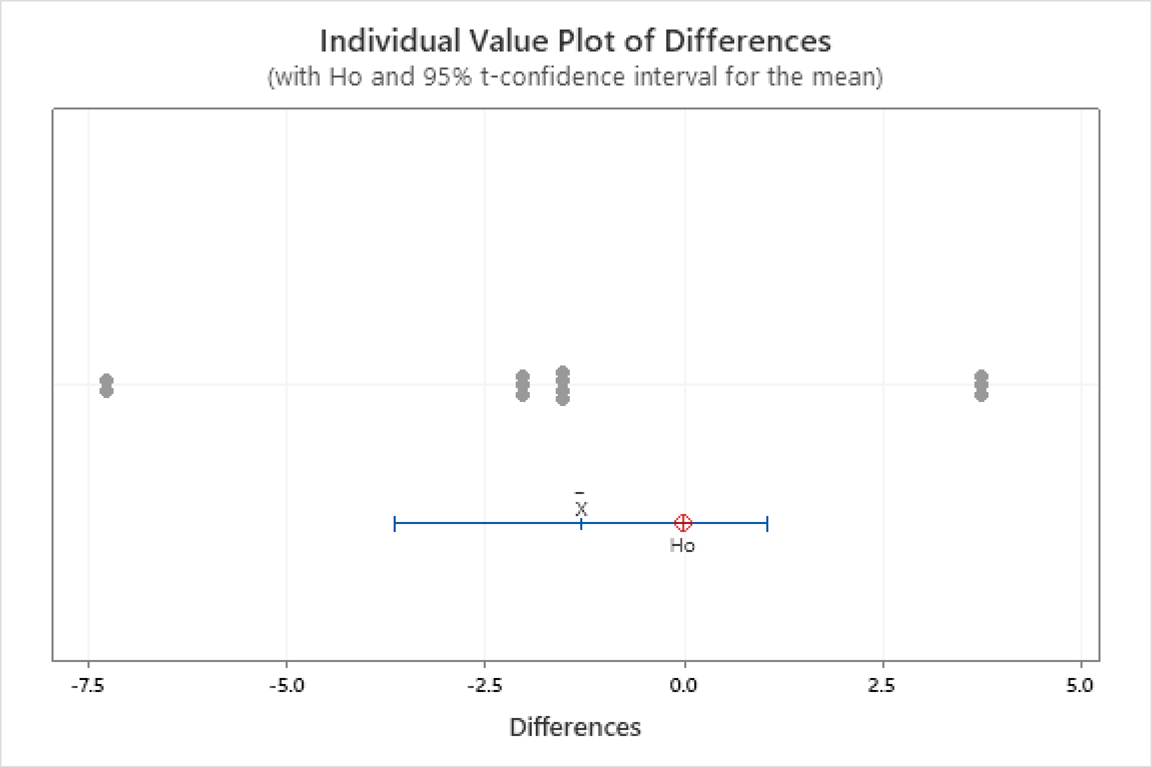
Individual Value Plot of Differences
(with Ho and 95% t-confidence interval for the mean)
X̄
Ho
-7.5
-5.0
-2.5
0.0
2.5
5.0
Differences

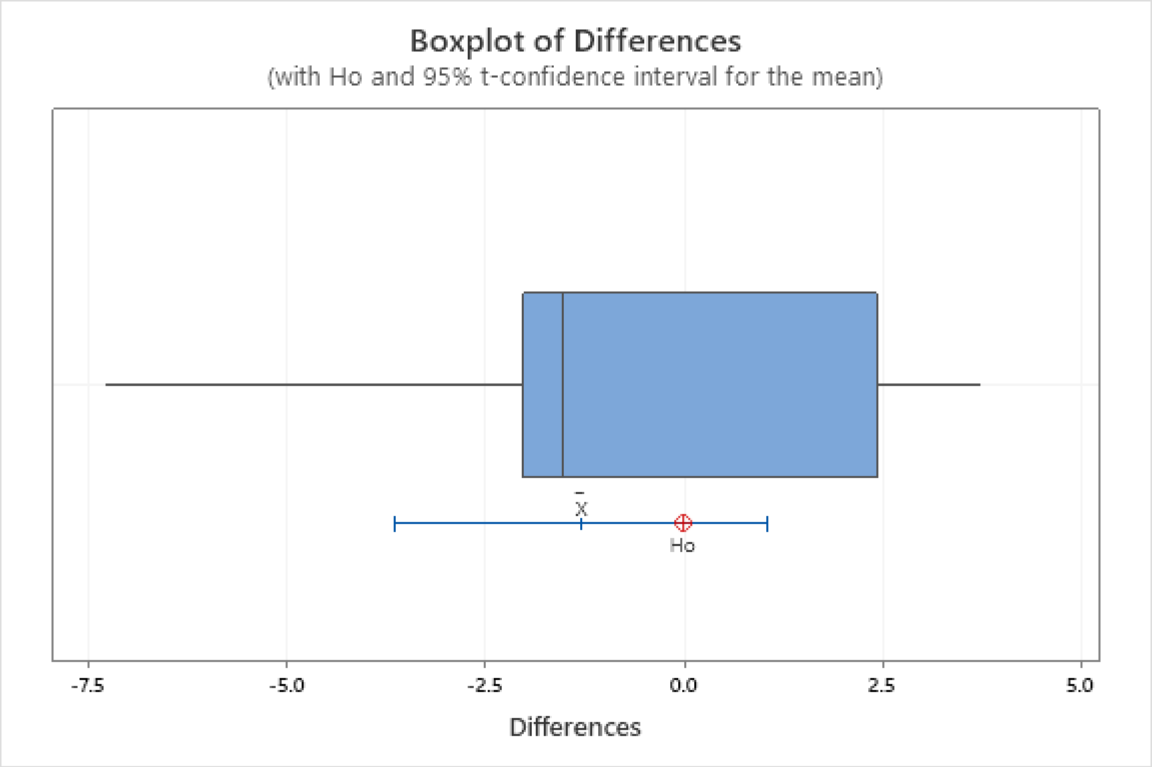
Boxplot of Differences
(with Ho and 95% t-confidence interval for the mean)
X̄
Ho
-7.5
-5.0
-2.5
0.0
2.5
5.0
Differences
